# Mixed-methods analysis on psychedelic-augmented meditation experiences from a randomized controlled mindfulness retreat

**DOI:** 10.1038/s41598-026-39261-5

**Published:** 2026-03-18

**Authors:** Jonas T. T. Schlomberg, Daniel Meling, Robin Grylka, Emilia A. Vasella, Dominik Augustinovic, Milan Scheidegger

**Affiliations:** 1 Psychedelic Research and Therapy Development, Department of Adult Psychiatry and Psychotherapy, Psychiatric University Clinic Zurich and University of Zurich, Zurich, Switzerland; 2 Digital Society Initiative, University of Zurich, Zurich, Switzerland; 3 Department of Psychosomatic Medicine and Psychotherapy, Medical Center—University of Freiburg, Faculty of Medicine, University of Freiburg, Freiburg, Germany; 4 Neuroscience Center Zurich, Swiss Federal Institute of Technology Zurich, University of Zurich, Zurich, Switzerland

**Keywords:** Psychedelics, Meditation, Natural language processing, Subjective effects, Multi-Modal, Synergistic effects, Health care, Psychology, Psychology

## Abstract

The acute subjective effects (ASEs) of psychedelic substances are assumed to play a critical role for their therapeutic and well-being enhancing benefits. However, recent work voiced critique regarding the validity and adequacy of conventional measures and modalities utilized to study ASEs of psychedelics, and call for data-driven, unbiased, and experience-based research approaches. The emergence of advanced Natural Language Processing techniques as an enabler of data-driven qualitative research holds promise for addressing the current biases and limitations in the investigation of ASEs of psychedelics. In the present study, we employed an NLP-driven, multi-method analytical paradigm to study the subjective experiences of participants in an ecologically valid RCT examining the effect of DMT/harmine on meditative states in experienced meditators using phenomenological interviews. Our analysis showed differences in the thematic landscape and experiential diversity of meditation under placebo and meditation under DMT-harmine while showing overlap in their semantic topographies. The mixed-modal analysis successfully identified a wide range of well-established primary subjective effects while also detecting subtle, patterned regularities in language that traditional hypothesis-driven approaches alone may overlook. It revealed a pronounced use of Buddhist concepts and spiritual jargon to describe and integrate the subjective experience, independent of the experimental condition. Findings suggested shared experiential features between meditative and psychedelic states, a strong drug-context interconnection and potential synergistic effects of meditation and psychedelics. We advocate for using NLP-augmented, data-driven paradigms to deepen the understanding of psychedelic subjectivity and emphasize the importance of extra-pharmacological factors in shaping therapeutic outcomes.

**Supplementary Information:**

The online version contains supplementary material available at 10.1038/s41598-026-39261-5.

## Introduction

The acute subjective effects (ASEs) of hallucinogenic psychedelic substances, such as dimethyltryptamine (DMT), lysergic acid diethylamide (LSD) or psilocybin (“magic mushrooms”), are inherently multifaceted, rich and complex phenomena central to the altered states of consciousness (ASCs) they induce in humans^1–4^. Despite a strong correlation between psychometric assessments of acute psychedelic effects and positive therapeutic responses weeks or months after the experience^3,5–7^, there is still much debate as to whether ASEs are instrumental to therapeutic change or merely epiphenomenal side-effects of psychoplastogenic mechanisms^3,4,8^.

Meditation-induced ASCs have been described in recent research as phenomenologically similar to psychedelic states of consciousness^9–11^. At the same time, the wellbeing and mental health promoting properties of different meditation practices have been established in several randomized controlled studies^12,13^. First studies suggest combined effects of meditation and psychedelics^11,14,15^, indicating that the investigation of combined-interventions holds promise for advancing the understanding of ASEs therapeutic importance.

Standardized psychometric tools such as the Mystical Experience Questionnaire (MEQ)^16,17^ or the 5-Dimensions Altered States of Consciousness questionnaire (5-D ASC)^18^ have been the dominant methods to assess ASEs. However, these scales are increasingly criticized for their conceptual limitations and biases, such as being outdated^19^, lacking heterogeneity^2,20^ and construct validity^21,22^ and their reliance on researcher-specified cognitive and experiential categories^23,24^. These instruments may not fully reveal the rich phenomenological diversity of psychedelic experiences, because they fail to capture first-person meaning-making processes – processes that require approaches for studying language from within the experience^10^. Recent studies showed that the subjective effects of different psychedelics were comparable across various psychometric scales^25,26^, even though large-scale, text-based quantitative analyses had found reliable semantic and phenomenological variations^19,27,28^. This suggests that there are aspects of psychedelic experiences that are difficult to capture with itemized measures.

The resurgence of psychedelic research has been dominated by quantitative research modalities^29–31^. Sophisticated neuroimaging studies^32–35^ provide important insights into neural correlates of psychedelic experiences but only limited insight into their experiential quality. While they may track correlates of brain activity, they cannot resolve how to make meaning of these states – a factor likely relevant for therapeutic outcomes^36,37^. Consequently, approaches considering first-person experiential descriptions to assess the structure, content, and dynamics of psychedelic states could contribute to a more integrative understanding of psychedelics’ modes of action^38–40^.

Recent advances in Natural Language Processing (NLP) offer promising alternatives, allowing for large-scale, data-driven analysis of subjective narratives without presupposing theoretical frameworks^41–44^. Several studies have successfully utilized advanced NLP techniques to study the subjective effects of psychedelic substances^27,28,45–47^. For example, previous studies uncovered semantic structures that align with receptor binding profiles^28^, and compounds could be differentiated by their perceptual signatures based on quantitative linguistic markers of psychological states and processes^46^. More recently, Noah and colleagues used an embedding model from OpenAI to research the visual effects of different psychedelics. Their investigation showed that (a) the proportion of statements addressing visual effects, and (b) the types of reported visual effects varied significantly and consistently across psychedelic substances^27^.

Yet, these efforts have relied almost exclusively on a publicly available large-scale dataset consisting of unstructured, anecdotal self-reports from highly diverse non-clinical settings. The Dataset is maintained by the nonprofit organization Erowid Center^48^. The collected reports neither address differences of subjective effects between drug- and placebo-induced experiences nor do they cater to other design considerations of contemporary research trials. Consequently, it remains largely unknown how state-of-the-art NLP techniques perform on small- to mid-scale experimental data sets of psychedelic experience reports collected in a structured research setting.

To address these gaps, we employed a mixed-methods, NLP-augmented, qualitative analysis paradigm to structured phenomenological interviews conducted as part of a double-blind, placebo-controlled RCT held as a 3-day mindfulness retreat. Using BERTopic, a state-of-the-art natural language processing technique, we examined semantic patterns across interview transcripts from both the verum and the placebo group. This automated analysis was complemented by a manual, frequency-based and theory-informed thematic analysis to (a) cross-validate and (b) expand on the explorative findings from the NLP analysis.

Our objectives were threefold: (1) to assess the feasibility of applying unsupervised NLP methods to structured, moderate-scale interview data on psychedelic experiences; (2) to compare the experiential landscapes elicited by DMT-harmine and placebo-enhanced meditation through combination of numeric indicators from an NLP method and qualitative findings from manual thematic analysis; and (3) to explore the emergence of latent phenomenological themes overlooked by traditional theory-driven qualitative approaches and canonical metrics. In doing so, we aim to advance a richer, more integrative understanding of psychedelic phenomenology and its potential relevance for therapeutic applications.

## Methods

The research project at hand is part of a randomized controlled trial conducted in 2023. The initial study was approved by the Cantonal Ethics Committee of Zurich, Switzerland (BASEC-Nr. 2021 − 00180) and received an exemption from the Federal Office of Public Health (FOPH) for the administration of the controlled substance DMT. Written informed consent was obtained from all participants before active participation. The study was performed according to the Declaration of Helsinki (ClinicalTrials.gov identifier: NCT05780216 (https://clinicaltrials.gov/study/NCT05780216); Date of Registration: 03/21/2023).

### Study design

#### Participants

A total of 40 intermediate meditation practitioners were recruited to take part in a RCT designed as a three-day meditation retreat. Before enrollment, participants were first screened by telephone, followed by an in-person screening, including a medical check-up and psychological assessment. All participants were psychiatrically and medically healthy. For logistical reasons, two separate meditation retreats were held, with 21 and 19 participants, respectively.

#### Study procedures and setting

The main study was designed as a double-blind, placebo-controlled, between-subject trial. Forty healthy participants were randomly assigned to one of two groups (DMT-harmine or placebo) which were matched for gender. The two groups were similar across several baseline variables (Supplementary data I). Participants took part in one of two structurally identical 3-day meditation retreats held at the Zen meditation center Stiftung Felsentor in the Swiss Alps. Throughout the retreat, participants practiced sitting meditation in structured daily group sessions. The meditation practice was interleaved with walking meditation, mindful physical work, short breaks and meals.

The meditation retreat consisted of three phases: preparation (day 1), placebo or Drug administration (day 2), and integration (day 3). Each evening participants filled out structured questionnaires. On the second day of the retreat, participants received incremental doses of DMT-harmine or placebo while continuing their meditation practice. Study drugs were administered in a double-blind fashion. Participants were encouraged to adhere to the formal retreat structure only to the extent to which it remained comfortable to them and to take a gentle approach to meditation. Even more, the retreat schedule was adapted on that day, containing additional elements, such as guitar play and a gong ceremony, and relaxation periods after the last dose administration. In addition, participants acute subjective experience was assessed through psychometric scales (quantitative) eight times during that day: 90 min prior to the first dose administration, and 30, 60, 90, 120, 180, 240 and 360 min after the first dosing. Please find the original publication^49^ for further information on psychometric assessment and study design.

#### Substance and dosing

The pharmacological intervention utilized sublingual tablets containing DMT and harmine as described elsewhere^49,50^. Each tablet contained 30 mg of DMT and 30 mg of harmine. A total of four tablets was administered at 30-min intervals, resulting in a total dose of 120 mg of DMT and harmine. Placebo tablets were matched in taste and appearance using sucralose, menthol, and peppermint flavor for taste masking^49^. Both, the placebo tablets and the DMT-harmine sublingual tablets were prepared for double-blind administration by a certified pharmacist at the University of Zurich and administered in randomized order based on our previous studies with DMT and harmine in healthy human subjects (BASEC-Nr. 2018 − 01385). For more information on the pharmaceutical formulation of the study drug please find Egger et al.^50^.

#### Data collection

The qualitative data considered in the study at hand was collected using phenomenological interviews inspired by the MPI (Micro-Phenomenological Interview) method. MPI is an interview technique meant to obtain detailed chronological accounts of subjective experiences, while considerably reducing the influence of subjective biases otherwise associated with experiential reports^51^. MPIs follow a pre-defined iterative three-steps procedure. First, the interviewer guides the subject towards a single, temporally and spatially precisely situated particular experience. The evocation is considered successful when this particular experience becomes more vivid for the participant than the present situation^52,53^. Second, the interviewer facilitates acts of épochè – suspensions of conceptual superimpositions on lived experience – as he or she brings the subject back to that experience whenever their attention shifts away from it. Third, with each successive iteration, the interviewer gains progressively more detailed synchronic and diachronic descriptions of the same experience^52,53^.

Interviews were conducted after the second phase of the retreat. Neither the participants nor the interviewer or study team present at the study site were informed about the group allocations (double-blind). Due to time and staff constraints, we performed 28 interviews – 20 in the first and 8 in the second retreat. Intervieweeswere randomly selected from the participant pool. At the study site, interviews were conducted in a quiet and separate location to create a safe and pleasant atmosphere for the participant. Only 7 participants from retreat 1 were interviewed in-person, within 2 days of their experience. For logistical reasons, the residue of participants was interviewed online, no later than 11 days after their experience. All 8 participants of retreat 2 had their interview in-person and on the same day of their experience. All in all, we interviewed 16 people of the verum and 9 of the control condition. All interviews were audio recorded.

### Data preprocessing

All data preprocessing was performed in Python (version 3.9 and 3.11) using the JupyterLab environment (version 4.2.5) or Python-based software.

#### Interview audio recordings

All 28 interview recordings were manually quality-checked by DM. Due to technical issues with the recording device, 4 audio files were not usable and hence excluded for further analysis. Moreover, one participant from the verum group was interviewed twice because the first interview had to be cut short, resulting in 24 audios from 23 different people.

The remaining 24 audios were transcribed using version 0.3 of the open-access, python-based, AI-powered transcription software “noScribe”^54^. noScribe has been specifically developed for use in qualitative research and journalism and it runs completely local on the user’s computer. Transcripts were manually reviewed and cleaned by JTTS, and personal identifiers (e.g. names, date of birth, unrelated details of a person’s biography) were removed from the text. All transcripts were reviewed for a second time by two independent researchers (RG/DA) later in the text annotation process. The two transcripts from interviews with the same participant were merged into a single file resulting in 23 distinct interview transcripts. On average, transcripts counted 4238.8 words in the verum and 2714.3 words in the placebo group.

#### Text data for NLP model

For automated text annotation with BERTopic, interview transcripts had to be segmented into smaller analysis units. In line with the literature, we considered both participant’s and interviewer’s speaking parts for analysis^55^. Unspecific or isolated text segments, for example silence notations or another person’s comment within a person’s speaking part, were removed from the text corpus. Advanced transcript cleaning and segmentation was performed using spaCy’s German transformer pipeline “de_dep_news_trf”^56^. We opted for a sentence-based segmentation of the transcripts. The procedure yielded 6636 individual analysis units for all 24 interviews. For the individual experimental conditions, this led to 4609 and 2026 sentences for verum and placebo group respectively.

All python scripts used for cleaning and segmentation were written by JTTS (see Supplementary data II for code example).

### Data analysis

We considered 23 interview transcripts from 23 different participants for analysis. All analysis steps were performed in German language. Coding was done using Python (version 3.9 and 3.11) in the JupyterLab environment (version 4.2.5). The manual thematic analysis (text annotation, frequency-based analysis) was done with MAXQDA 24 Analytics Pro (version 24.6)^57^.

#### NLP text analysis

**Fig. 1:**
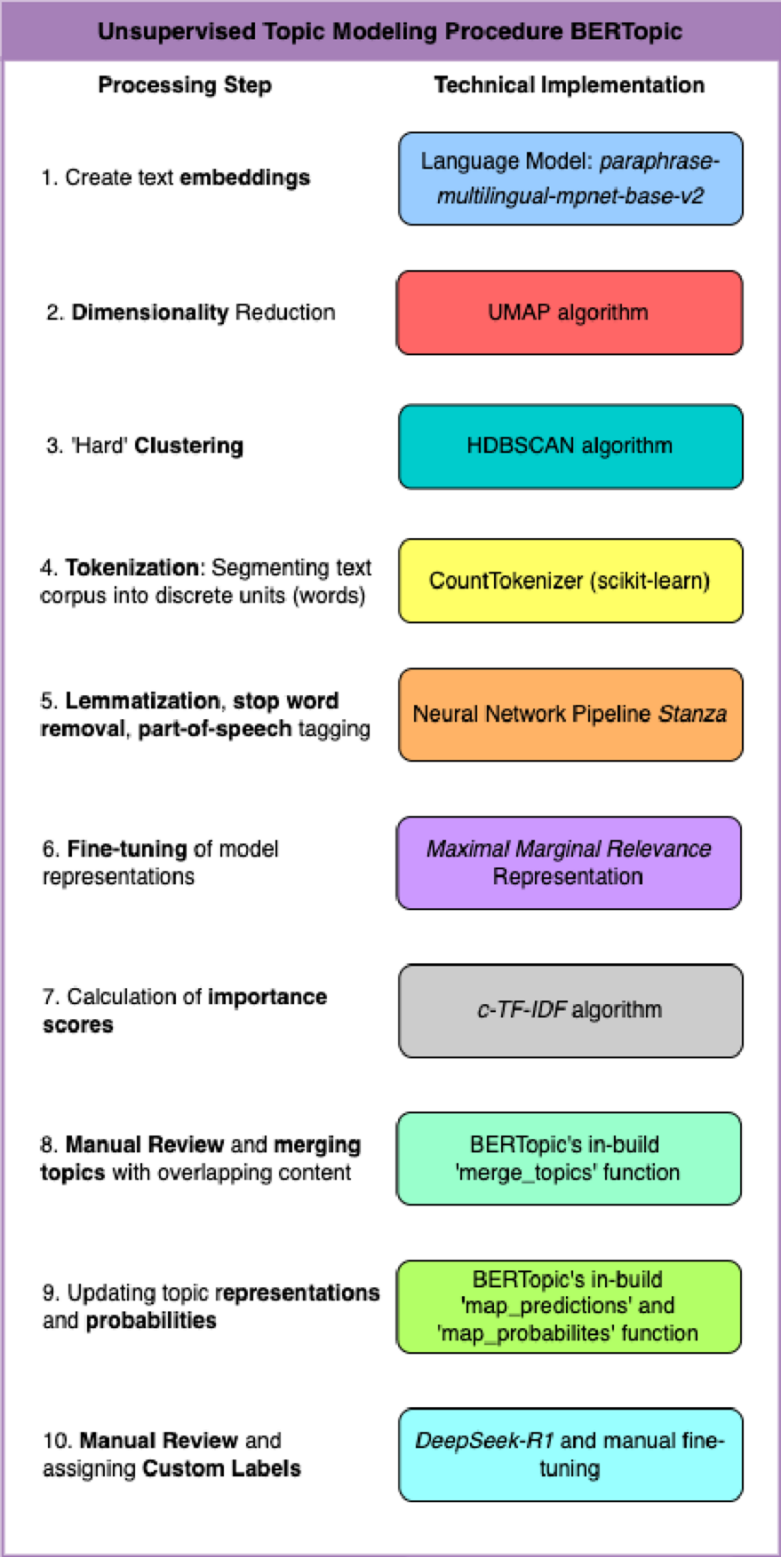
Steps of the unsupervised topic modeling procedure using the python library BERTopic. The figure outlines the 10 individual processing steps and their technical implementation as used for the study at hand. Detailed descriptions of the individual steps are provided in section ‘NLP Text Analysis’.

To explore subjective effects and common themes present during participants’ psychedelic or meditative experience, respectively, we utilized the state-of-the-art Python library BERTopic^58^. BERTopic leverages transformer-based language models in several steps along the modeling process and has a modular structure that by default comprises four main steps which are run in sequence: sentence-transformers, UMAP, HDBSCAN, and c-TF-IDF. Our topic modeling procedure is informed by the work of Haag and colleagues^41^ and was adapted to accommodate the unique characteristics of the dataset at hand. The individual steps of the procedure are outlined in Fig. 1 (see Supplementary data III for an implementation in python code).

We opted for an unsupervised topic modeling approach, which means that the topic model and its constituent sub-models were not trained on domain-specific data, resulting in a more explorative and unbiased (“unsupervised”) modeling procedure. First, we transformed the text data into numerical representations, resulting in so-called “embeddings”. This was done using the well-established multilingual sentence transformer model *paraphrase-multilingual-mpnet-base-v2*^59^. Next, we reduced the dimensionality of the data to save memory and computational resources^60,61^. The target dimensionality was defined based on the two quantitative metrics Nearest-Neighbor-Preservation (NNP)^62^ and Distance Preservation Error (DPE)^63^. Dimensionality reduction was done using the built-in *UMAP* algorithm^64^. Next, we clustered the data using BERTopic’s default *HDBSCAN* clustering algorithm^65^. Afterwards, we tokenized the data using the well-established *CountVectorizer* from Scikit-Learn library^66^. We combined Tokenization with Lemmatization; a process that converts a word to its root word (e.g., hearing to hear). Nouns and adjectives were found most informative for our research aim, hence we extracted all lemmatized nouns and adjectives from the text corpus using Part-of-Speech Tagging^67^. We also defined a list of stop words that would be neglected for the topic modeling procedure. The list of all stop words can be found in the python script in Supplementary data III. We performed all four steps using the python natural language analysis package Stanza^68^. Next, we let BERTopic assign importance scores to each unique word within the topic representations based on the statistical metric class-based term frequency-inverse document frequency^58^. Finally, we fine-tuned the model output using Maximal Marginal Relevance (MMR)^69^.

Topics with substantial thematic overlap were merged. For the sake of simplicity, all topics that did not sufficiently contribute to the understanding of participants’ subjective experience, because they exhibited an unusual degree of incoherence or unspecific mentions, were labeled as “noise” and manually merged into one single noise topic. Following Lezhnina^70^, an interdisciplinary team (JTTS, EV, MS) combined qualitative and quantitative metrics to assess model outputs (see Table 1). We tried to strike a balance between meaningfulness of a topic and appreciation of experiential diversity.

**Table 1:** Qualitative and quantitative metrics for automated topic modeling procedure. The four established metrics presented here were used as a standardized procedure for quality assessment of all BERTopic outputs.

Metric	Abbreviation	Type	Description
Topic coherence	TC	Quantitative (summary score)	Score between 0–1 that indicates the degree to which key words in a topic are semantically related
Topic diversity	TD	Quantitative (summary score)	Score between 0–1 that indicates the variety of topics by looking at uniqueness of key words across topics
Proportion of outliers	OUT	Quantitative	Indicates the share of analysis units that could not be assigned to one of the identified topics
Topic interpretability	TI	Qualitative (subjective)	Subjective assessment of the informative value and meaningfulness of a given topic

For the final outcomes, we inspected each topic individually and assigned a custom label which seemed to best capture the content of the assigned sentences. Labels were created using the Ollama-embedding of the large language reasoning model *DeepSeek-R1*^71^ and manually fine-tuned. We visualized the final topic representations using the Python WordCloud library^72^ and the built-in Intertopic Distance Map (IDM) functionality of BERTopic. An IDM is a simplified mapping of the total number of topics and visualizes the overlap or separation between all identified topics as well as each topic’s size in a 2-dimensional vector space.

The described procedure was first applied to the full text corpus comprising all 23 interview transcripts. Afterwards, we investigated the interview transcripts from the verum and the placebo group individually.

#### Manual interview coding

Two researchers (RG and DV) reviewed all 23 transcripts to prepare for manual text annotation. We utilized a hybrid coding approach, following a pre-defined coding system which was derived from established psychometric scales used in the main study and the team’s research-informed assumptions about psychedelic and meditative experiences. The initial code system comprised six main codes (see coding system in Supplementary data IV). Meanwhile, due to limited empirical evidence on psychedelic use in experienced meditators in the context of a meditation retreat, we allowed for the emergence of new codes during the coding process. We used sentence-based coding to avoid excessive loss of information and to maintain comparability with the results of the NLP analysis. We refined the code system iteratively, adding new codes as they arose from the data.

A team consisting of the two coders and at least one mediator (JTTS or EV) reflected on the coding process. Among other things, the team evaluated the intercoder reliability, using the built-in advanced analysis functionalities of MAXQDA Analytics Pro^57^. We carved out the codes and interviews, respectively, with the highest intercoder disagreement and had them recoded by both coders. This was repeated until we reached an overall intercoder agreement of about 90%, considering the codes used in each document. The manual coding of the interviews was performed independent from the results of the NLP analysis. The two researchers coding the interviews were not involved with the NLP analysis.

#### Manual quantitative analysis

Upon completion of the coding process, JTTS performed a frequency-based thematic analysis of the interview transcripts. We focused our analysis on the following seven main codes, and their associated sub-codes: *Psychometrics*, *Control*, *Sensations*, *Meaningfulness*, *Well-being/Life Satisfaction*, *Side effects* and *Valence*. In addition, we included the study’s setting factors, especially the code *Placebo or Substance*, in our analysis, which was informed by the bottom-up results of the NLP analysis. For each code and sub-code, we looked at total and co- appearances across both groups and performed a between-group comparison. As a last step, we compared the results from the manual to the outputs of the automated analysis. All findings and their possible implications were discussed in the research team.

## Results

### NLP analysis

Descriptive statistics for each analysis are presented as either frequencies (in integers) or proportions (in %). Topic descriptions, sample quotes and topic prevalence across analyses are provided in Supplementary data V. For visualizations of topic frequency distributions see Supplementary data VI.

#### Across-group findings

**Fig. 2:**
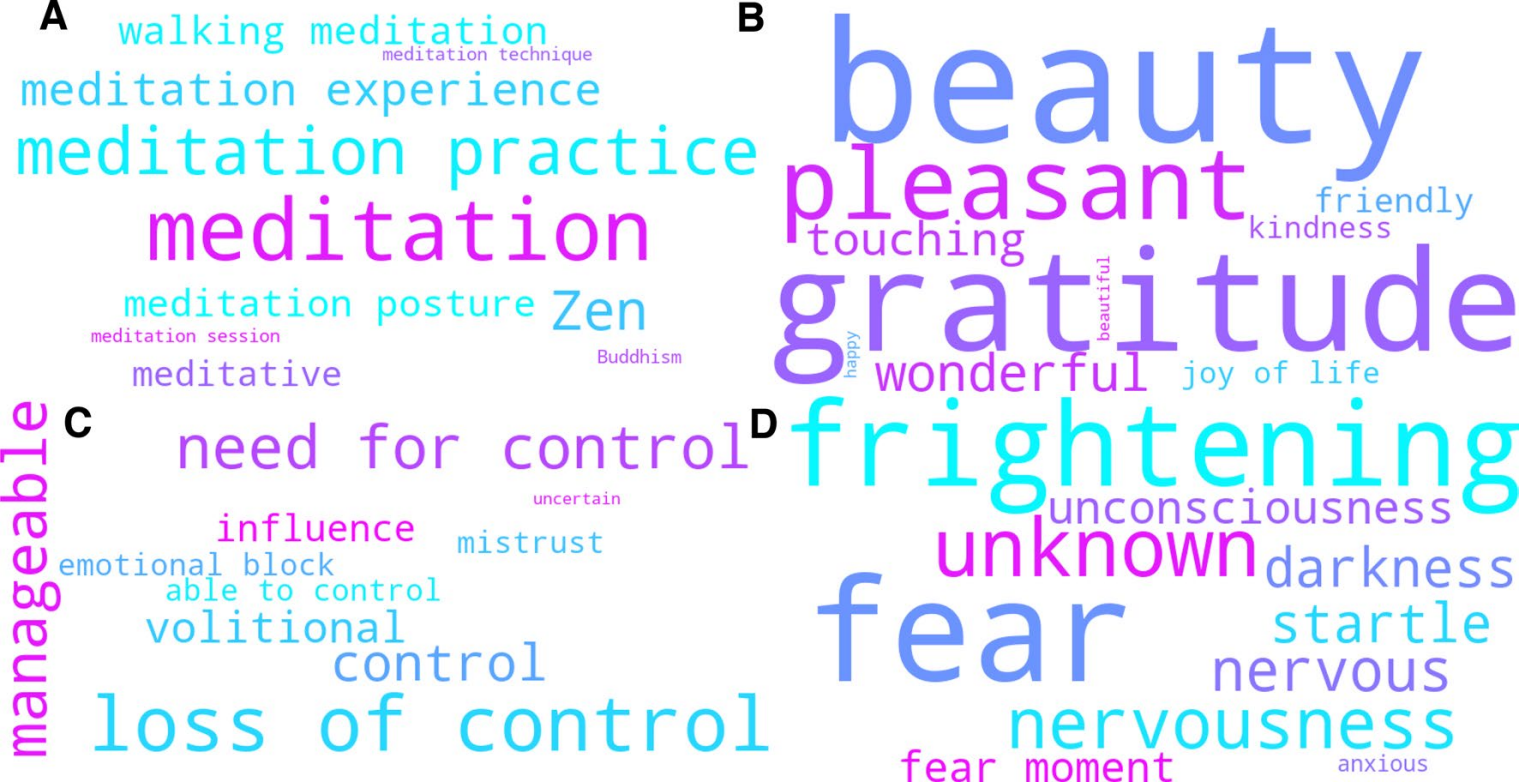
Word clouds across-group analysis. Visualization of the topic-defining key words for four topics representative of the diverse themes from the across-group analysis. **Top-Left**: ‘Contrasts and similarities between ordinary and enhanced meditation experience’; **Top-Right**: ‘Profound positive affect and uplifted emotional states’; **Bottom-Left**: ‘Control-acceptance dynamics; **Bottom-Right**: ‘Facets of fear’. In each word cloud, the size of the word corresponds to the relative importance of that given word for a given topic, as determined by the c-TD-IDF score. The color of a given word does not carry a deeper meaning.

The topic modeling analysis yielded 28 distinct topics plus one noise topic across all 23 interview transcripts. On average, participants referenced 23 topics during their interview. We identified 19 topics on acute subjective effects (ASE), four on reflective and integrative effects, and six topics addressed contextual factors. Topic ‘Interplay of subjective effects and contextual factors’ was assigned to both the cluster on ASEs and the cluster on contextual factors. Word cloud representations of topic-specific keywords from four representative topics are shown in Fig. 2. Additional visualizations and underlying c-TF-IDF scores for all 29 topics can be found in Supplementary data VII and VIII.

**Fig. 3:**
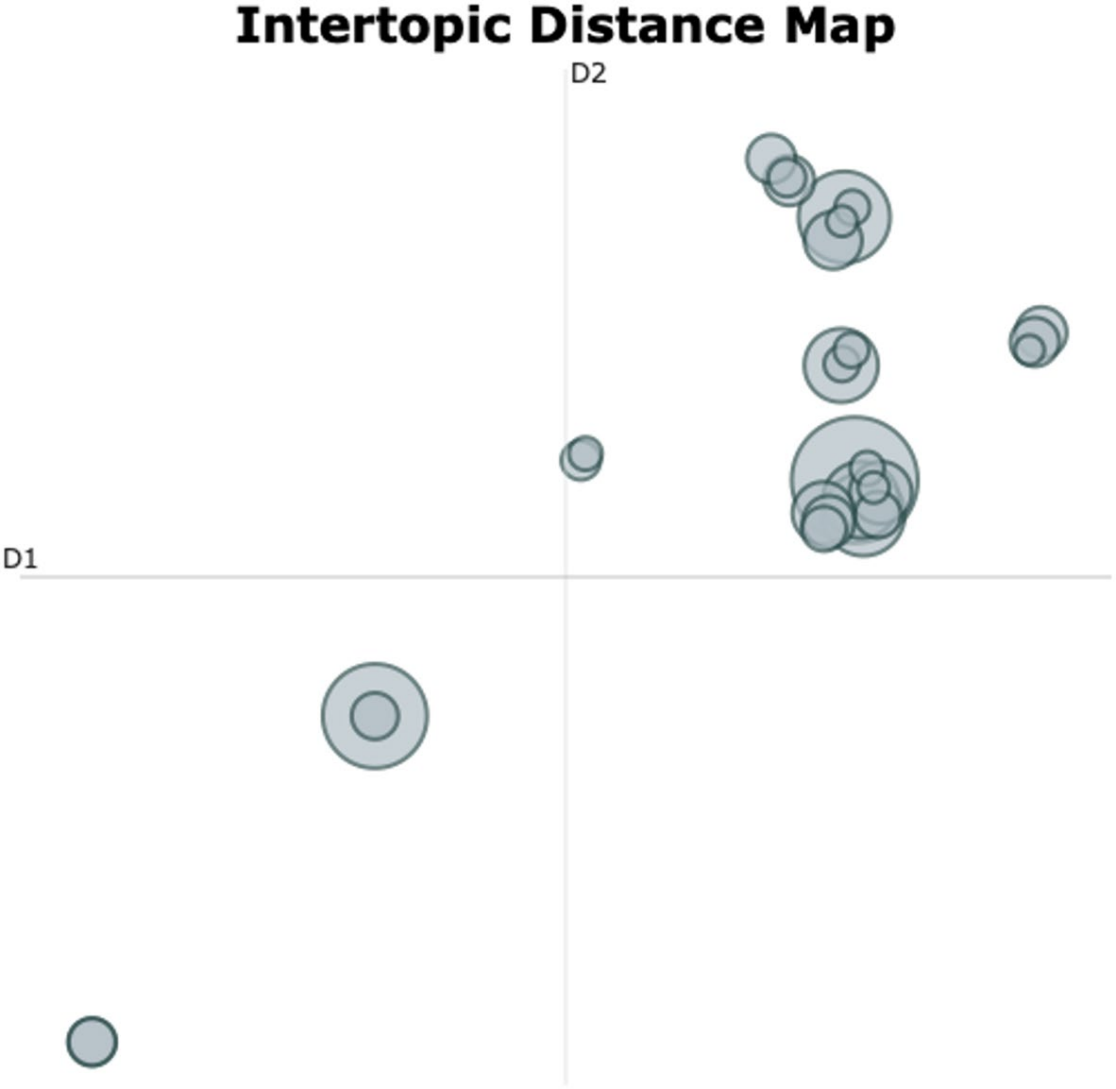
Intertopic Distance Map (IDM) across-group analysis: The figure shows the two-dimensional distances between the 29 distinct topics (including noise topic) of the across-group analysis. Each bubble represents one topic, where size of a bubble indicates importance (total count) and distance between two given bubbles indicates the semantic distance between these two topics. The location of a bubble in the map carries no specific meaning.

The across-group topic modeling procedure showed the highest quality, as indicated by the largest score on Topic Diversity (TD = 0.976) and an average Topic Coherence (TC = 0.53) while producing the lowest share of outlier sentences (37.8%). We found a complex semantic landscape with both shared and divergent themes emerging across participant groups. Some topics showed significant semantic overlap, as indicated by two vertically arranged dense clusters of topics close to each other in the upper-right corner of the Intertopic Distance Map (Fig. 3). This was supported by our subjective topic interpretability analysis which showed that a great share of topics had formed around subjective effects (82.1%). Meanwhile, the map showed four main isles of topics – two in the upper-right and two in the lower-left corner - positioned further apart from the clusters, indicating that the procedure discovered semantically distinct and group-specific themes. This was also reflected in the large TD score.

#### Between-group findings—verum

**Fig. 4:**
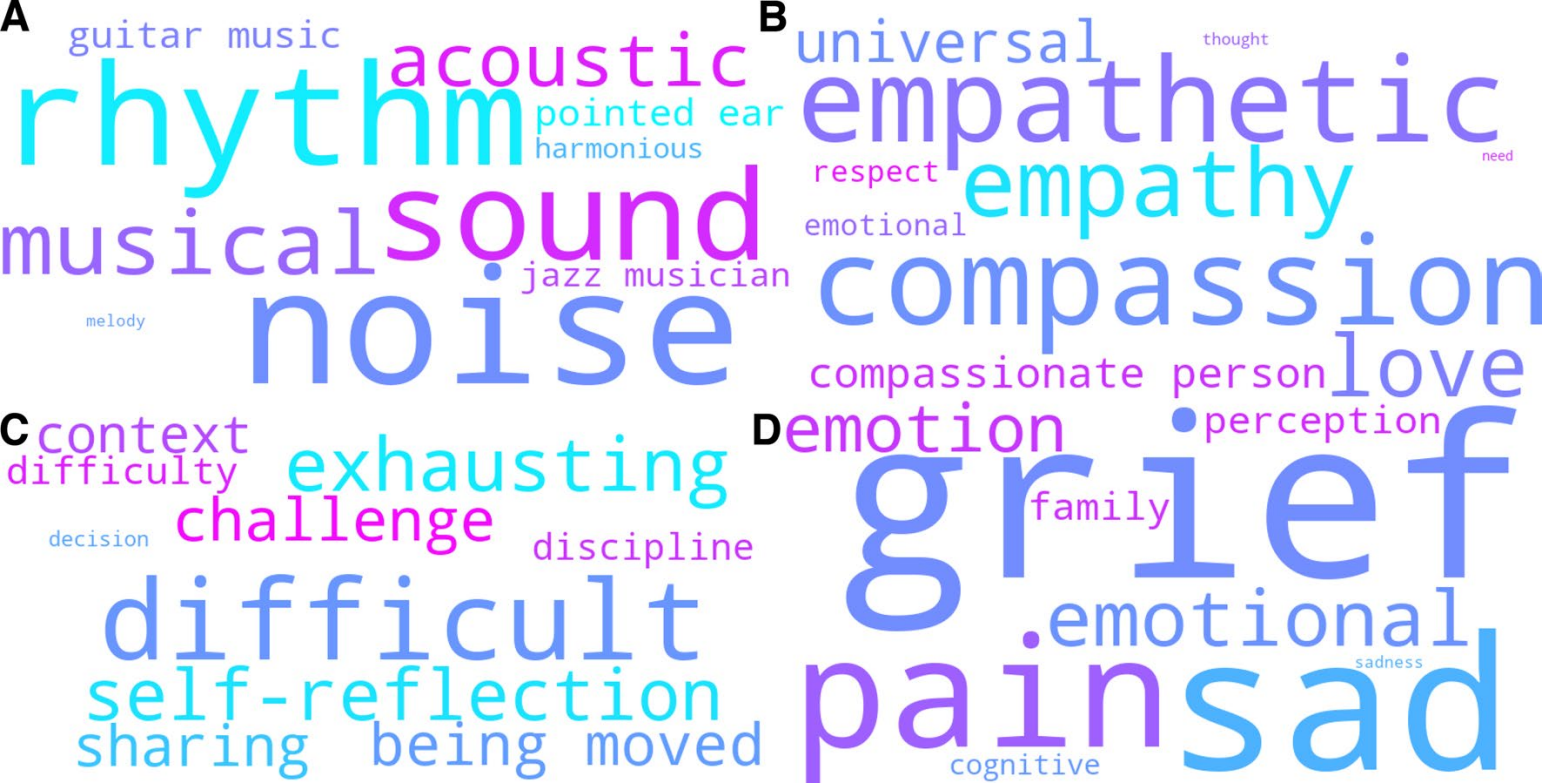
Word clouds DMT-harmine condition. Visualization of the topic-defining key words for four topics representative of the diverse spectrum of subjective effects found in reports from the verum group. **Top-Left**: Transformative power of music’; **Top-Right**: ‘Empathy, compassion, metta’; **Bottom-Left**: ‘Challenging experiences’; **Bottom-Right**: ‘Open emotionality and emotional integration. In each word cloud, the size of the word corresponds to the relative importance of a given word for a given topic, as determined by the c-TD-IDF score. The color of a given word does not carry a deeper meaning.

The topic modeling procedure resulted in 24 distinct topics plus one noise topic across the 14 interview transcripts from the verum group. We identified 18 ASEs, four reflective and integrative effects, and only two topics addressed contextual factors. Participants in the verum group touched on 20 topics during the interview on average. Figure 4 visualizes the key words of four representative topics from the verum condition in the form of word clouds. Residual word clouds are provided in Supplementary data IX. C-TF-IDF scores can be found in Supplementary data X.

**Fig. 5:**
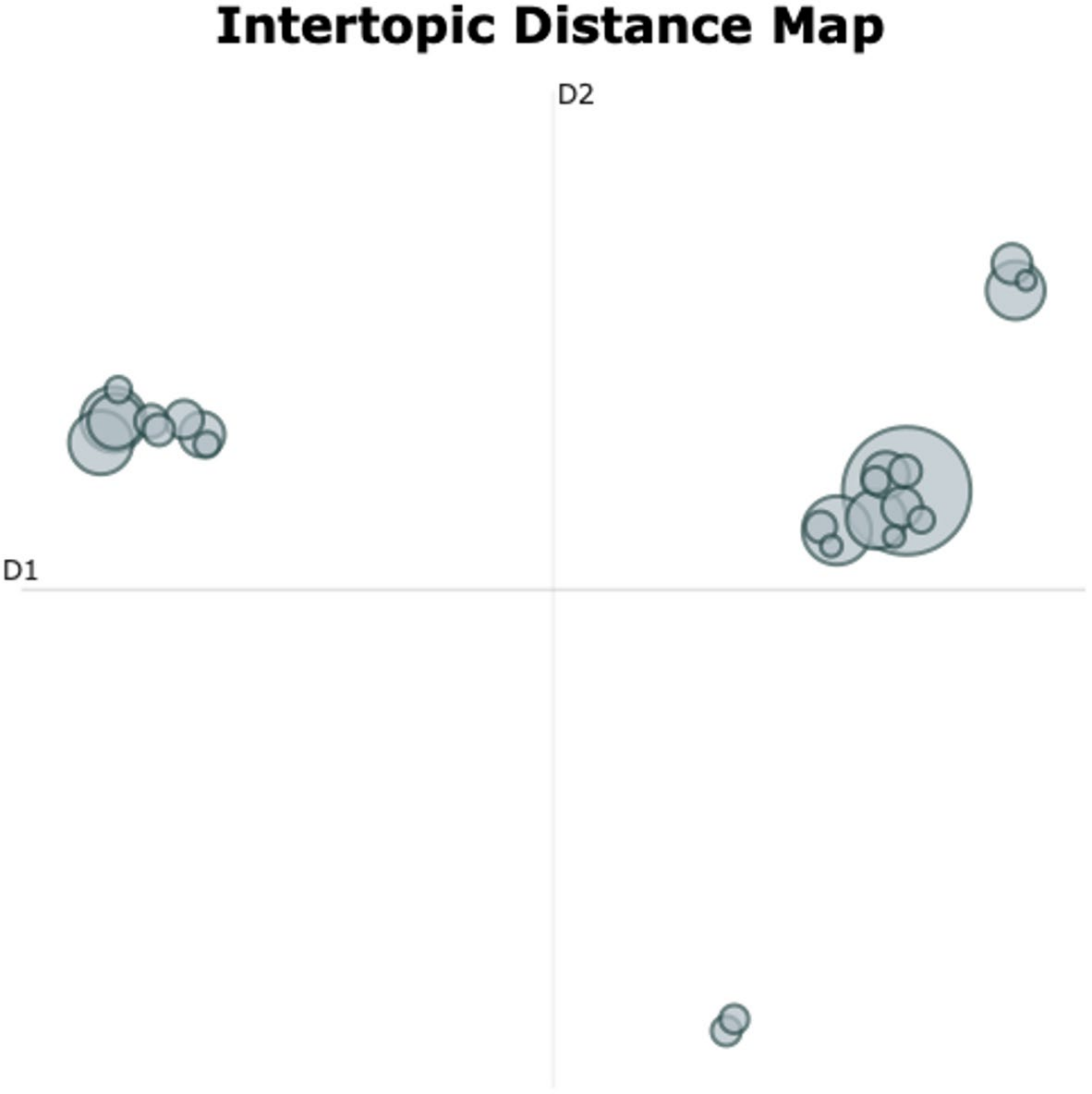
Intertopic Distance Map (IDM) verum group analysis. The figure shows the two-dimensional distances between the 25 distinct topics (including noise topic) of the verum group analysis. Each bubble represents one topic, where size of a bubble indicates importance (total count) and distance between two given bubbles indicates the semantic distance between these two topics. The location of a bubble in the map carries no specific meaning.

We found 20 shared topics between the outputs of the verum group and across-group analysis, indicating a significant thematic overlap. There were also clear parallels in the semantic topographies of the two outputs. Both showed two main thematic hubs addressing the prominent theme of subjective effects. Group-specific (across-group analysis) or sub-themes of the dominant topic (verum-only) were covered by a few moderately prevalent outlier topics. Comparison of the Intertopic Distance Maps (IDM) reinforced this finding (see Figs. 3 and 5). In both cases we found small distances within-hubs and greater separation from outlier topics. Clustering for verum group-only showed lower heterogeneity compared to the across-group analysis, as indicated by a lower topic diversity score (TD = 0.971) and more tightly clustered topics in the IDM. This was also reflected in a weaker topic interpretability (TI). Topics showed greater within-topic coherence, as indicated by a score of TC = 0.547. The most frequent topic ‘Acute phenomenology: Dynamic self–perception shifts and state transitions’ was an exception here, as it stood out with a disproportionately high count of 740 assigned sentences. The corpus of assigned sentences and the topic’s key word representation exhibited high incoherence.

#### Between-group findings—placebo

**Fig. 6:**
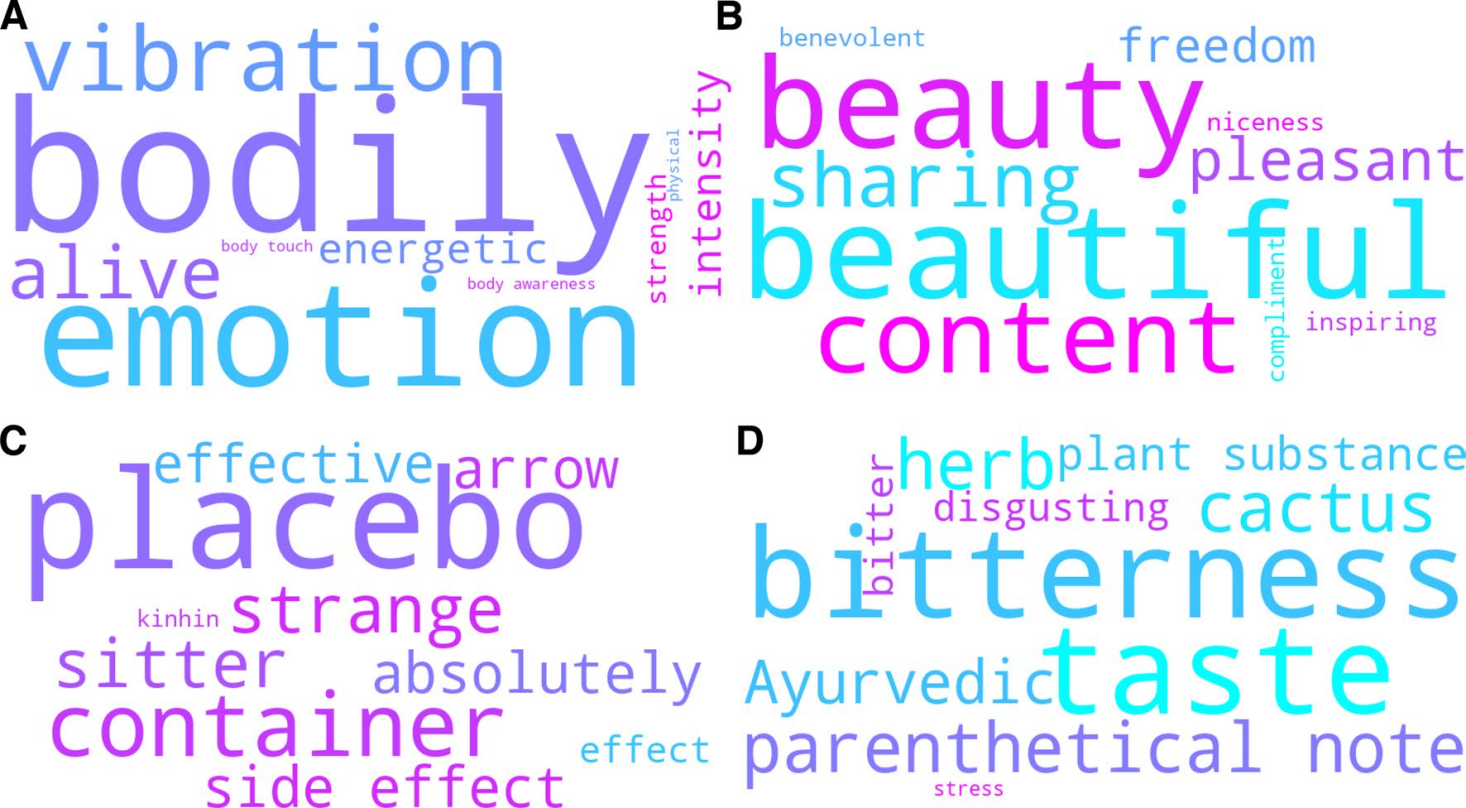
Word clouds placebo condition. Visualization of the topic-defining key words for the top-four topics from the placebo group analysis. **Top-Left**: ‘Energetic experiences and physiological sensations’; **Top-Right**: ‘Profound positive affect and uplifted interpersonal-contextual emotional states’; **Bottom-Left**: ‘Placebo effect; **Bottom-Right**: ‘Temporal dynamics and dose-dependent effects study medication’. In each word cloud, the size of the word corresponds to the relative importance of a given word for a given topic, as determined by the c-TD-IDF score. The color of a given word does not carry a deeper meaning.

The topic modeling analysis on the 9 interviews from the placebo group resulted in 11 distinct topics and one noise topic. We classified 7 acute and one reflective and integrative effect(s). Five topics addressed contextual factors. Topics ‘Interplay of subjective effects and contextual factors’ and ‘Profound positive affect and uplifted interpersonal-contextual emotional states’ were assigned to the cluster on contextual factors and the cluster on acute subjective effects. Figure 6 presents word cloud representations of four topics that are representative of the experiential content reported by the placebo group. Residual word clouds and underlying c-TF-IDF scores are provided in the Supplement (XI and XII). On average, participants in this group referenced 15 different topics in their interviews.

**Fig. 7:**
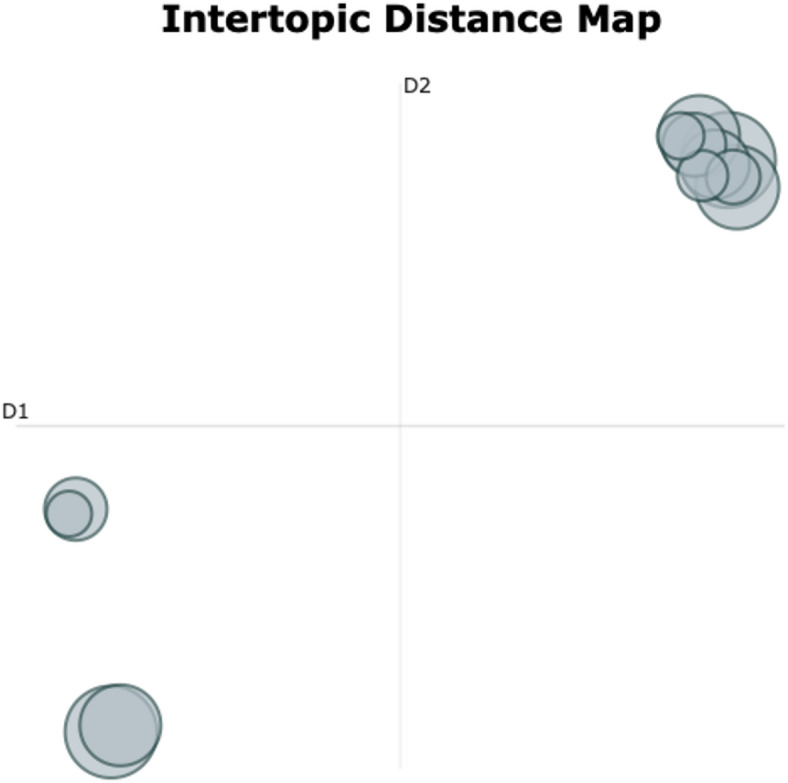
Intertopic Distance Map (IDM) placebo group analysis. The figure shows the two-dimensional distances between the 12 distinct topics (including noise topic) of the placebo group analysis. Each bubble represents one topic, where size of a bubble indicates importance (total count) and distance between two given bubbles indicates the semantic distance between these two topics. The location of a bubble in the map carries no specific meaning.

We found the largest thematic diversity across all three outputs, reflected in a topic diversity score of TD = 0.983. This was contrasted by the lowest topic coherence (TC = 0.532) and the largest share of outlier sentences (45.9%). The lowest TC score suggested the highest within-topic heterogeneity across all three analyses. The great share of outliers indicated that a great portion of the data did not cluster well within the modeled topic space. Topics in the IDM aggregated around three dense and far apart distinct topic hubs with no salient outliers (Fig. 7). This suggested strong semantic overlap between certain topics, strong semantic diversity between hubs of similar topics and little to no reports of extreme altered-state phenomena in individual interviews of the placebo group. Similar to the highest ranking topic from the verum-only analysis, the most frequent topic ‘Acute phenomenology: Somatic overload, emotional release, and regulation’ was concerned with a broad spectrum of acute altered-state effects, however strongly weighted toward somatic-affective load and regulation. Topics on acute subjective effects made up the lowest share (63.6%) across all three model outputs, while topics on contextual factors accounted for the maximum share of 45.5% compared to 8.3% (verum) and 21.4% (across).

#### Latent topics

Our automated analysis surfaced several underreported yet theoretically intriguing themes representing conceptual spaces often overlooked in mainstream scientific discourse on psychedelic experiences and their therapeutic potential. The most prominent of these topics, which we refer to here as *latent topics*, was ‘Contrasts and similarities between ordinary and enhanced meditation experience’ which ranked among the top four most frequent topics in each of the analyses. Total counts reached 182 (verum), 453 (across) and 107 (placebo). Another latent topic ‘Placebo effect’ stood out because it appeared in the placebo-only but not the verum-only analysis. An overview of all latent topics and their prevalence is provided in Table 2.

**Table 2:** Latent topics automated topic modeling procedure. The table shows the custom label assigned to a given latent topic and its prevalence across the outputs of all three NLP Analyses. In brackets: total number of coding units assigned to a given latent topic for a given form of analysis.

Latent Topic	Form of analysis		
	Across group	Between group—Verum	Between group—Placebo
Control-acceptance dynamics	**X** (161)	**X** (30)	
Placebo effect	**X** (83)		**X** (64)
Equanimity and authentic presence	**X** (137)		**X** (78)
Interactions between meditation and psychedelics	**X** (90)	**X** (182)	
Relational and social dimensions of the experience	**X** (50)	**X** (46)	
Transitions, fluctuations, and impermanence of experiential states	**X** (226)	**X** (66)	**X** (48)
Contrasts and similarities between ordinary and enhanced meditation experience	**X** (453)	**X** (191)	**X** (107)
Profound positive affect and uplifted interpersonal- contextual emotional states			**X** (114)
Personal meaning and embodied insight	**X** (96)		
Interpersonal attunement with trip sitter	**X** (39)		
Simplicity		**X** (24)	

### Manual quantitative analysis

#### Descriptive analysis—codes

All 23 interview transcripts were included in the manual analysis. The most frequently used top-level codes were ‘Control’, ‘Mental Awareness’ and ‘Psychometrics’, with global counts of 346, 312 and 164. Four new top-level codes arose from the inductive coding of the text data. Data-driven subcodes were iteratively added to the code system, resulting in a total of 39 (sub)codes. We refer to Supplementary data IV for an overview of the final code system, a description of, and sample text data for each of the codes.

#### Descriptive analysis—discrepancy verum vs. placebo

The manual analysis yielded almost three times more coded sentences in the verum than in the placebo group – a respective total of 886 and 320 sentences. The bias was especially pronounced among codes representative of subjective effects; (a) the four ‘Psychometrics’ sub-codes and (b) sub-codes of the top-level code ‘Sensations’. Table 3 shows absolute counts for all 9 sub-codes and illustrates the discrepancies in code appearances between the two experimental conditions.

**Table 3:** Between-group comparison of main code ‘Psychometry’ and main code ‘Sensations’ from manual thematic analysis. The table shows the group-wise and the total counts for sub-codes of the two primary deductive codes ‘Psychometry’ and ‘Sensations’ used to code for (acute) subjective effects in participants’ experience reports.

Code	Sub Code	Verum	Placebo	Total
Psychometrics	NDA	21	5	26
	ME	58	9	67
	PI	57	2	59
	EB	10	2	12
Sensations		-	-	-
	Auditory	27	10	37
	Visual	35	3	38
	Mental awareness: Cognition	189	39	228
	Mental awareness: Emotions	64	20	84
	Bodily awareness	64	38	102
Total:		530	130	660

**Fig. 8:**
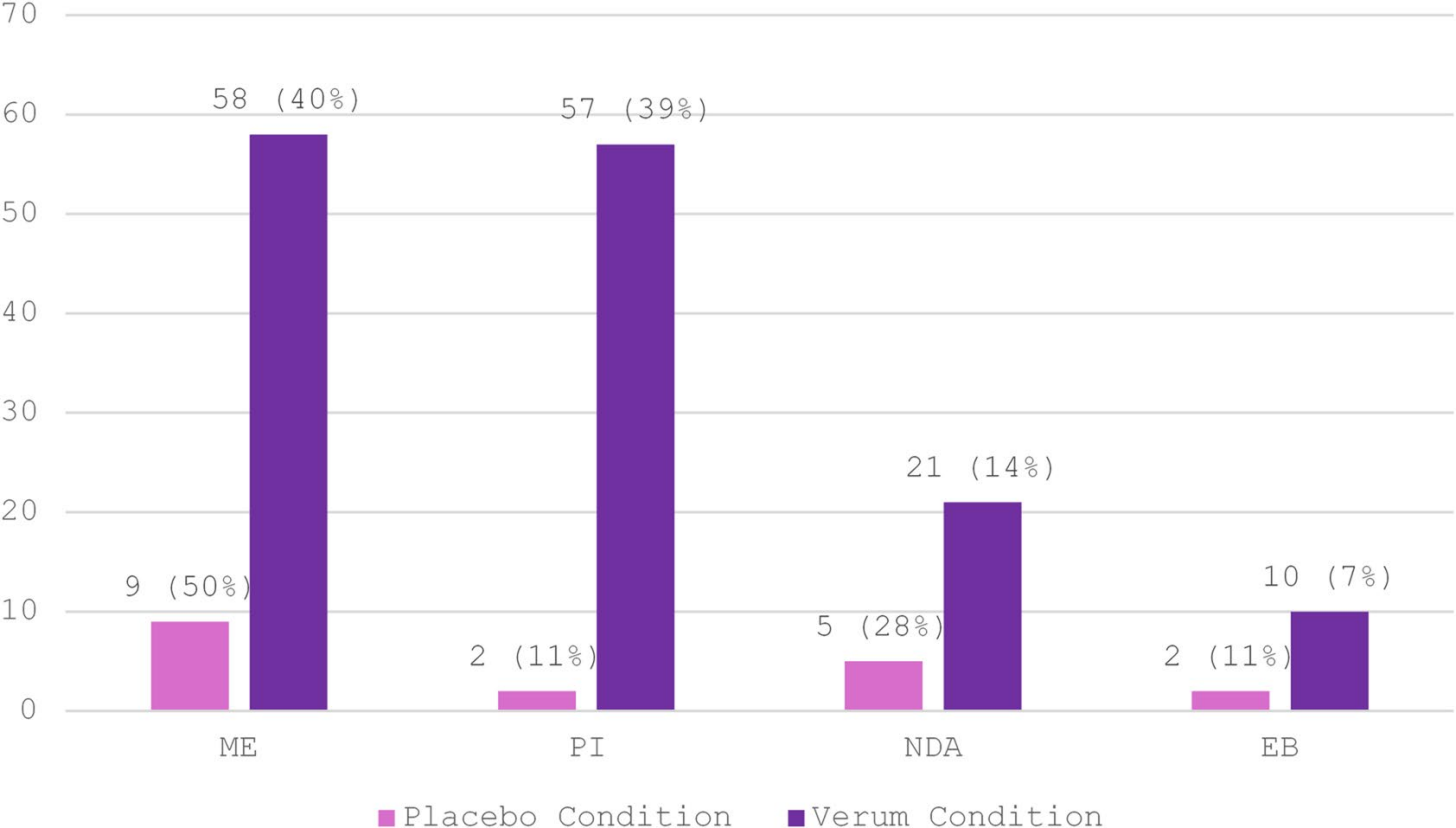
Total appearances of Psychometrics sub-codes across experimental conditions. Bars show the absolute counts for each sub-code (ME, PI, NDA, and EB) across all 15 interviews in the verum and all 9 interviews in the placebo condition respectively (see Table 3). In brackets: Percentage contribution of each sub-code to the total number of Psychometrics codes within the placebo and verum condition. Percentages are normalized within condition.

Code ME dominated the Psychometrics sub-codes with appearances in 70% of all interviews. This was followed by PI (62.5%), NDA (58%) and EB (50%). Mystical-type experiences (ME + NDA) were the most prevalent sub-codes in reports from both groups but were represented in greater proportion in the placebo group. Insight experiences (PI) had almost equal importance in verum reports. Figure 8 visualizes the absolute counts and relative frequency-based importances for sub-codes of the top-level code ‘Psychometrics’ across the two experimental conditions.

#### Mental and bodily awareness

Awareness of mind and body (‘Mindfulness’) saliently stood out in reports from both groups (Table 3). Code Mental Awareness appeared in 96% of all interviews. The sense of increased mindfulness seemed heavily driven by an awareness of mental processes in the verum group, indicated by greater counts for sub-codes ‘Visual’ and ‘Auditory’. Sub-code ‘Emotion’ was present in 85% of the interviews but 76.2% of all codes fell into the verum group. For ‘Cognition’, the effect was even more pronounced with a ratio of 39 / 189 counts (placebo / verum). Mental Awareness was followed by Bodily Awareness (92% of all interviews) with almost twice as many codes in the verum group.

#### Altered sensory perception

**Fig. 9:**
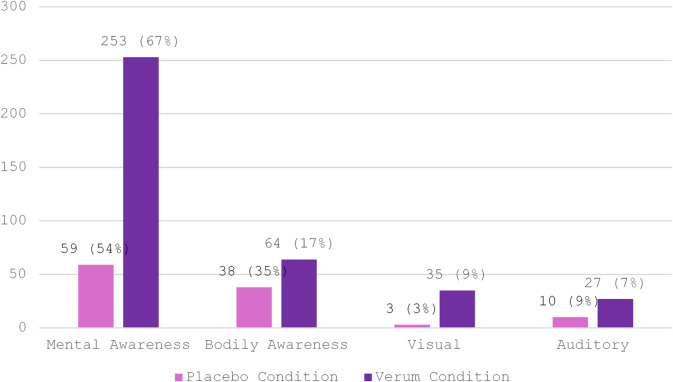
Total appearances of Sensations sub-codes across experimental conditions. Bars show the absolute counts for each sub-code (Mental Awareness, Bodily Awareness, Visual, and Auditory) across all 15 interviews in the verum and all 9 interviews in the placebo condition respectively (see Table 3). In brackets: Percentage contribution of each sub-code to the total number of Sensations codes within the placebo and verum condition. Percentages are normalized within condition.

Across all interview transcripts, 71% contained code Auditory and 54% code Visual. The total count for ‘Auditory’ was more than twice as much in the verum compared to the placebo condition (Table 3). Auditory effects ranged from a “perfect’ or “pure” hearing experience to experiences of synaesthesia (e.g., seeing sounds or colors changing with the breath) and auditory pareidolia (imposing meaningful patterns on random sounds or noise). Similarly, visual effects seemed starkly more present for the verum group. Only 3 out of 38 codes fell in the placebo group (Table 3). The spectrum of experienced effects ranged from high sensitivity to light, visual synaesthesia and (pseudo-) hallucinations, to complex visual imagery with open and closed eyes. Figure 9 visualizes the absolute counts and proportional distributions (relative importance) for sub-codes of the main code ‘Sensations’ across the two experimental conditions.

#### Emotional valence and affect

We coded for positive and negative emotional valence in the manual analysis. The results showed 78 codes across 21 interviews for positive and 40 codes across 11 interviews for negative emotional valence. Negative valance was balanced between the two groups; positive valence showed a slight group-bias towards the verum group. Verum participants mainly referred to their subjective experience when expressing both positive and negative affect (“*It wasn’t fear*,* but more like anxiety and darkness—yes*,* I would call it vermin. Crawling creatures rising up from the depths. And that was the moment when I thought*,* oh*,* I’d really like to get out now.*” – participant 36; “*Emotionally*,* throughout the whole experience*,* I was mostly filled with rapture and amazement because it was just so incredibly beautiful.”* – participant 34*).* Participants from the placebo group reported more negative and unpleasant side-effects from the study drug (“*Then I got this pill and it just tasted so disgusting to me. Somehow so artificial*.” – participant 04; “*I had stomach pains in between.*” – participant 17) and expressed positive affect towards contextual factors, often leaving subjective effects more in the background (“*And I really enjoyed the way you guided us through it.*” – participant 27; “*It was also quite impressive for you to be in the room with other people who are also going on a journey*.” – participant 01).

#### Latent topics

The inductive part of the analysis resulted in four latent main codes comprising a total of 15 sub-codes (see Supplementary data IV). The most prominent of the topics was ‘Control’. The code was present in 92% of the interviews, although 213 of the total 346 codes fell in the verum group. The sub-code “Placebo or Substance” was used 21 times, distributed across 13 interviews – 8 placebo and 5 verum. Contextual factors – main code ‘Setting’ and its seven sub-codes - were referenced in both experimental groups to roughly equal shares with a total of 237 coded segments.

## Discussion

This study presents the first application of Natural Language Processing (NLP) within a mixed-method framework to analyze a set of structured phenomenological interviews from an ecologically valid randomized controlled trial (RCT) involving psychedelic-augmented meditation. By combining automated topic modeling with manual thematic analysis, we systematically explored the subjective experiences of participants who received either a DMT-harmine formulation or placebo during a meditation retreat. This approach allowed us to examine both group-level experiential patterns and underrecognized phenomenological themes, contributing a novel methodological and conceptual lens to the growing field of psychedelic research.

We found that the states induced by meditation under DMT-harmine and meditation under placebo shared acute subjective effects and that participants from both conditions used similar vocabulary to describe their experiences. Meditators in both groups invoked Buddhist concepts and spiritual philosophy. The results highlighted an experiential prevalence of extra-pharmacological factors and their influence on the subjective experience under placebo and a dominance of acute subjective effects under DMT-harmine.

Our findings support the growing promise of data-driven, multi-method approaches for investigating psychedelic subjective effects. In particular, we demonstrate the utility of NLP techniques to complement qualitative analysis methods. Despite the unsupervised nature of our topic modeling approach and the relatively small data set, the topic models effectively captured both the diversity and nuance of participants’ subjective experiences when compared to the independent manual analysis. The analyses identified not only well-established acute subjective effects of psychedelics^2,4^, and similar states induced by meditation^9,10^ but context-specific and extra-pharmacological topics tied to the retreat setting. These results highlight the unique sensitivity of NLP methods to aspects of experience that often fall outside traditional theory-driven frameworks. Similar advances were recently reported by Bzdok et al., who used large language models (LLMs) to distinguish the experiential profiles of 30 psychoactive substances based on user reports^19^. Relatedly, Noah and colleagues used an embedding model from OpenAI to uncover compound-specific variations in reported visual effects of psychedelics^27^. Our results extend these insights to the setting of a controlled RCT and underscore the applicability of NLP even in smaller, structured datasets.

Several topics including the high-ranked topics ‘Acute phenomenology: Dynamic self–perception shifts and state transitions’ (verum), ‘Acute phenomenology: Somatic overload, emotional release, and regulation’ (placebo) and ‘Contrasts and similarities between ordinary and enhanced meditation experience’ (across all three analyses), exhibited notable thematic heterogeneity. This was reflected by incoherent key word representations of these topics, a reduced within-topic coherence and a smaller between-topic diversity. Such outcomes are characteristic of unsupervised topic modeling approaches, which, while powerful for exploratory analyses, may produce less targeted outputs without domain-specific guidance. As a result, some topics were less interpretable, with diminished thematic clarity. The effect was least pronounced in the across-group analysis. Based on these findings, we recommend caution when applying NLP techniques, such as BERTopic, to small-sized data sets. In line with previous findings, we found that the effectiveness of NLP techniques strongly depends on both the *quality* and the *size* of the data sample^73–75^.

Second, our results highlight the value of combining a data- and a theory-driven analysis approach. While traditional qualitative methods are prone to subjectivity bias^76,77^, this risk was mitigated by triangulating a manual coding process with NLP-based topic modeling. Both approaches independently revealed a strong group-effect for topic prevalence, particularly regarding code (topic) frequency and diversity of acute subjective effects. This was further supported by the high degree of thematic overlap between outputs of the automated across-group and the verum-group analysis. The convergence suggests that the observed group differences were not merely an artifact of investigator bias but likely reflect genuine experiential divergence between the two conditions. At the same time, the manual procedure enriched and corroborated the exploratory NLP analyses, including the prominence of spiritual jargon in both experimental groups and of contextual factors and their contributions to participants’ subjective experiences in the placebo condition. These results illustrate the complementary strengths of manual and automated methods in capturing the complexity of psychedelic experiences.

Our mixed-method approach uncovered a set of less recognized yet theoretically compelling themes. These *latent topics*, including themes like control, equanimity, impermanence or embodied wisdom, have largely remained underrepresented in mainstream psychedelic literature but gained increased attention in recent studies exploring the convergence of psychedelics and mindfulness practices^10,11,14,15,78^. We use the term *latent* not merely in the statistical sense, but to highlight how these themes have remained hidden in plain sight: present in anecdotal data yet rarely foregrounded in empirical research. Their emergence in our data reflects the capacity of automated topic modeling to detect subtle, patterned regularities in language that traditional hypothesis-driven approaches alone may overlook. These findings broaden the interpretive scope of psychedelic science and invite further inquiry into underexplored dimensions of psychedelic experiences.

A notable finding from the across-group NLP analysis was the high semantic similarity between reports from the placebo and the verum group, indicating that participants drew on similar vocabulary to describe their experience. This aligns with previous research highlighting similarities between psychedelic and meditative phenomenology^9,10^ and pointing toward potential convergence between these modalities^11,79^. Even more, the acute and post-acute subjective effects reported by placebo participants seemed to mirror those of the verum group, corresponding to well-established features of psychedelic experiences^2,4^. In support of our findings, Dikovskaya et al.’s natural language analysis had revealed that reports from floating tank and meditation experiences most closely resembled those from 5-MeO-DMT, N, N-DMT, and ketamine sessions, Together, these results suggest that altered states of consciousness - whether induced pharmacologically or through non-pharmacological means - may produce subjective effects that are generalizable across ASCs rather than being limited to drug-specific artifacts^45^.

Our quantitative investigation pointed towards a semantically homogenous conceptual framework for reports from both groups. Manual analysis revealed that many participants used Buddhist philosophy or spiritual concepts to describe and integrate their experiences; a pattern mirrored in NLP-derived topics such as ‘Personal meaning and embodied insight’, ‘Transitions, fluctuations, and impermanence of experiential states’ or ‘Interactions between meditation and psychedelics’. Some participants stated how their experience helped them to truly understand and embody the teachings from their meditation tradition (“*Two days later*,* I remembered the Heart Sutra and unpacked it. I really understood half of it*.” – participant 09; “*And when I first experienced its effects*,* it was clear to me that this was the path to the heavens*,* but not where Buddha wanted to get to the root of suffering.*” – participant 05). Others used their tradition’s jargon to express experiential differences (“*Um*,* and I feel like my psychedelic experience wasn’t about somehow deconstructing the experience*,* it wasn’t about seeing in every sound*,* in every*,* in every sensory perception*,* the impermanence and how it ends and re-emerges and is not me*.” – participant 25; “*So during normal meditation*,* I don’t usually experience these strong energetic phenomena that I just demonstrated during the session*.” ), or how the two modalities seem to intersect, support or even interfere with one another (“*And I feel that the meditative state*,* depending on the type of meditation*,* is probably the important thing*,* as it can be both beneficial and keep you firmly in the here and now.*” – participant 22; “*That it may take several units to achieve certain effects that may be possible more quickly with psychedelics. This means that psychedelics could speed up processes that take longer with meditation.*” – participant 17). The finding is supported by previous research emphasizing how language under psychedelics serves as *‘a window into the psychedelic mind*’^80^, and how spiritual or philosophical frameworks can serve as conceptual containers for meaning-making and integration of psychedelic experiences^81–85^.

It has been repeatedly pointed out that the phenomenology of psychedelic experiences is profoundly shaped by cultural and religious beliefs and that individuals often connect their subjective effects to culturally specific or spiritual and religious interpretations^86,87^. Given our RCT design and the participants’ extensive meditation background in various Buddhist traditions, a contemplative lens seems to have naturally shaped their narratives regardless of group assignment. However, we should assume to find different themes when conducting the same study in a population with different religious backgrounds (e.g. no contemplative background at all or trained in other traditions, for example shamanic practices) or embedding it in a different cultural framework (e.g. performing the study in an African or South American country). Nonetheless, spiritual and philosophical language may have offered participants a linguistic bridge to articulate ineffable or non-ordinary states that otherwise lay beyond linguistic habits; awareness of such meaning-making processes may therefore be relevant in therapeutic settings^88^.

Despite strong similarities, we found notable experiential divergence between the two groups. In the verum group, 75% of topics from the automated analysis pertained to ASEs compared to 63.4% in the placebo group. Reported acute effects were often intense and exhibited great diversity, including perceptual, emotional, cognitive and often self-related domains. In contrast, placebo participants’ subjective experiences showed less heterogeneity, effects were largely body-centric, and participants’ reports focused on coping and containment rather than allowing and experiencing the effects. Moreover, two of the seven topics from the placebo-only analysis contained a substantial number of references to contextual factors. This imbalance was mirrored by differences in thematic landscapes: while placebo participants distributed attention across a broader range of distinct topics, verum participants’ narratives converged on fewer, more dominant and related experiential themes. We interpret this as a kind of *focusing effect*, in which the vivid, intense, and sometimes overwhelming nature of the DMT-harmine experience foregrounded acute subjective effects in participants’ awareness and language. This led to high thematic diversity within a relatively narrow conceptual domain. In contrast, placebo-group participants reported effects that were often familiar, accessible and modulated by their meditation experience, seldom entirely novel (“*So all the elements that I know anyway*,* equanimity*,* focus*,* concentration*,* um*,* it also was much easier for me to meditate*” – participant 27; *“[…] That is the energetic phenomenon*,* which you are also familiar with*,* but to a lesser degree*,* from [your] meditation practice*“ – participant 16).

82.5% of all participants guessed their group correctly when asked in a survey after their study experience at the end of retreat day 2^49^. Nonetheless, both the manual and the automated analysis highlighted a mental occupation with the study drug among participants in the placebo group (code ‘Placebo or Substance’ and topics ‘Placebo effect’ and ‘Temporal dynamics and dose-dependent effects study medication’ respectively) wondering whether their subjective effects were elicited by the psychedelic or the cause of other extra-pharmacological factors (e.g. placebo drug, meditation, contextual factors). With fewer intense altered-state phenomena to process, they appeared more attuned to contextual elements, including the retreat setting and interpersonal dynamics. This may explain the higher proportion of setting-related content in the placebo condition and the more balanced distribution of attention across topic areas.

Much of the existing literature has attempted to isolate the therapeutic effects of psychedelics from extra-pharmacological influences. In contrast, our mixed-methods analysis consistently surfaced topics deeply tied to contextual factors embedded in the study design. Notably, both automated and manual analysis revealed a clear divergence in how participants expressed emotional valence: those receiving DMT-harmine directed positive affect toward their psychedelic experiences and associated subjective effects, whereas placebo participants emphasized broader contextual aspects of the retreat, such as music, setting, and interpersonal atmosphere shaping their experiences (e.g. “*Because the music also does something*,* so you just have to be aware of that*,* right?*” or “*Yes*,* as I said*,* just like the music*,* the people are additional aspects*,* additional factors in this whole.*”). This subtle but meaningful difference was captured by emergence of two closely related yet meaningfully differentiated topics: ‘Profound positive affect and uplifted emotional states’ which appeared in the verum-only and across groups analysis, and ‘Profound positive affect and uplifted interpersonal-contextual emotional states’ which emerged in the placebo-only analysis. Although setting-related content was less prominent in the verum group, previous studies suggest an increased sensitivity to environmental factors under psychedelics^21,89^, and other non-psychedelic psychoactive drugs^90^, implying that such influences may have been equally active but less verbally articulated^91,92^.

Contextual factors have recently regained recognition as essential determinants of the intensity and quality of psychedelic experiences^93–96^. Structured, supportive settings were found to be conducive to positive and therapeutically valuable experiences^89,97^, while also buffeting against the negative impact of challenging experiences^98,99^. Moreover, the setting seems to shape emotional breakthrough experiences, which in turn are predictive of positive therapeutic outcomes^84,100^. Recently, Pronovost-Morgan et al. published 30 influencing extra-pharmacological variables and guidelines for reporting of setting of clinical trials with psychedelics; results from an international Delphi consensus study with 89 experts from 17 countries^101^. Our findings support the notion of an intimate interplay between set, setting and subjective effects. Echoing previous research, we argue that psychedelics should not be regarded as standalone pharmacological agents, but rather as context-sensitive catalysts whose therapeutic potential emerges through complex interactions between drug, the immediate and extended environment and an individual’s mindset^3,94,95^.

### Limitations

The present study has several limitations that should be considered. First, phenomenological interviews were conducted within nine days post experience, due to logistical constraints. This provided insight into a broader spectrum of post-experience states while reducing comparability and immediacy, as some participants had already returned to their everyday life when they were interviewed.

Second, utilizing an unsupervised topic modeling approach carries inherent constraints. Without domain-specific training, the model generated some thematically diffuse or incoherent topics, which complicated interpretation and labeling. Although topic labels were the product of an iterative, team-based evaluation process informed by label suggestions from a Large Language Model, the manual refinement inevitably introduced a degree of subjectivity. Additionally, the removal of stop words to improve topic coherence may have exerted thematic influence on the outputs, partially undermining the model’s exploratory potential.

Third, a greater sample size would have generated more text data, thus would have allowed the model to learn from an experientially more diverse dataset. This might have resulted in more coherent, diverse and meaningful topics for the NLP analysis. This consideration is particularly relevant for the placebo-only analysis, given the smaller number of placebo interviews relative to the verum condition. However, given the resource-intensive nature of in-depth phenomenological interviews, we prioritized analytic comparability and ecological validity over dataset expansion. Future studies should explore the use of NLP methods in controlled but large-scale naturalistic settings.

Fourth, our manual text analysis was prone to potential investigator’s bias. Although coders were not involved in NLP analyses, they were aware of experimental assignments, and verum interviews tended to be longer and more vivid. These factors may have influenced coding salience and perceived thematic prominence.

Lastly, the retreat setting itself shaped the content and framing of participants’ experiences. While this enhances ecological validity, it also makes it difficult to disentangle the influence of pharmacological from contextual factors. Thus, our findings should be viewed as exploratory and specific to this experimental context, rather than universally generalizable.

## Conclusion

This study presents a first step toward more qualitative, unbiased, and data-driven investigations of the subjective effects elicited by psychedelics^4,102,103^.

Transformer-based NLP methods (such as BERTopic) complement traditional qualitative methods as they offer a unique sensitivity to experiential facets that often fall outside traditional theory-driven frameworks. These methods can reduce the time and labor demands of conventional approaches substantially, even when applied to smaller, structured datasets.

Our findings underscore the salient interrelation between extra-pharmacological factors and subjective effects, calling for dedicated studies on how contextual influences shape psychedelic experiences, and their potential therapeutic or well-being effects.

Although the present results do not allow for conclusions regarding the therapeutic relevance of specific subjective effects, they do support the hypothesis that such experiences – whether induced by psychedelic substances or meditation – may be central to their therapeutic and wellbeing potential.

More broadly, our findings highlight the importance of embedding subjective experience more firmly within phenomenological frameworks and refining psychometric tools to better capture the therapeutic potential of psychedelics.

## Supplementary Information

Below is the link to the electronic supplementary material.

Supplementary Material 1

Supplementary Material 2

Supplementary Material 3

Supplementary Material 4

Supplementary Material 5

Supplementary Material 6

## Data Availability

For more information on participant characteristics and study design we refer the reader to the primary publication Meling et al., 2024 [49] and the follow-up publication Egger et al. (2025) [104]. Limitations to safeguard sensitive data: Audio recordings, video files, or other personal interview materials are considered sensitive personal data under both Swiss law and GDPR. Even pseudonymization does not meet the high threshold of irreversible anonymization required to fully exempt data from data protection rules. Therefore, these data will not be publicly shared via repositories but will remain confidential. The reader is signposted to Supplementary data for more details on the code and topic system from the manual and automated analysis. The supplement also provides unidentifiable sample quotes and further curated metadata. For specific inquiries please reach out to the first author Jonas Schlomberg (jonas.schlomberg@uzh.ch).
