# Supplementary material for: Mixed-methods analysis on psychedelic-augmented meditation experiences from a randomized controlled mindfulness retreat
Source: Sci Rep. 2026 Mar 18;16:14236. doi: 10.1038/s41598-026-39261-5 (PMC13139390; doi:10.1038/s41598-026-39261-5)

Mixed-Methods Analysis on Psychedelic-Augmented Meditation Experiences from a Randomized Controlled Mindfulness Retreat

Supplementary data I: Participant Characteristics in Comparison Between the DMT-harmine group and Placebo group

	DMT-harmine group	Placebo group	*p*	SMD
**n**	20	20		
**Age (mean (SD))**	41.85 (11.66)	45.60 (8.38)	0.250	0.369
**Sex (%)**				
male	11 (55.0)	11 (55.0)	1.000	<0.001
female	9 (45.0)	9 (45.0)		
**Hours of meditation practice (mean (SD))**	2125.00 (1509.49)	2720.00 (2182.37)	0.322	0.317
**Highest Education level (%)**				
primary school degree	2 (10.0)	1 (5.0)	0.669	0.501
secondary school degree	0 (0.0)	1 (5.0)		
high school degree	1 (5.0)	1 (5.0)		
university degree	16 (80.0)	17 (85.0)		
other school degree	1 (5.0)	0 (0.0)		
**Years of education (mean (SD))**	17.90 (5.32)	18.35 (5.34)	0.791	0.084
**Ethnicity (%)**				
White	19 (95.0)	19 (95.0)	1.000	<0.001
Hispanic	1 (5.0)	1 (5.0)		
**Religious belief (%)**				
Christian	4 (20.0)	3 (15.0)	0.691	0.657
Buddhist	5 (25.0)	3 (15.0)		
Atheist	0 (0.0)	1 (5.0)		
Pantheist/Animist	0 (0.0)	1 (5.0)		
Spiritual, but not religious	7 (35.0)	9 (45.0)		
None	3 (15.0)	3 (15.0)		
Other	1 (5.0)	0 (0.0)		

Values are either given as mean (standard deviation) or as frequency (%) including standard mean difference (SMD) and *p*-value.

Mixed-Methods Analysis on Psychedelic-Augmented Meditation Experiences from a Randomized Controlled Mindfulness Retreat

Supplementary Data II: Python Code for cleaning, standardization and segmentation of interview transcripts

```
import csv
import os
import re
import glob
import spacy
import pandas as pd
from docx import Document

### Function to load .docx files, perform segmentation (sentence-based) and store individual "chunks" in a .csv file. We consider both interviewer and interviewee parts.

# Load the spaCy model
nlp = spacy.load("en_core_web_lg")

# Define function to extract participant_id
def extract_participant_id(line):
    match = re.search(r'DHM-\d{2}', line)
    return match.group() if match else None

# Function to remove silence notations (e.g., '(...)')
def remove_silence_notations(text):
    silence_pattern = re.compile(r'\(\.{2,}\)')
    return re.sub(silence_pattern, '', text)

# Function to remove interviewer's or interviewee's comments (e.g., '//S01: Okay.//')
def remove_comments(text):
    comment_pattern = re.compile(r'//.*?//')
```

```
    return re.sub(comment_pattern, '', text)

# Custom function to clean and process text
def clean_and_segment_text(text):
    text = remove_silence_notations(text)
    text = remove_comments(text)
    processed_doc = nlp(text)
    sentences = [sent.text.strip() for sent in processed_doc.sents if
len(sent.text.strip()) > 10]
    return sentences

# Define main function to combine all processing steps
def process_files(input_directory, output_directory):
    for filename in os.listdir(input_directory):
        if filename.endswith(".docx"):
            filepath = os.path.join(input_directory, filename)
            participant_id = extract_participant_id(filename)
            if not participant_id:
                print(f"Participant ID not found in {filename}, skipping.")
                continue

            doc = Document(filepath)
            text_units = []

            # Iterate through document paragraphs to find relevant segments
            for paragraph in doc.paragraphs:
                if 'S00' in paragraph.text or 'S01' in paragraph.text:
                    cleaned_text = paragraph.text[4:].strip()
                    sentences = clean_and_segment_text(cleaned_text)
```

```
                text_units.extend(sentences)

        # Save processed text units to a CSV file
        if text_units:
            csv_filename = os.path.join(output_directory, f"{filename[:-5]}.csv")
            with open(csv_filename, 'w', newline='', encoding='utf-8') as csvfile:
                writer = csv.writer(csvfile)
                writer.writerow(['Participant-ID', 'Text Unit'])
                for unit in text_units:
                    writer.writerow([participant_id, unit])
            print(f"Processed and saved data for {participant_id} in {csv_filename}")

# Set directories
input_directory = '/File/Path'
output_directory = '/File/Path'

# Run the function
process_files(input_directory, output_directory)
```

Mixed-Methods Analysis on Psychedelic-Augmented Meditation Experiences from a Randomized Controlled Mindfulness Retreat

Supplementary Data III: Python Code for topic modeling analysis with BERTopic – code framework was used for all three topic modeling analyses

```
import pandas as pd
import re
from umap import UMAP
from hdbscan import HDBSCAN
import stanza
import nltk
nltk.download('stopwords')
from nltk.corpus import stopwords
from sklearn.feature_extraction.text import CountVectorizer
from bertopic.vectorizers import ClassTfidfTransformer
from bertopic.representation import TextGeneration, MaximalMarginalRelevance
from bertopic import BERTopic

### load data

data = pd.read_csv('/FILE/PATH')


# create document list

docs = data['Text Unit'].tolist()                # This is the column containing the text segments

id  = data['Participant-ID'].values.tolist()        # This is the column containing the ids



# Step I


## Pre-calculate vector embeddings

from sentence_transformers import SentenceTransformer

embedding_model = SentenceTransformer("sentence-transformers/paraphrase-multilingual-mpnet-base-v2")
```

```
embeddings = embedding_model.encode(docs, show_progress_bar=True)

# Step II

## Reduce dimensionality
umap_model = UMAP(
    n_neighbors=15,
    n_components=5,
    min_dist=0.0,
    metric='cosine',
    random_state=42)

# Step III

## Cluster reduced embeddings
hdbscan_model = HDBSCAN(
    min_cluster_size=35,
    min_samples=10,
    metric='euclidean',
    cluster_selection_method='eom',
    prediction_data=True)

# Step IV and V

german_stop_words = stopwords.words('german') # https://github.com/stopwords-
iso/stopwords-de

# Extension of the stopword list (translated into English from German)
german_stop_words.extend([  # time references
```

'jahr', 'woche', 'tag', 'zeit', 'periode', 'monat',

'stunde', 'minute', 'jederzeit', 'morgen', 'nachmittag', 'mittag', 'abend',

'september',

weekdays

'montag', 'dienstag', 'mittwoch', 'donnerstag', 'freitag', 'samstag', 'sonntag',

indefinite articles

'der', 'die', 'das',

standard filler expressions

'einfach', 'bisschen', 'irgendwie', 'vielleicht', 'eigentlich', 'wirklich','eur',

'schon',

saying goodby and hello

'Tschüss', 'ciao', 'Hallo', 'Hi', 'Bis Bald',

experimental group / study medication

'DMT', 'Harmin', 'DMT/Harmin', 'Gruppe', 'psilocybin', 'dmt',

speaker identifiers

'S00', 'S01',

filler words

'mhm', 'ähm', 'ehm', 'uh', 'oh', 'aha', 'ah', 'wow', 'äh', 'ach', 'hä',

single-word answers

'ja', 'nein', 'mmh', 'Mmh', 'Mmh-Mmh', 'mmh-mmh', 'okay', 'eben', 'mega',

'selbstverständlich', 'möglicherweise', 'ne', 'ok', 'okay', 'super', 'wow',

words specific to context of zoom/phone meeting

'auflegen', 'zoom', 'telefon', 'telefonieren', 'email', 'meeting', 'gespräch',

'gruß', 'unterbrich', 'reformulieren',

'danke', 'dank', 'teilnahme',

```
                    # travel-related words
                    'abfahren', 'Berlin', 'rückgereist', 'Anreise', 'Abreise', 'tram',
                    # words attributable to interviewer
                    'inwiefern', 'dissertation', 'studie', 'daten', 'forschung', 'erachtens',
                    'frage', 'fall',
                    # domain-specific but not contributing
                    'erfahrung', 'gefühl', 'low', 'dose', 'behörde', 'apotheke', 'substanz',
                    'publikation', 'mittwochsgruppe', 'studienwoche', 'follow',
                    'up', 'psychometrie',
                    # Noise
                    'rhein', 'eert', 'dame', 'gravier', 'geldfrage', 'geld', 'buch', 'erfahrng',
                    'freundin', 'kolumbianerin',
                    'moment', 'sache', 'afro', 'amerikanerin', 'diätempfehlung',
'anschiss',
                    'ausführn', 'cool', 'dasa', 'wesensker', 'arbeitswoche',
                    'termin', 'name', 'haar', 'mädchen', 'windel', 'oment', 'unterbrich',
'scheiß',
                    'good', 'apropo',
                    'tschuß', 'oka', 'kuchen', 'alkoholkonsum'
])
# Initialize German pipeline
stanza.download('de')
nlp = stanza.Pipeline('de')
def lemma(text, allowed_postags=['NOUN', 'ADJ']):        # only nouns and adjectives
   text = re.sub(r'\d+', '', text)                    # removal of white space
   text = re.sub(r'[,.!?+:-=`<>"/!()&%]', ' ', text)    # removal of punctuation
   doc = nlp(text)
   text = ' '.join([word.lemma for sent in doc.sentences for word in sent.words if
word.upos in allowed_postags]) # lemmatization using stanza
   text = text.lower() # change all words to lower case
```

```
    return text

# Vectorizer
vectorizer_model= CountVectorizer(stop_words=german_stop_words,
preprocessor=lemma) # remove stopwords and apply lemmatizer

# Step VI

### Representation Model
representation_model = MaximalMarginalRelevance(diversity=0.3)

# Step VII

### Calculate topic representation scores (Importance Scores)
ctfidf_model = ClassTfidfTransformer(reduce_frequent_words=True)

### Set the model
topic_model = BERTopic(
  # Sub-models
  embedding_model=embedding_model,
  umap_model=umap_model,
  hdbscan_model=hdbscan_model,
  vectorizer_model=vectorizer_model,
  representation_model=representation_model,
  ctfidf_model = ctfidf_model,
  calculate_probabilities=True,
  # Hyperparameters
  nr_topics="auto",
  top_n_words=10,
```

```
  n_gram_range=(1, 2),
  verbose=True,
  language="auto"
)

### Train model
topics, probs = topic_model.fit_transform(docs, embeddings)

# Step VIII

### Merging topics with similar content and keywords
topics_to_merge = [[1,13],
                  [0,7],
                  [23,34],
                  [2,3,9,10,19,24,25,26],
                  [30,36]
                 ]

topic_model.merge_topics(docs, topics_to_merge)

# Step IX

### Updating the topics and probabilities
import hdbscan
topics= topic_model._map_predictions(topic_model.hdbscan_model.labels_)
probs = hdbscan.all_points_membership_vectors(topic_model.hdbscan_model)
probs = topic_model._map_probabilities(probs, original_topics=True)

# Step X
```

```
### Assigning custom topic labels

custom_labels_ = {
    -1: "Custom Label Topic -1",
    0: "Custom Label Topic 0 ",
    […]
}
topic_model.set_topic_labels(custom_labels_)

## Additional Analysis Steps

# Step XI

### Visualization and Model Inspection

# Visualize topic distribution in 2-dimensional space (Intertopic Distance Map)
topic_model.visualize_topics()

topics_dict = topic_model.get_topics()
topic_id = 26
all_words = topics_dict[topic_id]  # list of (word, score) pairs
top_15_words = all_words[:15]
print(top_15_words)

# Step XII

### Save (Meta) Data
```

```
### Export keywords and associated importance score for all detected topics
topics_data = []
for topic in topic_model.get_topics().keys():
    if topic in [-1, 0]:  # Skip unwanted topics
        continue

    # Use the original dictionary to get the label
    topic_label = custom_labels_.get(topic, f"Topic {topic}")

    for word, score in topic_model.get_topic(topic):
        topics_data.append({"topic": topic_label, "keyword": word, "c-TF-IDF score":
score})

df_topics = pd.DataFrame(topics_data)
df_topics.to_csv("keywords_across_german.csv", index=False)

### add info about the percentage of the document that relates to the topic
topic_distr, _ = topic_model.approximate_distribution(docs, batch_size=1000)
distributions = [distr[topic] if topic != -1 else 0 for topic, distr in zip(topics, topic_distr)]

### create document dataframe using the original dataframe and metadata about
the topic distributions
df_doc_info = topic_model.get_document_info(
    docs,
    df=data,
    metadata={"Topic_distribution": distributions}
    )

### export as csv (adjust name!!)
df_doc_info.to_csv('/File/Path/across_topic_info.csv')
```

```
df_doc_info


### convert probs 2D array to dataframe
df_topic_probs = pd.DataFrame(probs)

df_topic_probs = df_topic_probs.add_prefix("topic_")
df_topic_probs = df_topic_probs.add_suffix("_prob")

### export as csv
df_topic_probs.to_csv('/File/Path/across_probs.csv')

df_topic_probs


# Step XIII

# Model Performance

### Define metrics for model performance

from sklearn.metrics.pairwise import cosine_similarity
import numpy as np
import itertools

# Function to extract top words for each topic
def get_top_words(topic_model, num_words=10):
    top_words = {}
```

```
    for topic_id, topic in topic_model.get_topics().items():
        if topic_id == -1:
            continue  # Exclude outliers
        top_words[topic_id] = [word for word, _ in topic[:num_words]]
    return top_words

# Function to calculate Topic Diversity
def calculate_topic_diversity(top_words):
    all_words = list(itertools.chain(*top_words.values()))
    unique_words = set(all_words)
    total_words = len(all_words)
    return len(unique_words) / total_words

# Function to calculate Topic Coherence using embeddings
def calculate_topic_coherence(top_words, embedding_model, docs):
    coherences = []
    for topic_id, topic in top_words.items():
        print(f"Calculating coherence for topic {topic_id}")
        pairs = list(itertools.combinations(topic, 2))
        coherence = []
        for w1, w2 in pairs:
            try:
                w1_embedding = embedding_model.encode([w1])[0]
                w2_embedding = embedding_model.encode([w2])[0]
                similarity = cosine_similarity([w1_embedding], [w2_embedding])[0][0]
                coherence.append(similarity)
              #  print(f"Coherence for pair ({w1}, {w2}): {similarity}")
            except Exception as e:
                print(f"Error with pair ({w1}, {w2}): {e}")
```

```
        if coherence:
            coherences.append(np.mean(coherence))
        else:
            coherences.append(np.nan)
    return np.nanmean(coherences)

### Calculate metrics and output the results

# Extract top words for each topic
top_words = get_top_words(topic_model, num_words=10)

# Calculate Topic Diversity and Topic Coherence for the Topic Model
topic_diversity = calculate_topic_diversity(top_words)
topic_coherence = calculate_topic_coherence(top_words, embedding_model, docs)

print(f"Model I - Topic Diversity: {topic_diversity}, Topic Coherence:
{topic_coherence}")
```

Mixed-Methods Analysis on Psychedelic-Augmented Meditation Experiences from a Randomized Controlled Mindfulness Retreat

Supplementary Data VI: Topic Frequency Distributions for all three topic modeling analyses: Verum group (A), Placebo group (B) and Across groups (C).

A

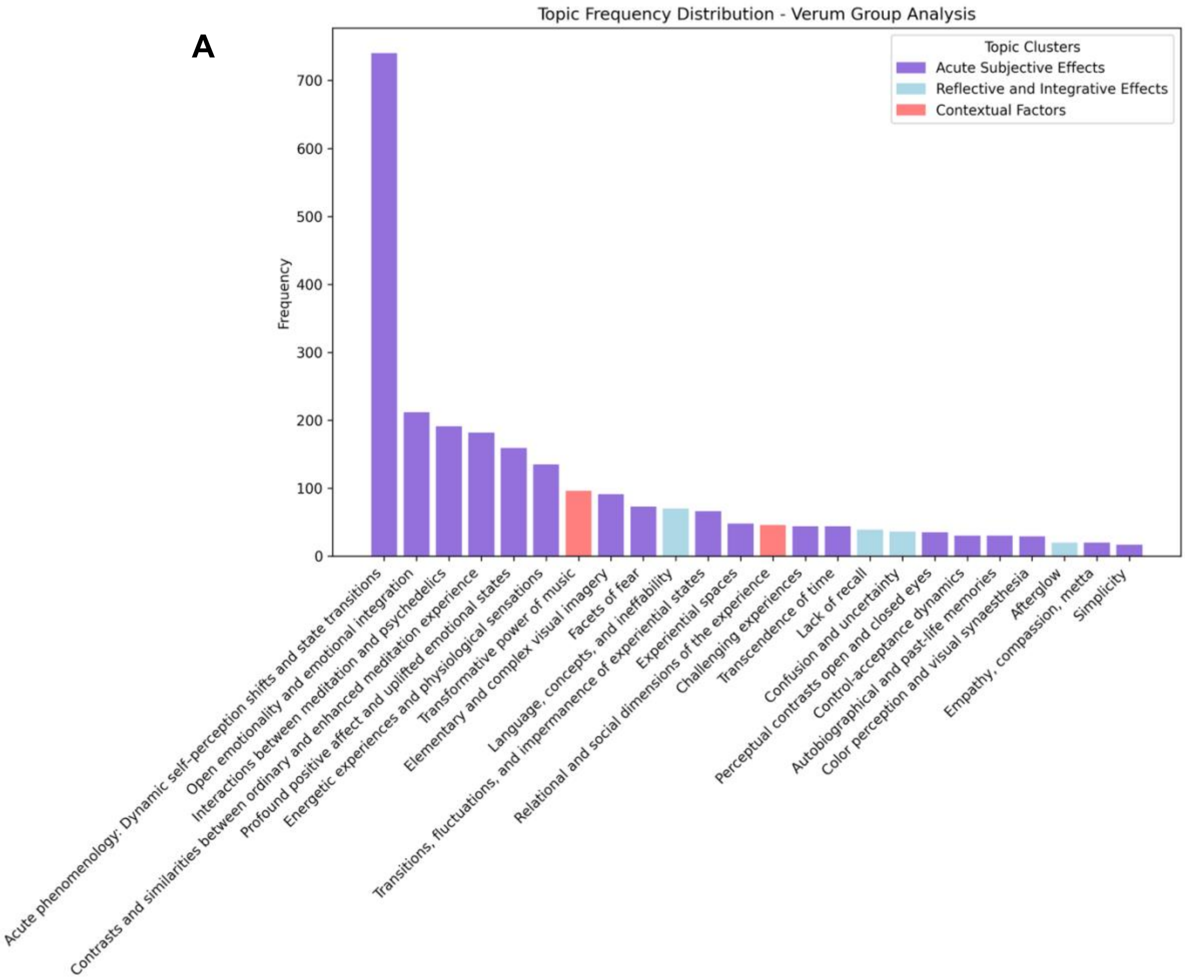

B

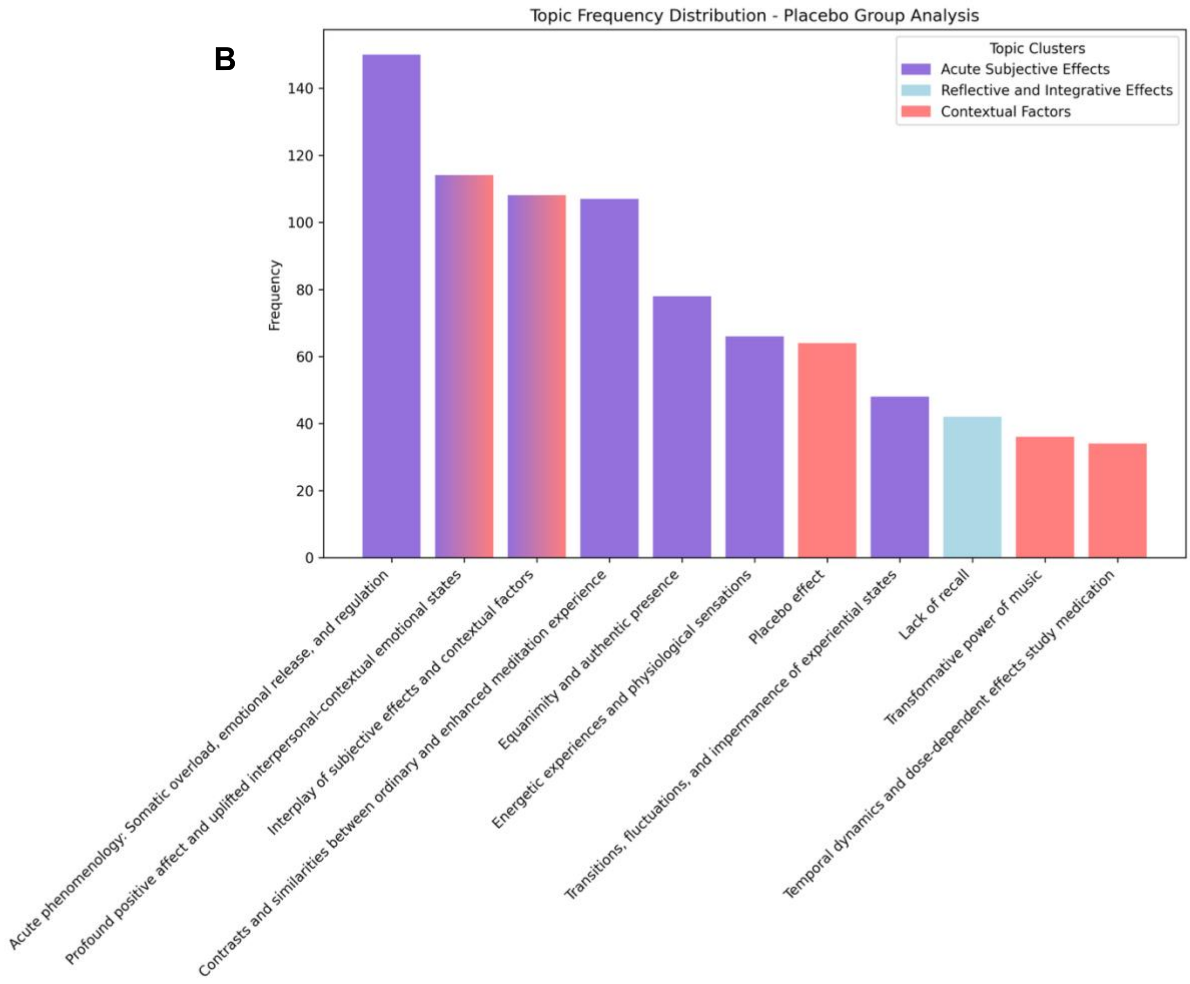
Topic Frequency Distribution - Placebo Group Analysis
Topic Clusters
Acute Subjective Effects
Reflective and Integrative Effects
Contextual Factors
Frequency
0
20
40
60
80
100
120
140
Acute phenomenology: Somatic overload, emotional release, and regulation
Profound positive affect and uplifted interpersonal-contextual emotional states
Interplay of subjective effects and contextual factors
Contrasts and similarities between ordinary and enhanced meditation experience
Equanimity and authentic presence
Energetic experiences and physiological sensations
Placebo effect
Transitions, fluctuations, and impermanence of experiential states
Lack of recall
Transformative power of music
Temporal dynamics and dose-dependent effects study medication

C

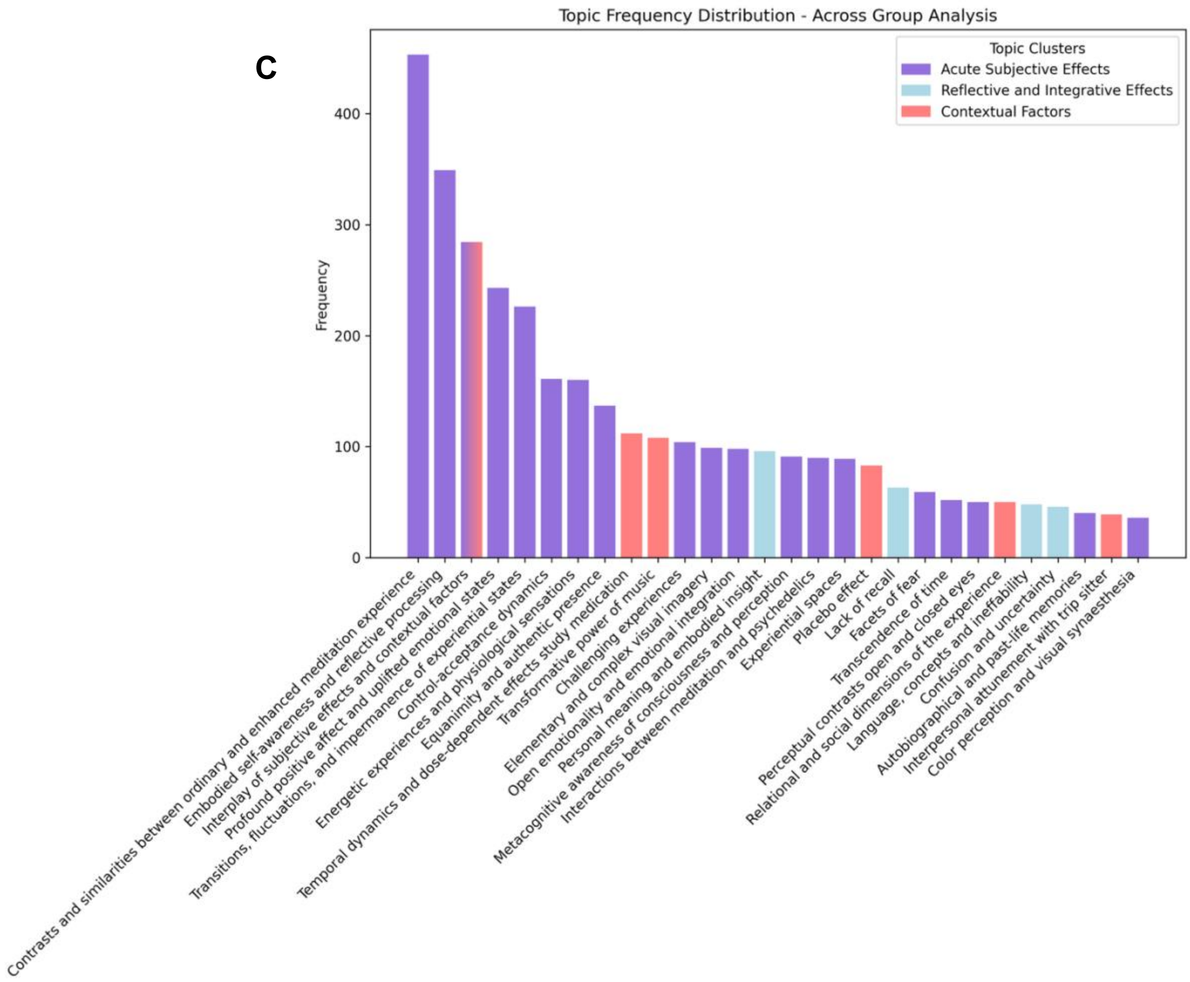
Topic Frequency Distribution - Across Group Analysis
Topic Clusters
Acute Subjective Effects
Reflective and Integrative Effects
Contextual Factors
Frequency
0
100
200
300
400
Contrasts and similarities between ordinary and enhanced meditation experience
Embodied self-awareness and reflective processing
Interplay of subjective effects and contextual factors
Profound positive affect and uplifted emotional states
Transitions, fluctuations, and impermanence of experiential states
Control-acceptance dynamics
Energetic experiences and physiological sensations
Equanimity and authentic presence
Temporal dynamics and dose-dependent effects study medication
Transformative power of music
Challenging experiences
Elementary and complex visual imagery
Open emotionality and emotional integration
Personal meaning and embodied insight
Metacognitive awareness of consciousness and perception
Interactions between meditation and psychedelics
Experiential spaces
Placebo effect
Lack of recall
Facets of fear
Transcendence of time
Perceptual contrasts open and closed eyes
Relational and social dimensions of the experience
Language, concepts and ineffability
Confusion and uncertainty
Autobiographical and past-life memories
Interpersonal attunement with trip sitter
Color perception and visual synaesthesia

Mixed-Methods Analysis on Psychedelic-Augmented Meditation Experiences from a Randomized Controlled Mindfulness Retreat

Supplementary data VII: Word cloud representations of unique keywords for all 28 distinct topics from across-group analysis

Contrasts and similarities between ordinary and enhanced meditation experience

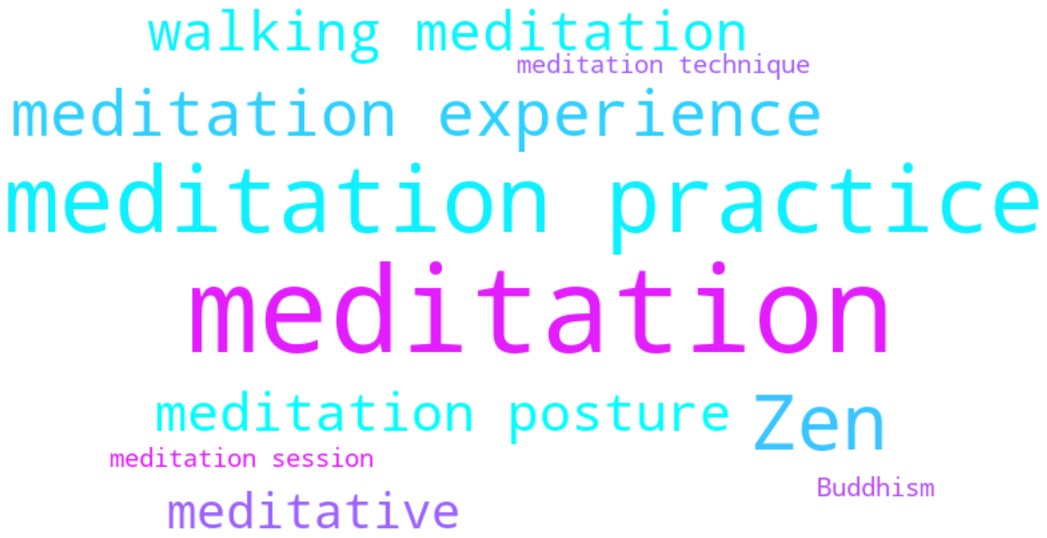

Embodied self-awareness and reflective processing

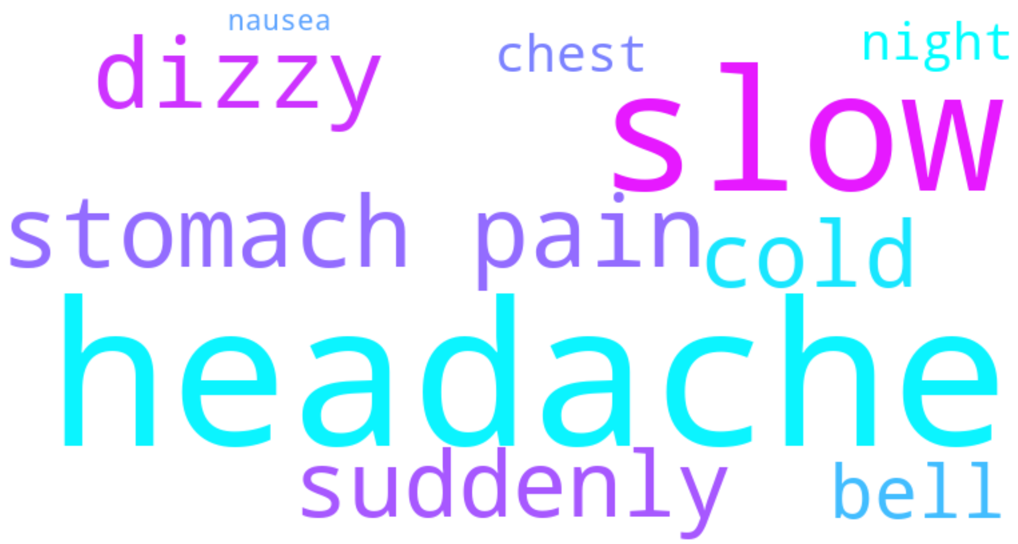

Interplay of subjective effects and contextual factors

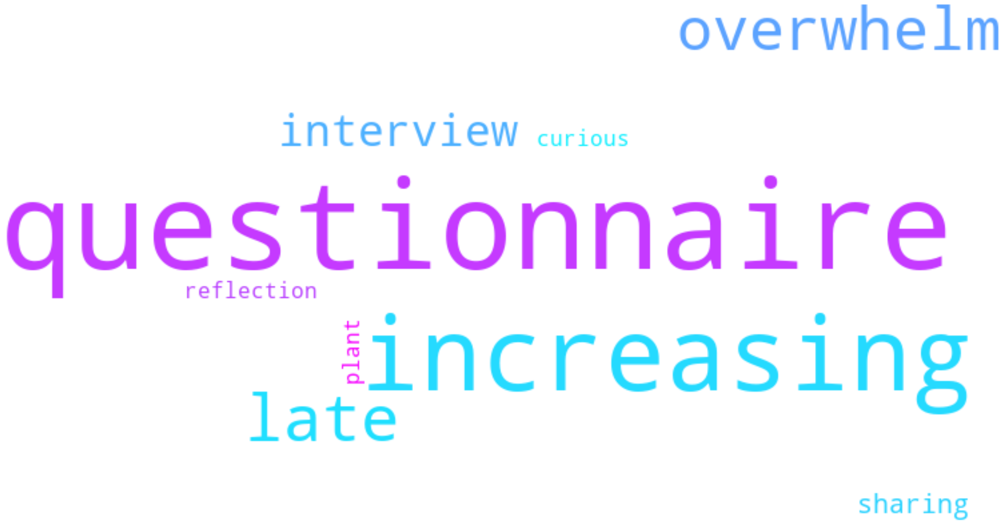

Profound positive affect and uplifted emotional states

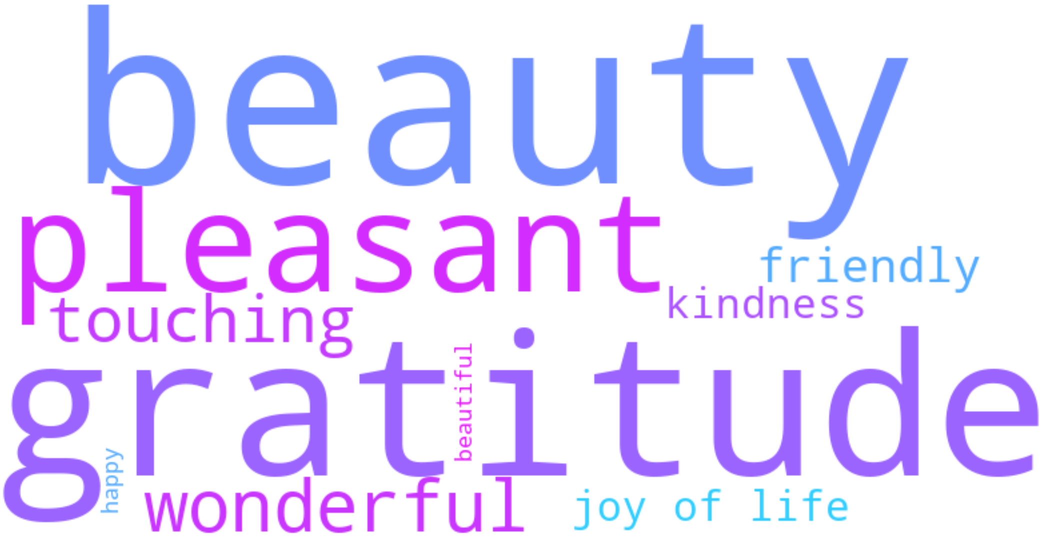

Transitions, fluctuations, and impermanence of experiential states

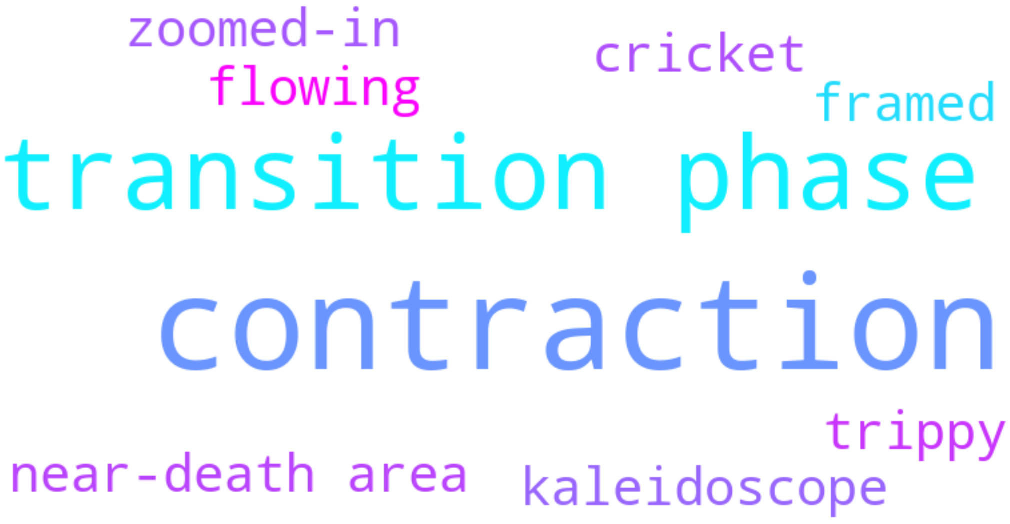

Control-acceptance dynamics

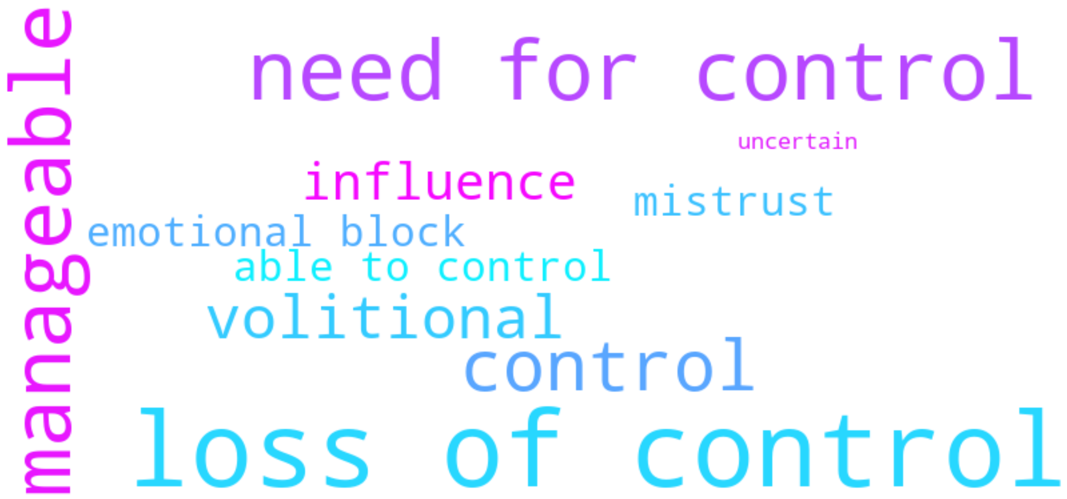

Energetic experiences and physiological sensations

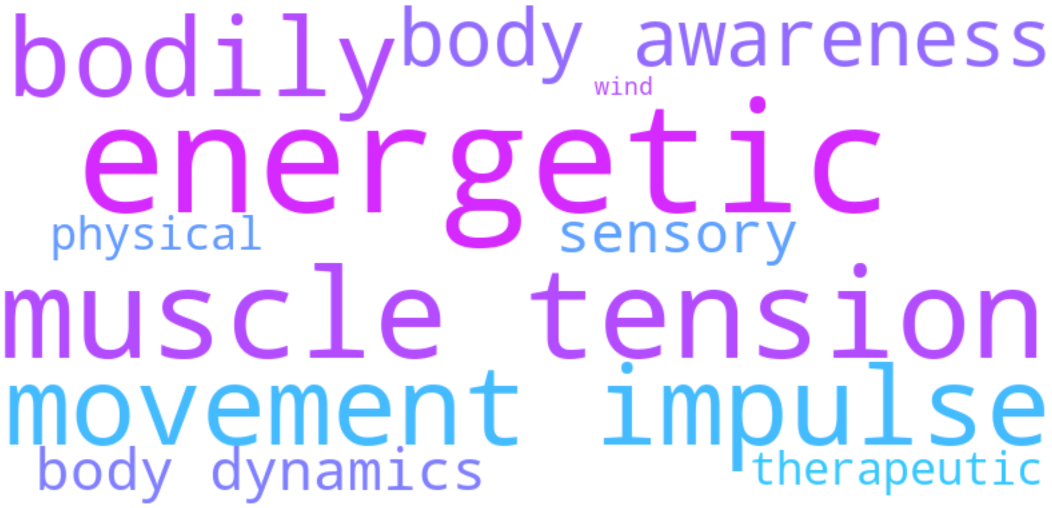

Equanimity and authentic presence

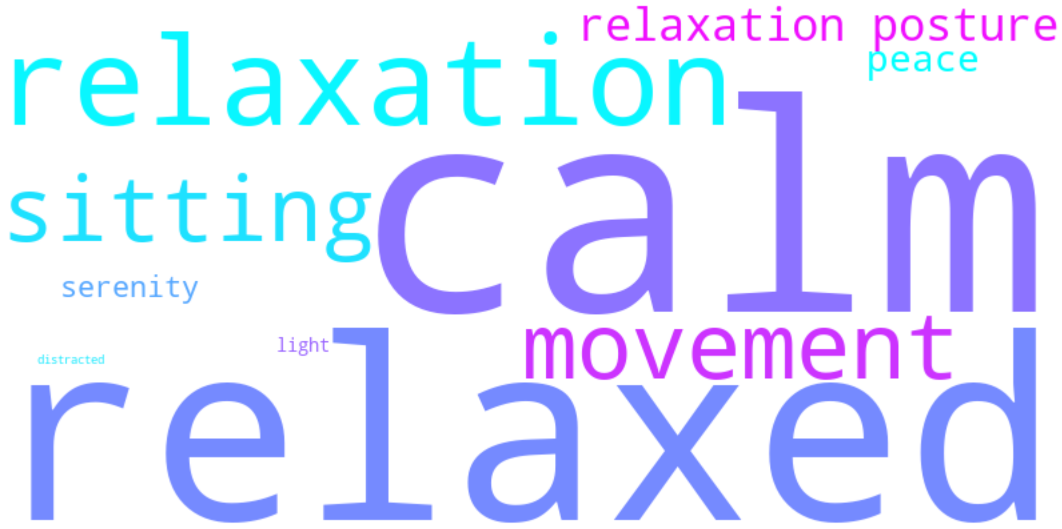

Temporal dynamics and dose-dependent effects study medication

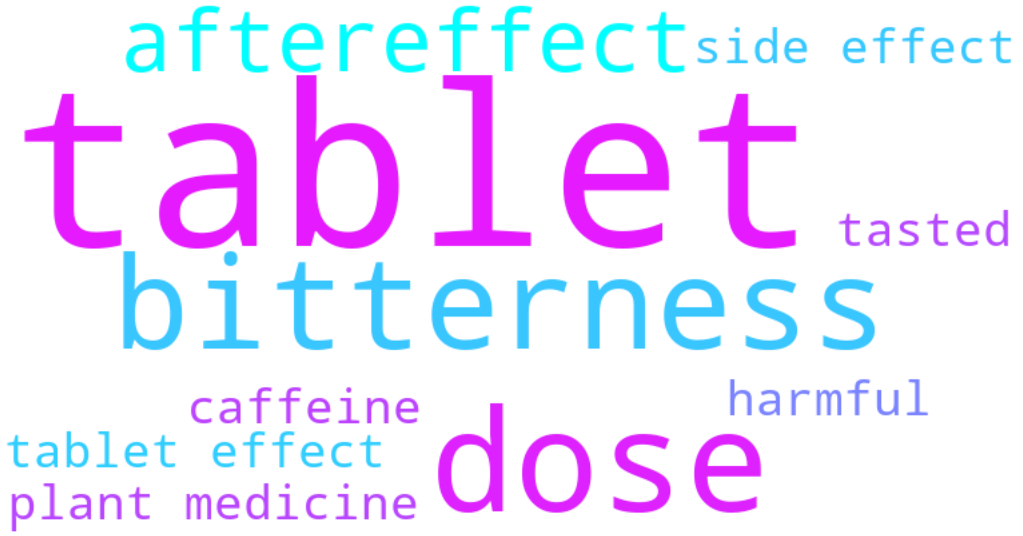

Transformative power of music

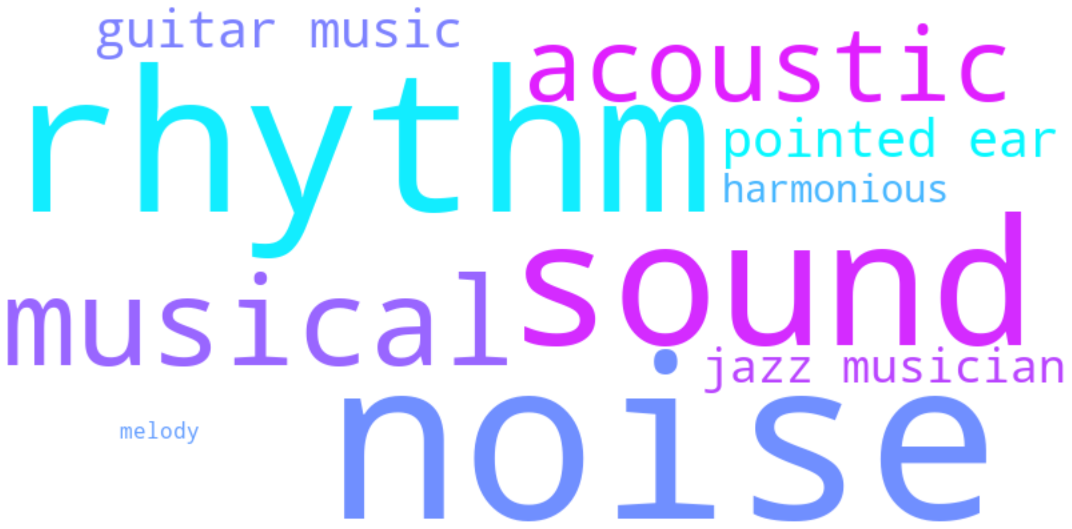

Challenging experiences

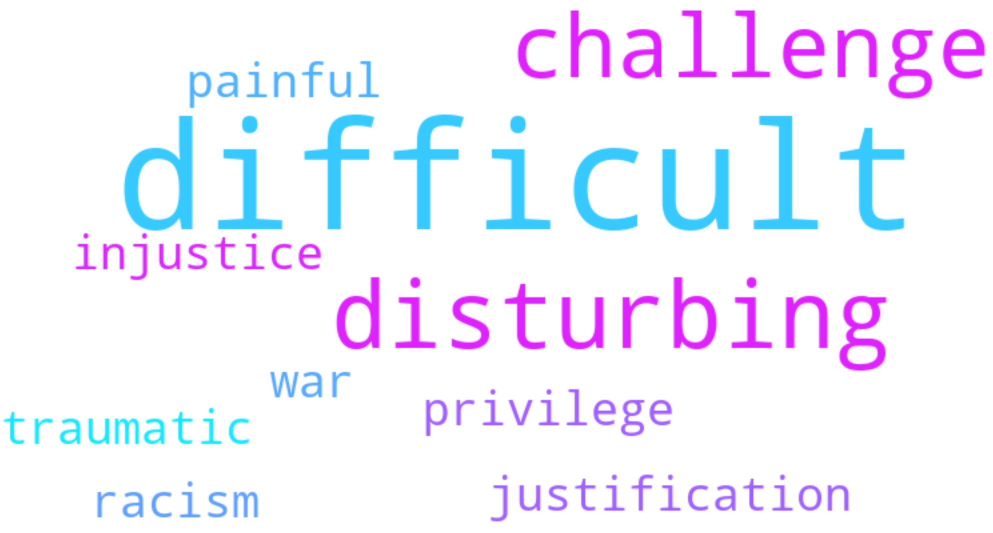

Elementary and complex visual imagery

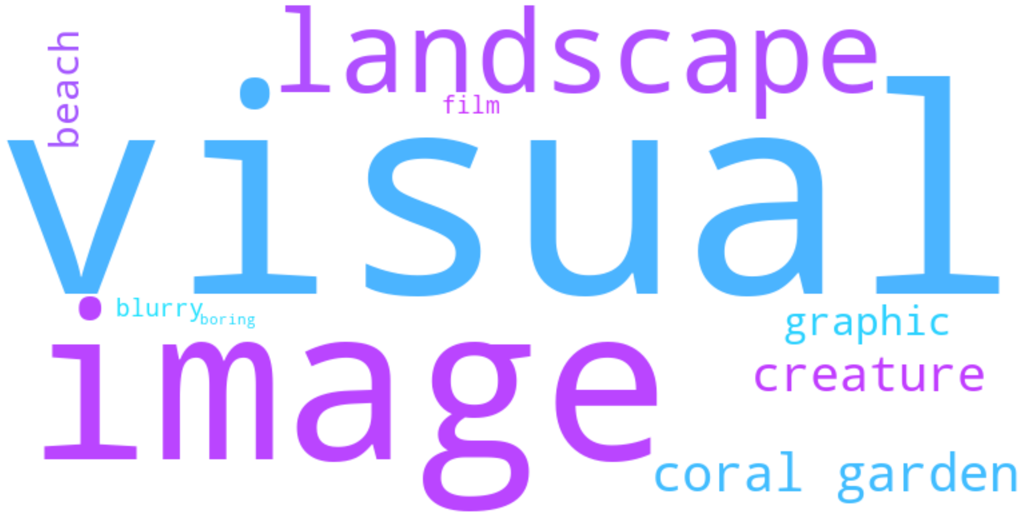

Open emotionality and emotional integration

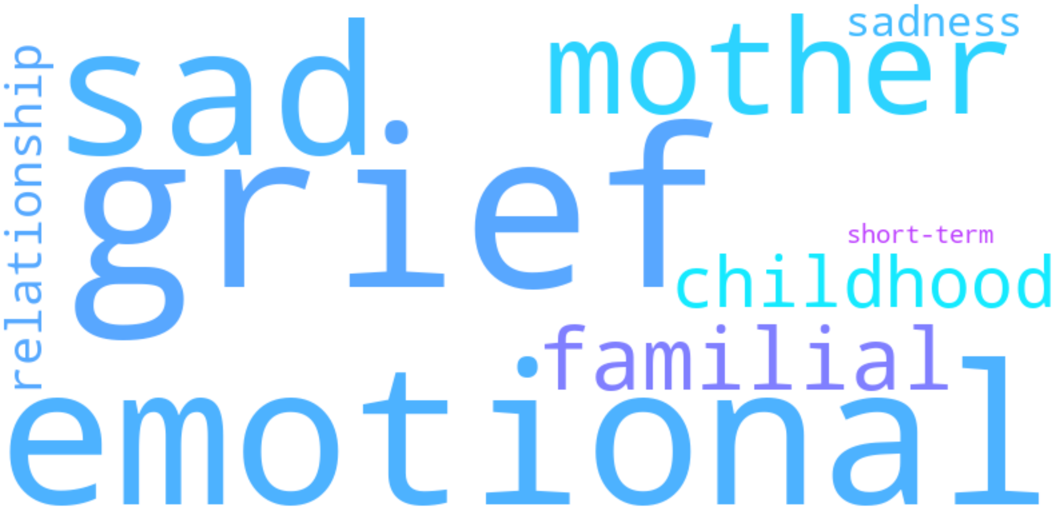

Personal meaning and embodied insight

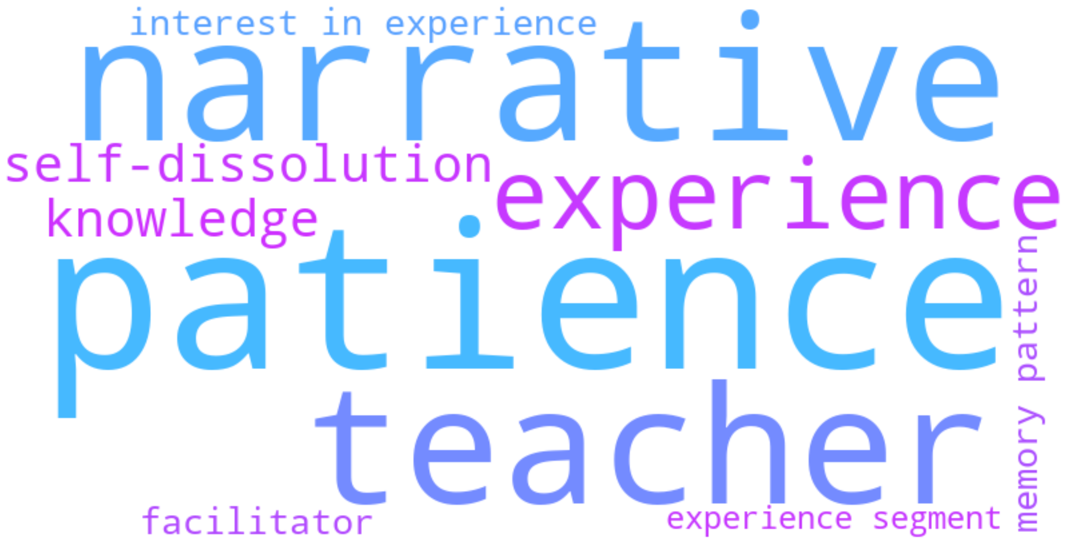

Metacognitive awareness of consciousness and perception

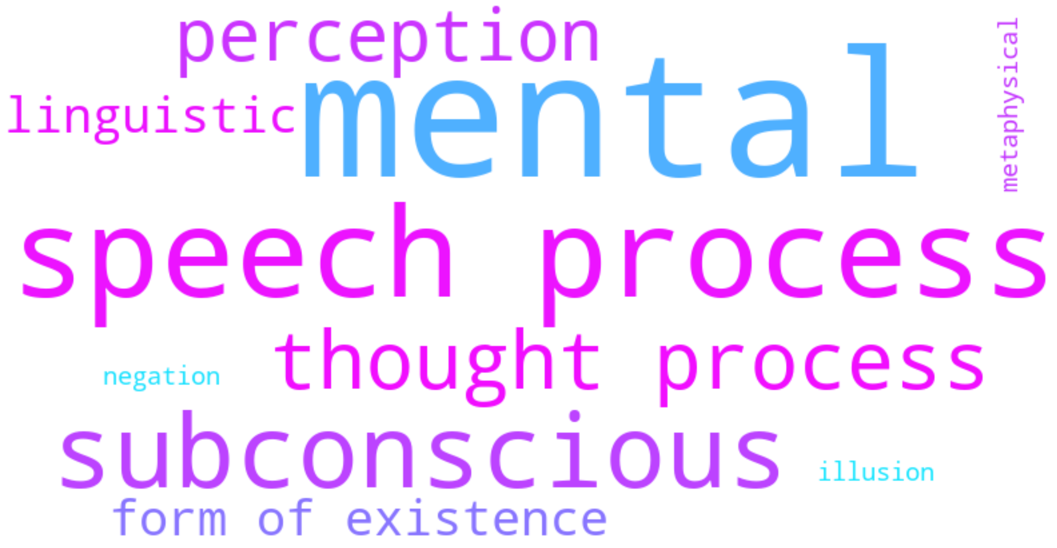

Interactions between meditation and psychedelics

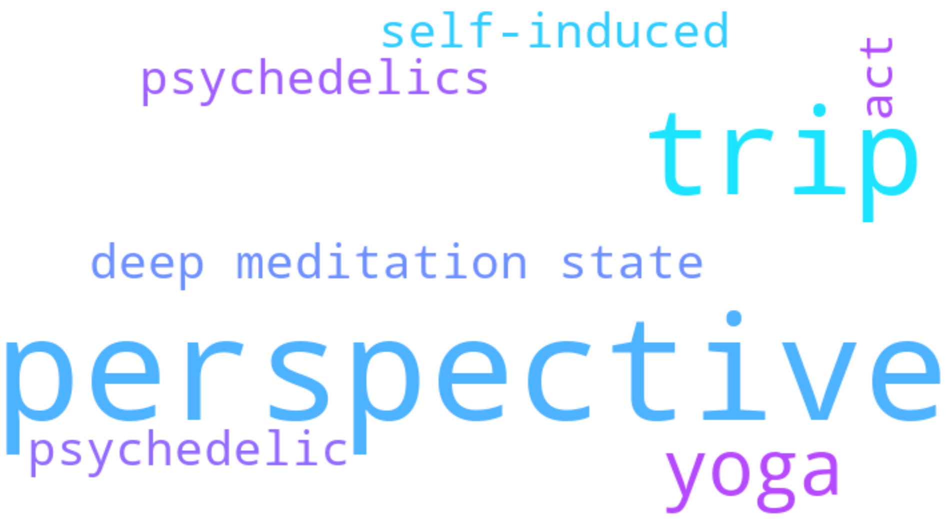

Experiential spaces

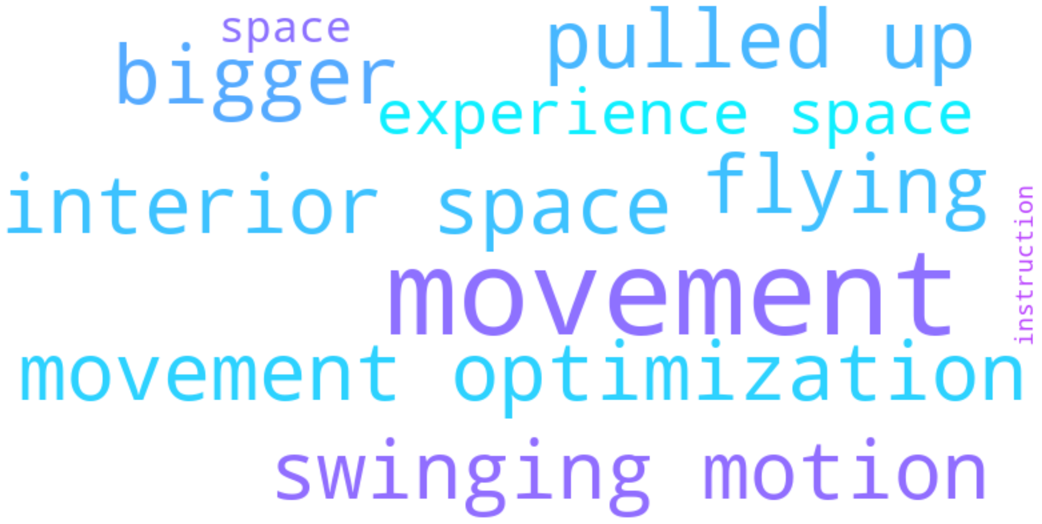

Placebo effect

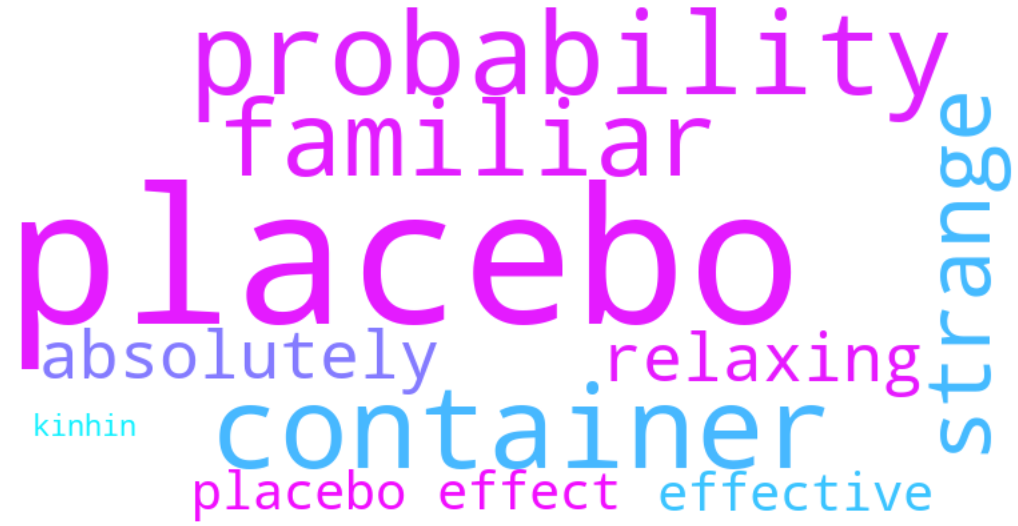

Lack of recall

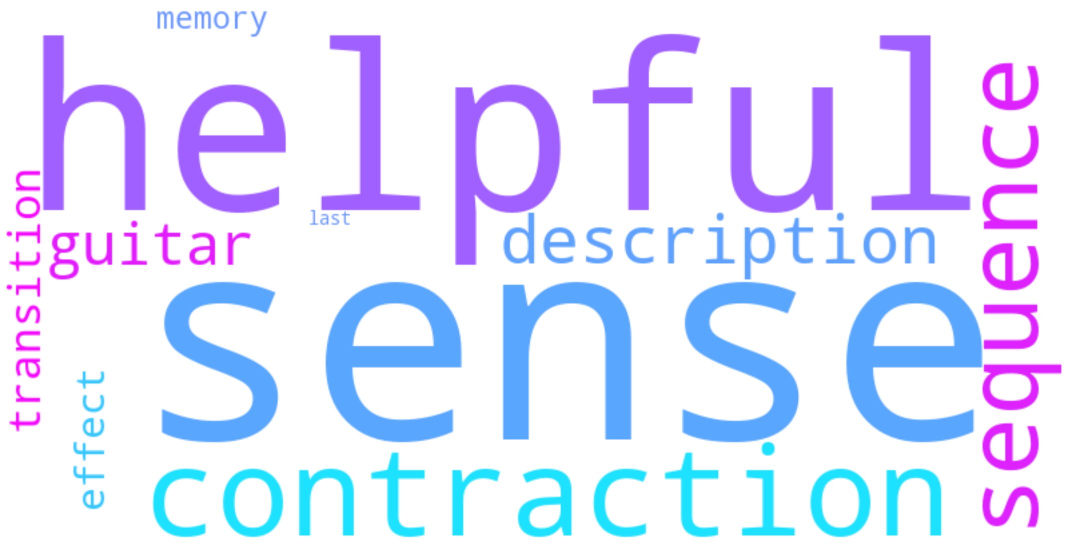

Facets of fear

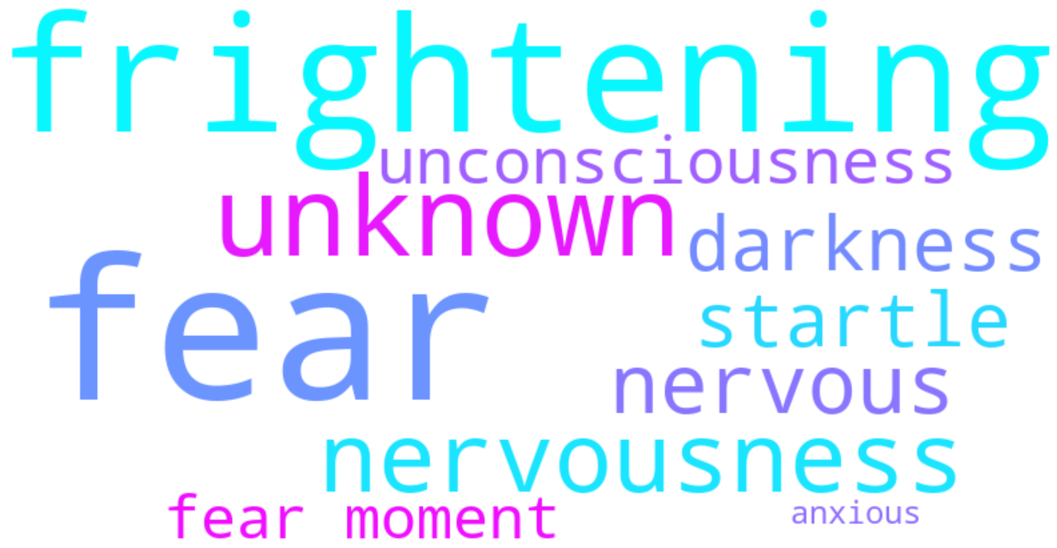

Transcendence of time

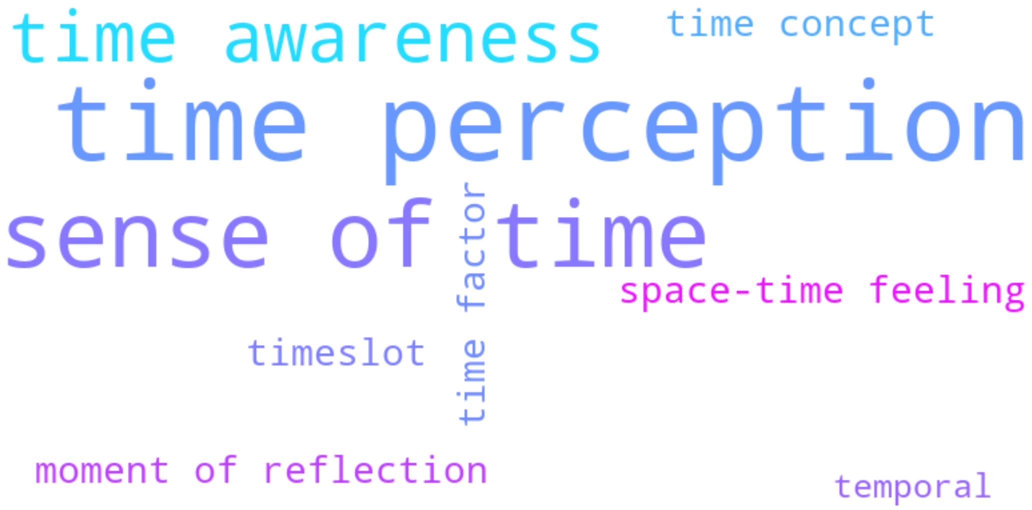

Perceptual contrasts open and closed eyes

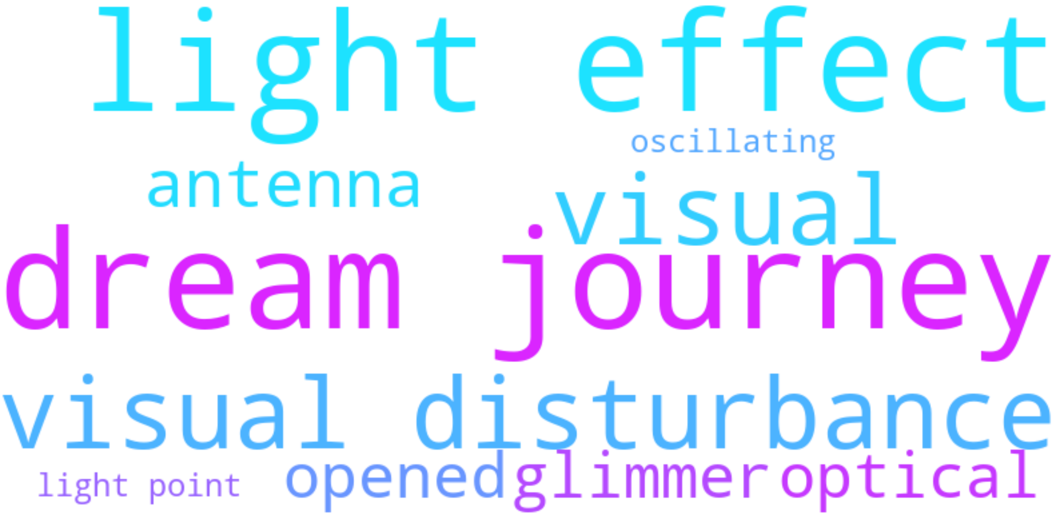

Relational and social dimensions of the experience

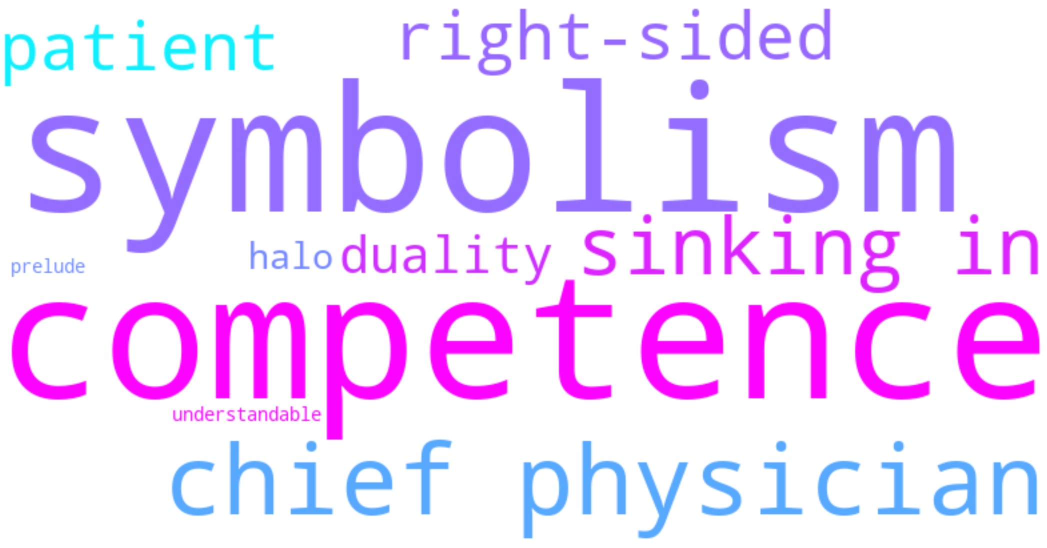

Language, concepts and ineffability

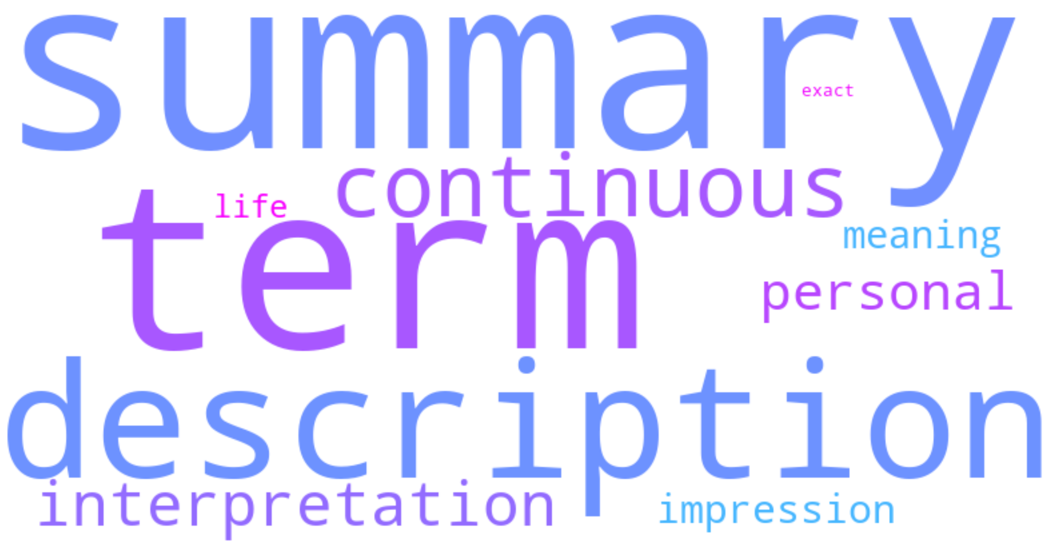

Confusion and uncertainty

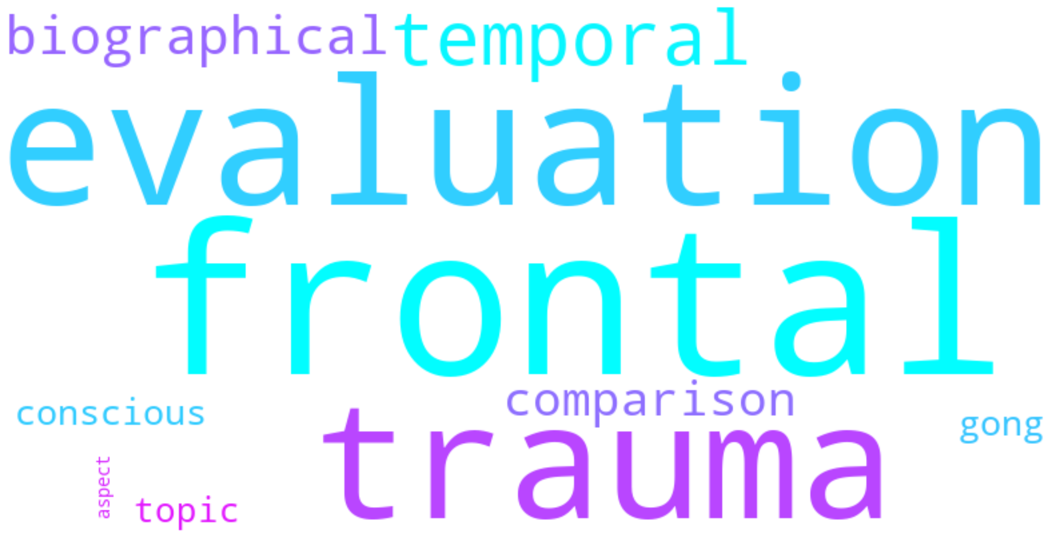

Autobiographical and past-life memories

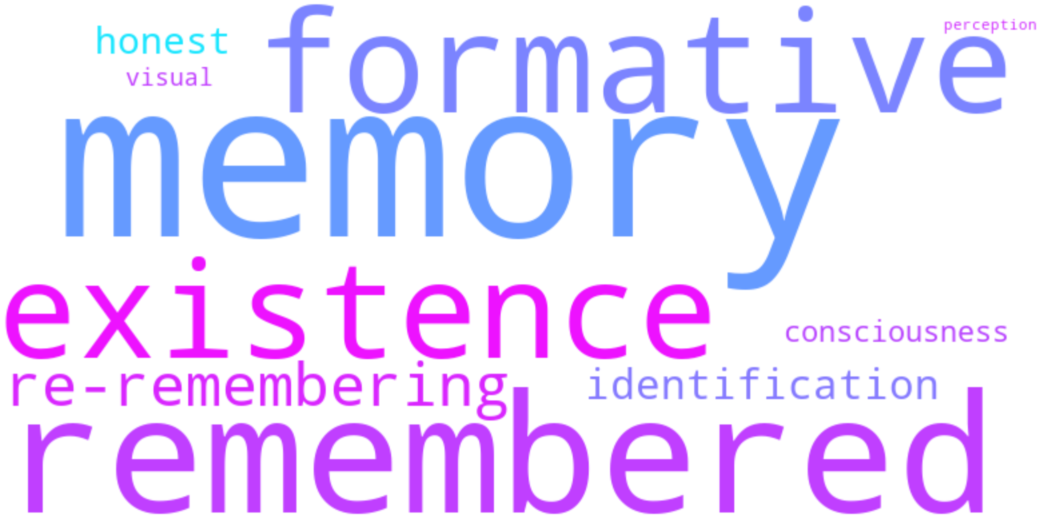

Interpersonal attunement with trip sitter

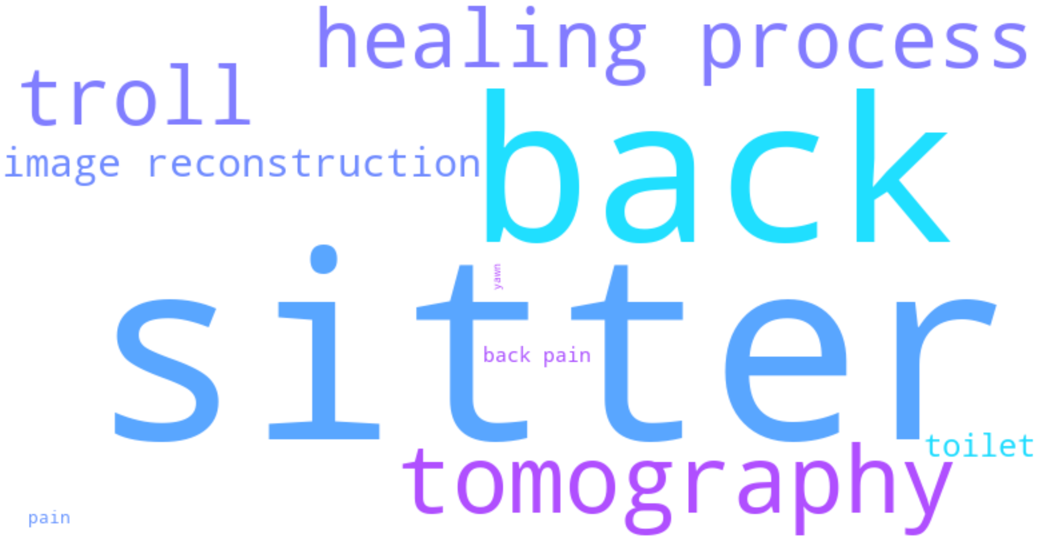

Color perception and visual synaesthesia

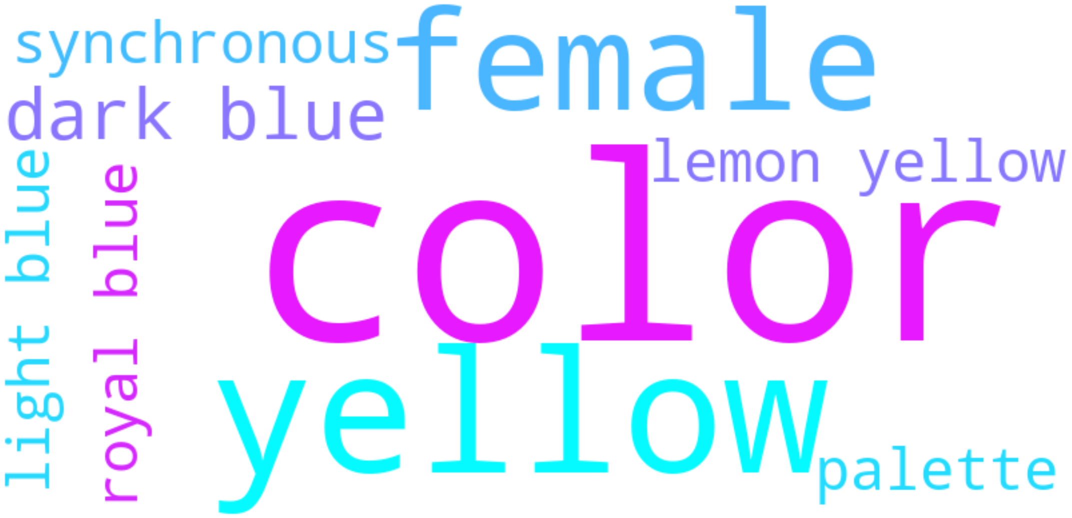

Mixed-Methods Analysis on Psychedelic-Augmented Meditation Experiences from a Randomized Controlled Mindfulness Retreat

Supplementary data IX: Word cloud representations of unique keywords for all 24 distinct topics from verum-group analysis

Acute phenomenology: Dynamic self–perception shifts and state transitions

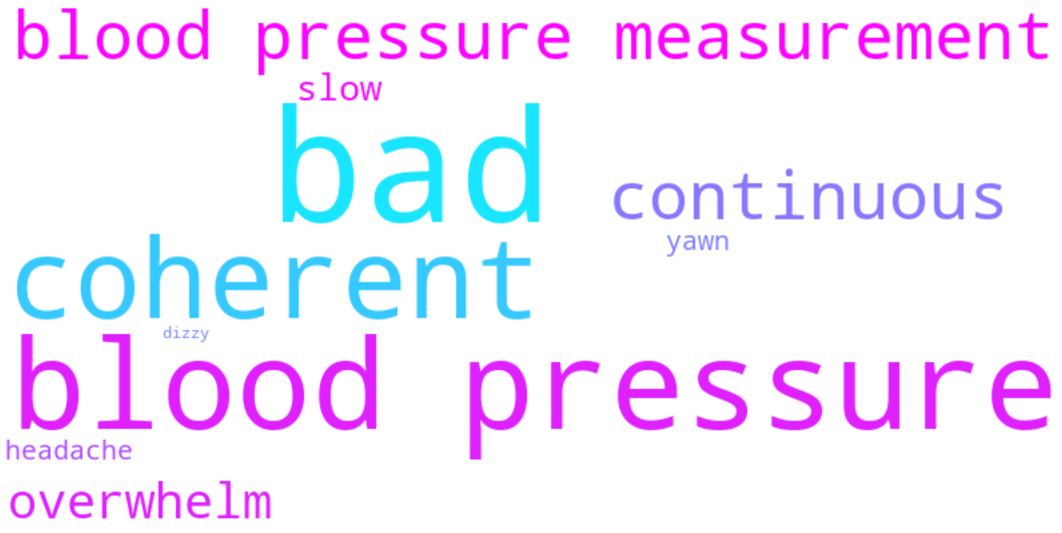

Open emotionality and emotional integration

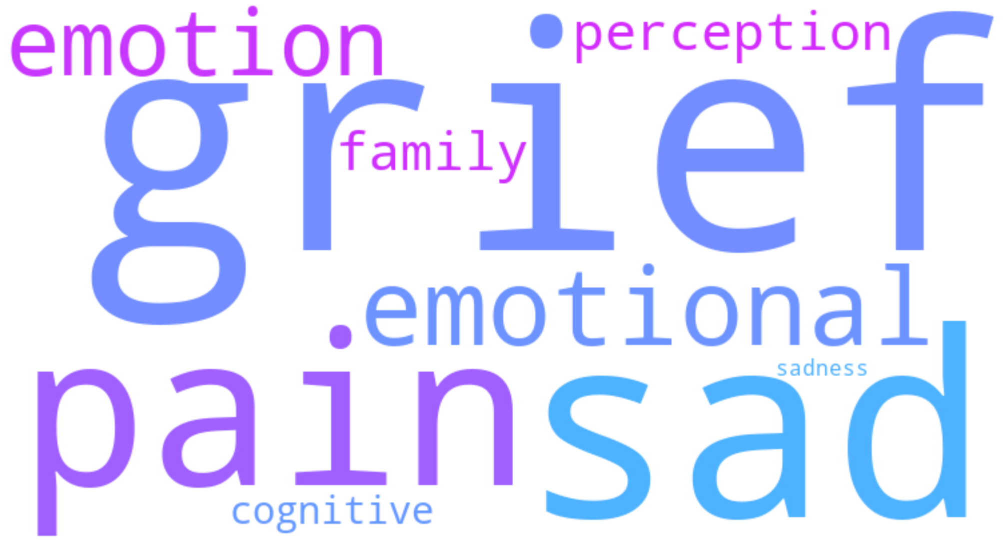

Interactions between meditation and psychedelics

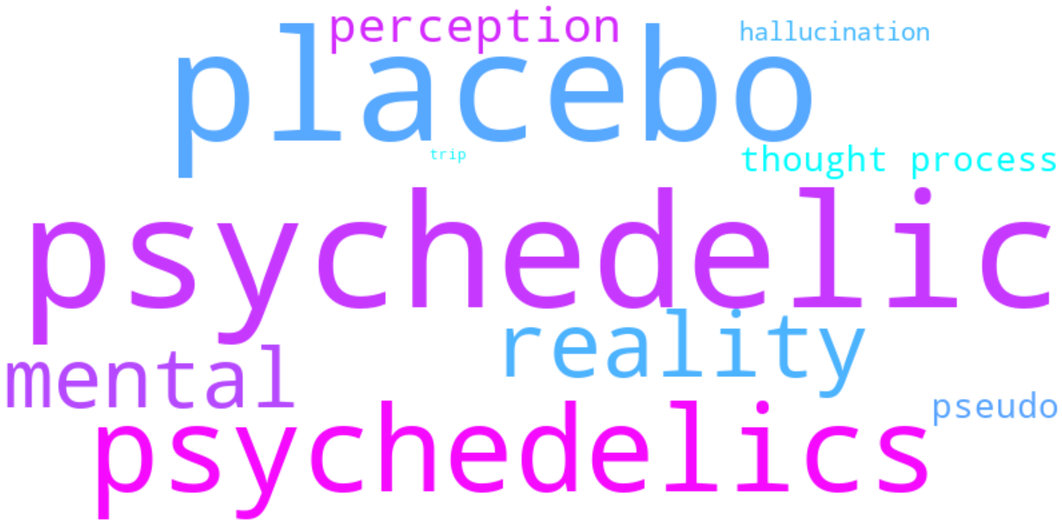

Contrasts and Similarities between Ordinary and Enhanced Meditation Experience

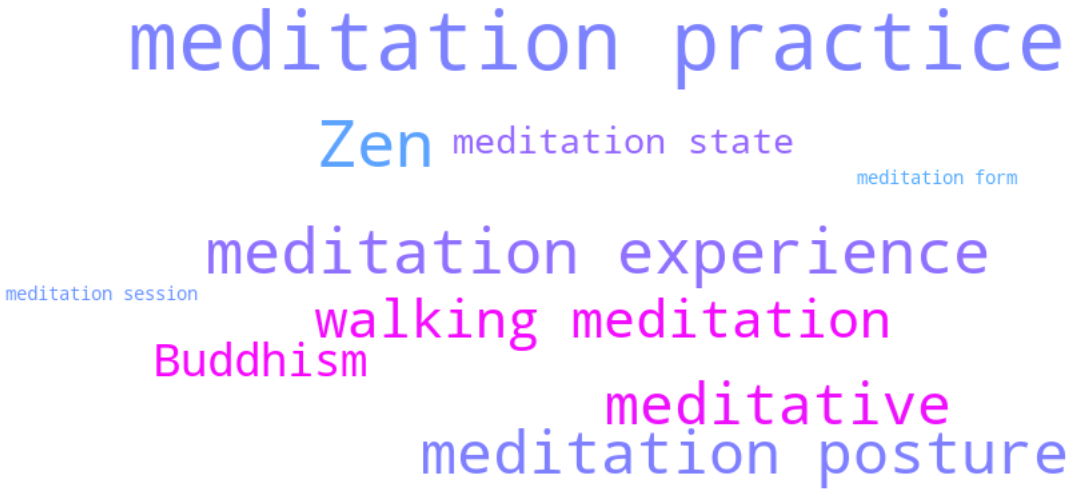

Profound positive affect and uplifted emotional states

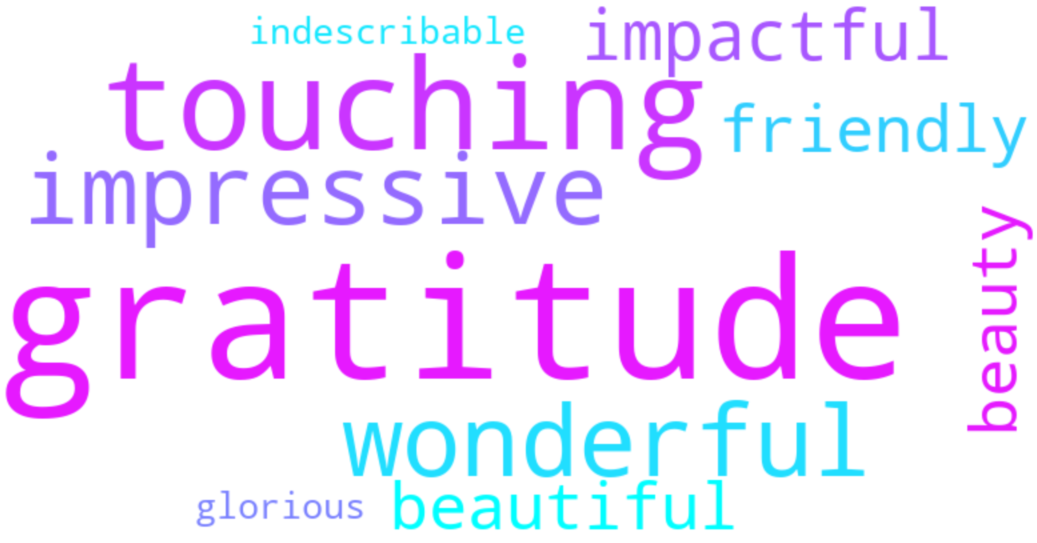

Energetic experiences and physiological sensations

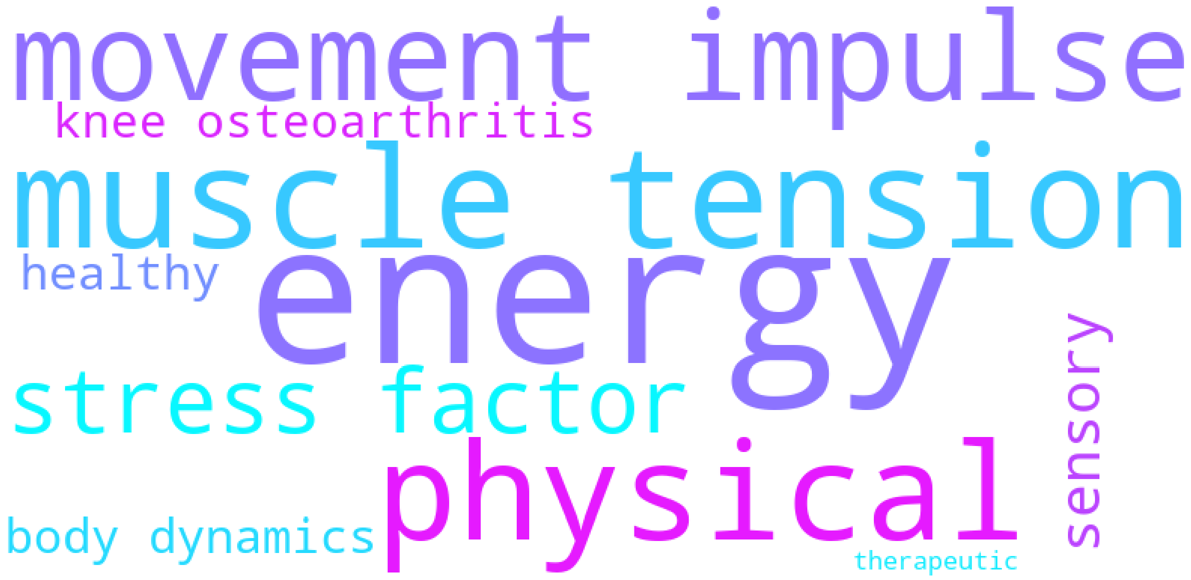

Transformative power of music

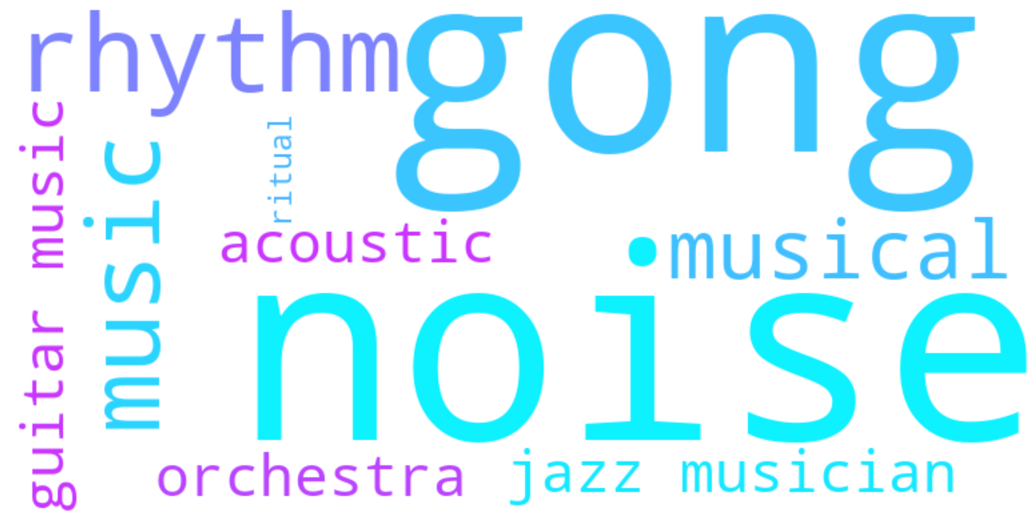

Elementary and complex visual imagery

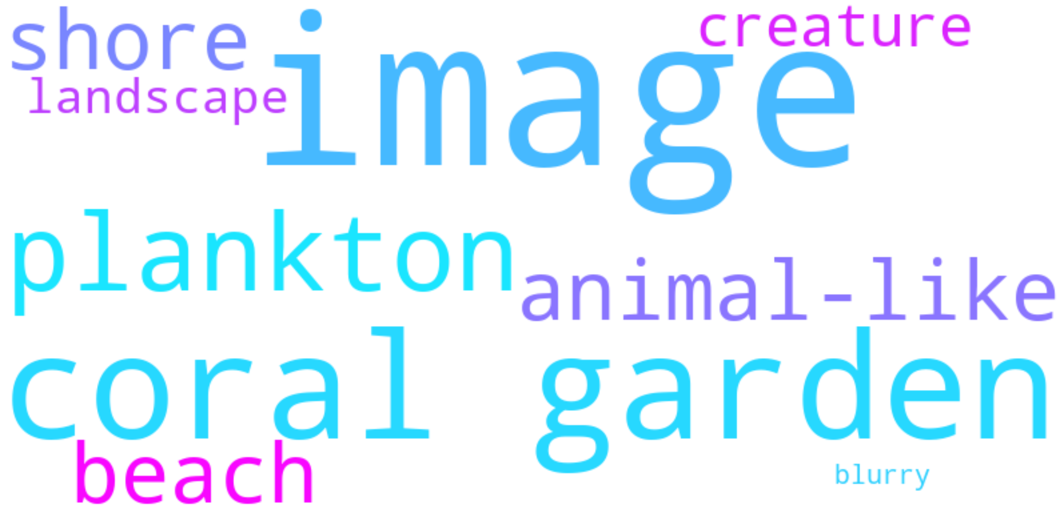

Facets of fear

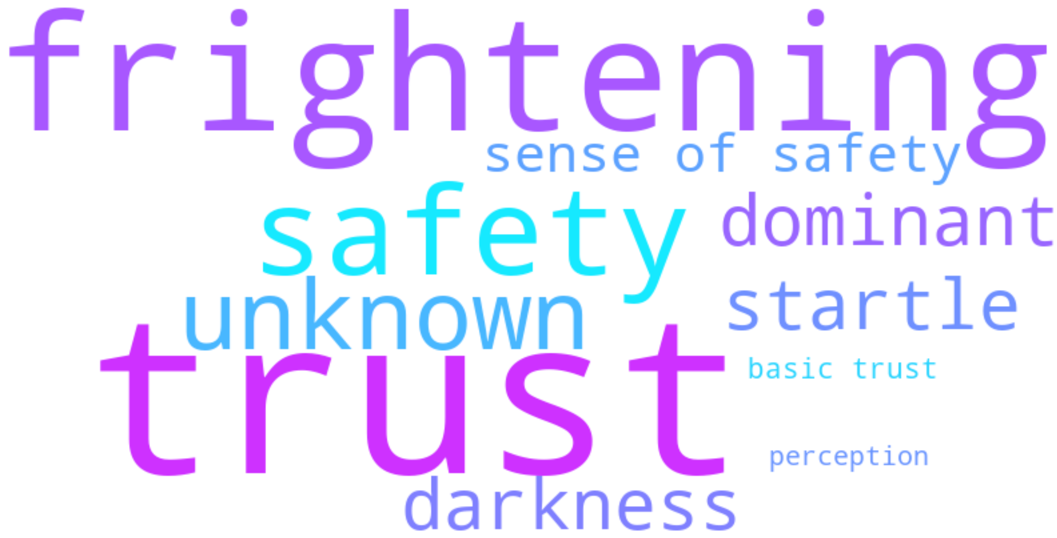

Language, concepts, and ineffability

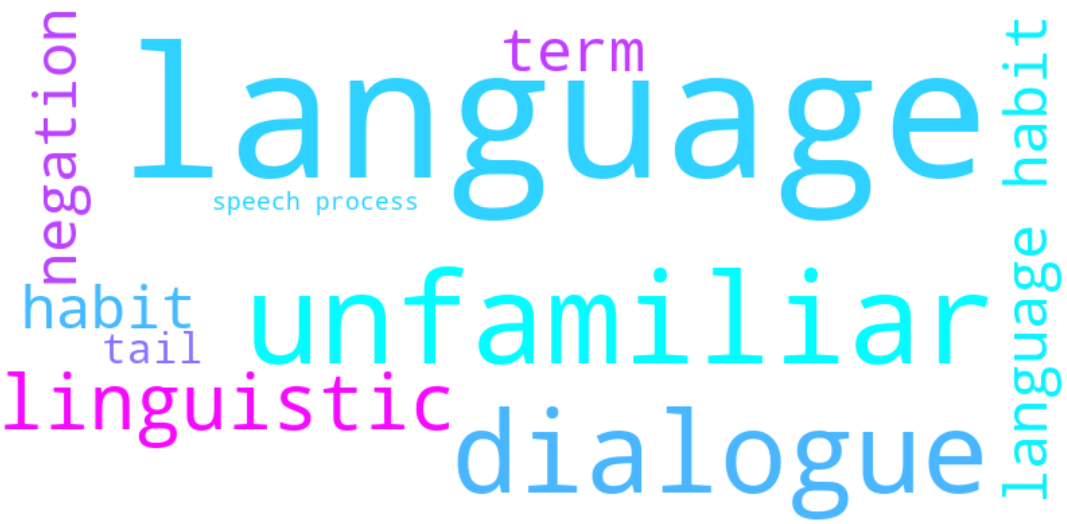

Transitions, fluctuations, and impermanence of experiential states

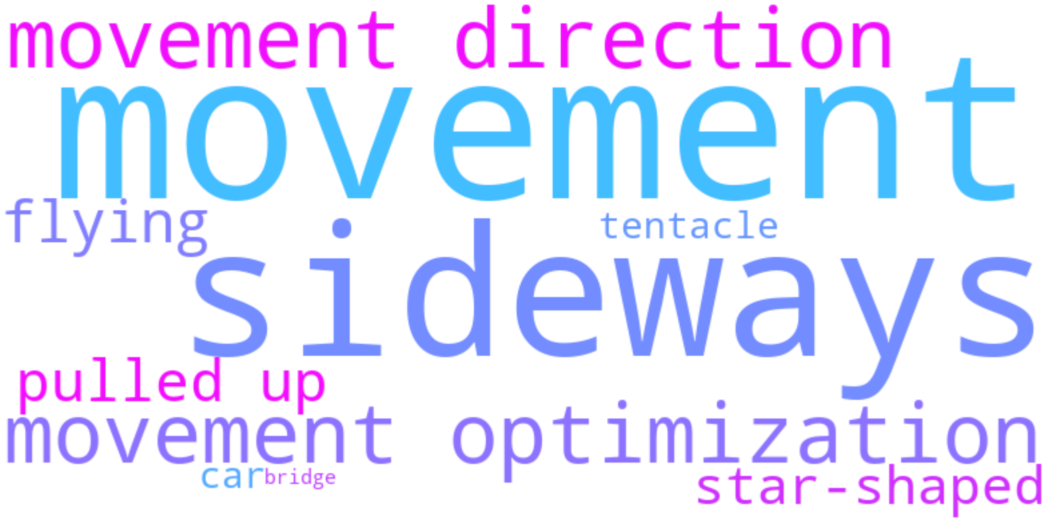

Experiential spaces

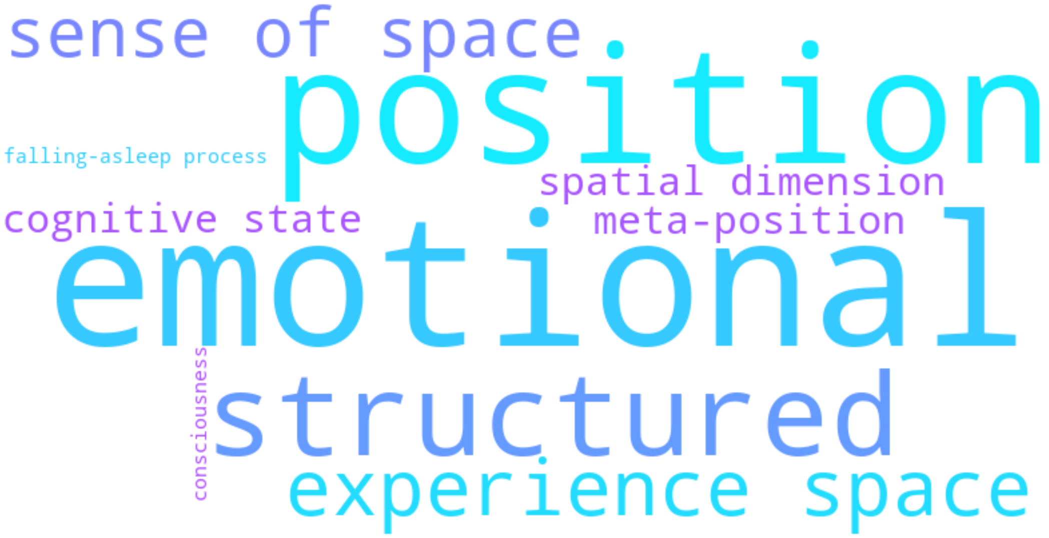

Relational and social dimensions of the experience

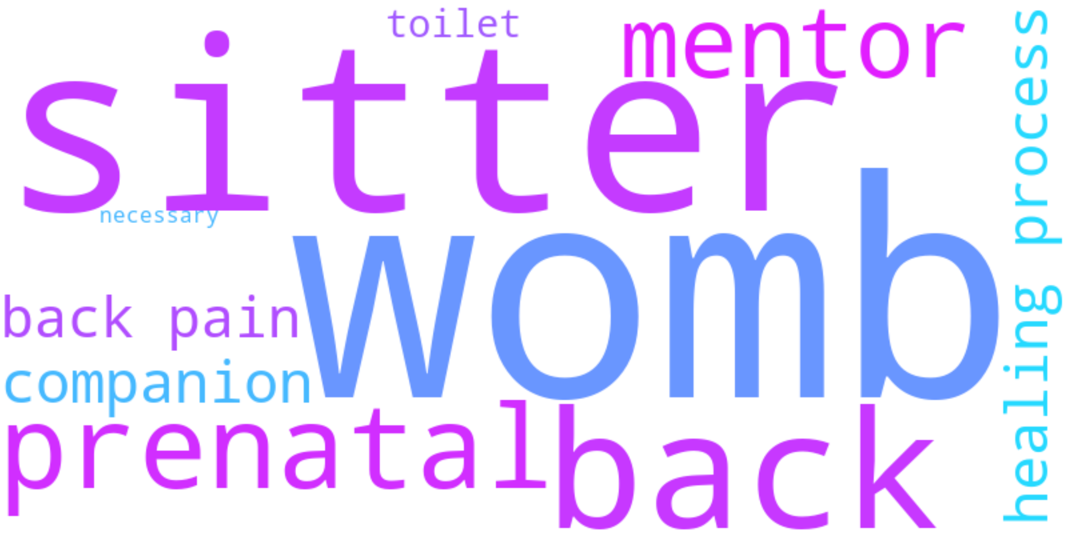

Challenging experiences

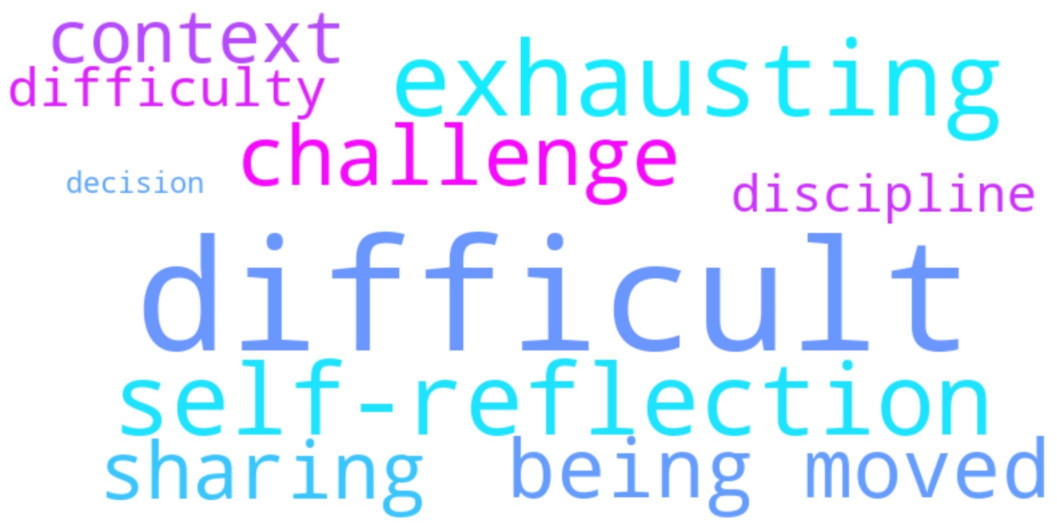

Transcendence of time

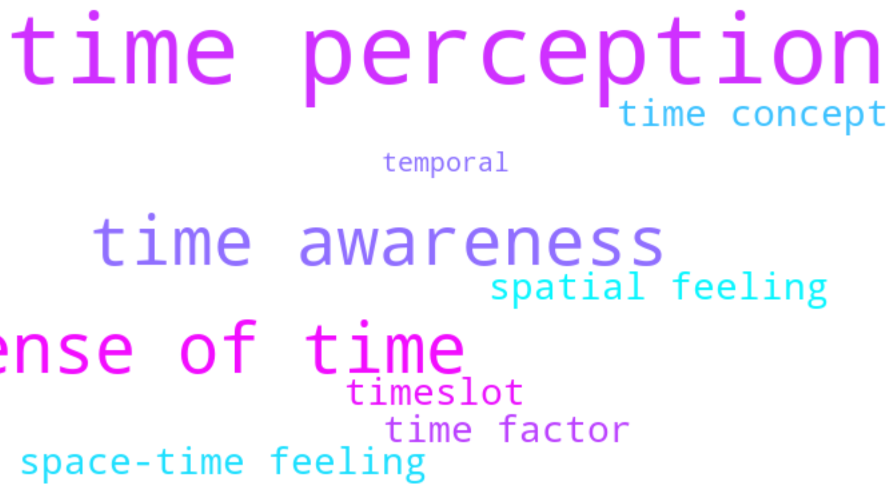

Lack of recall

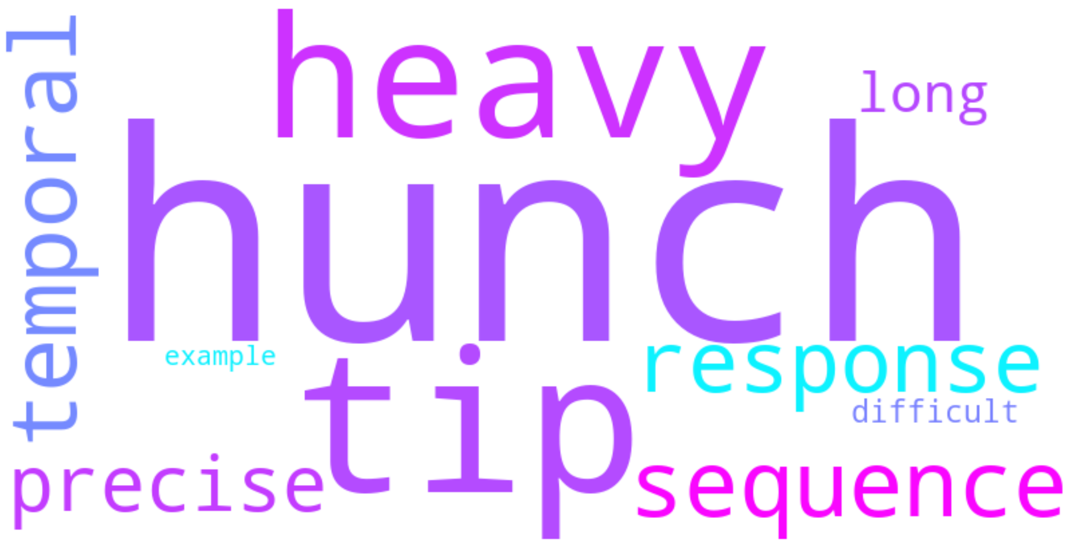

Confusion and uncertainty

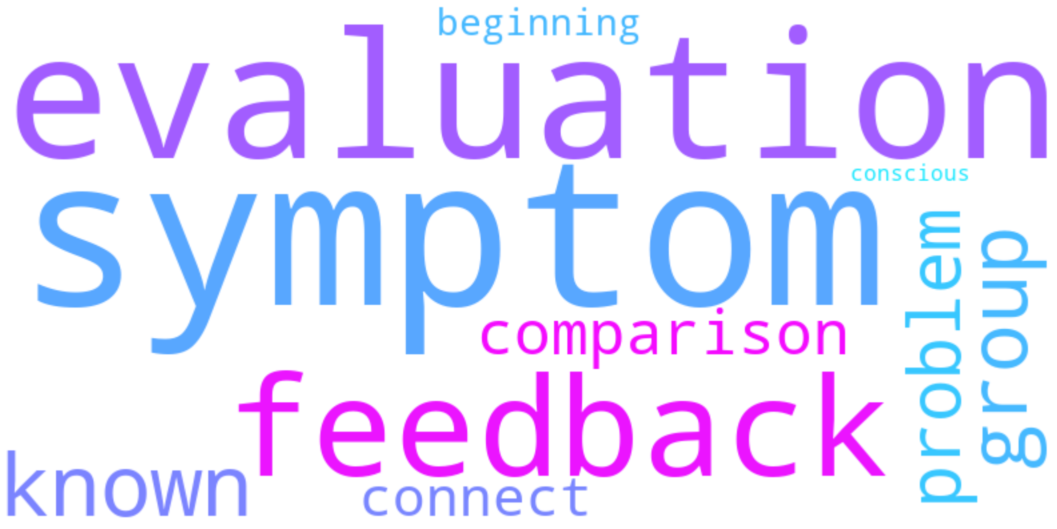

Perceptual contrasts open and closed eyes

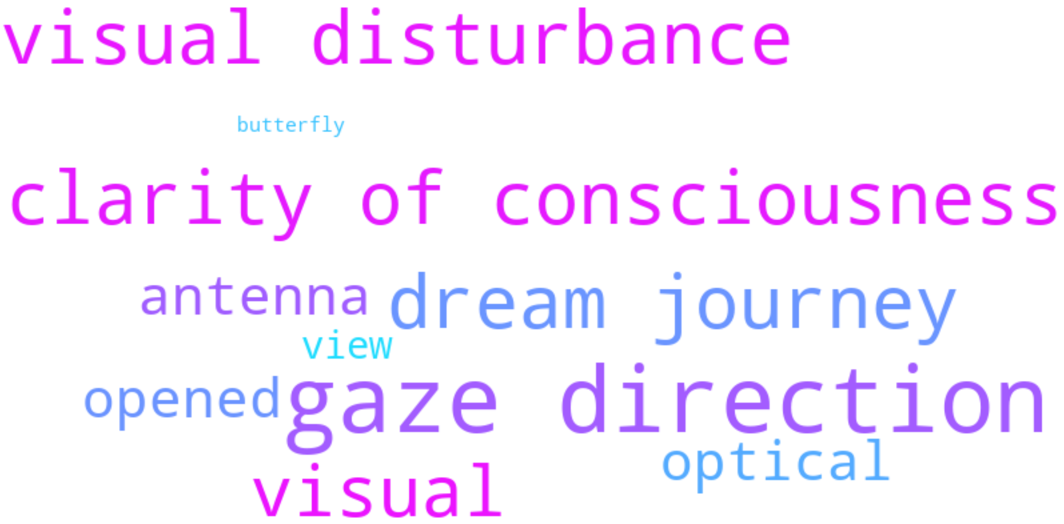

Control-acceptance dynamics

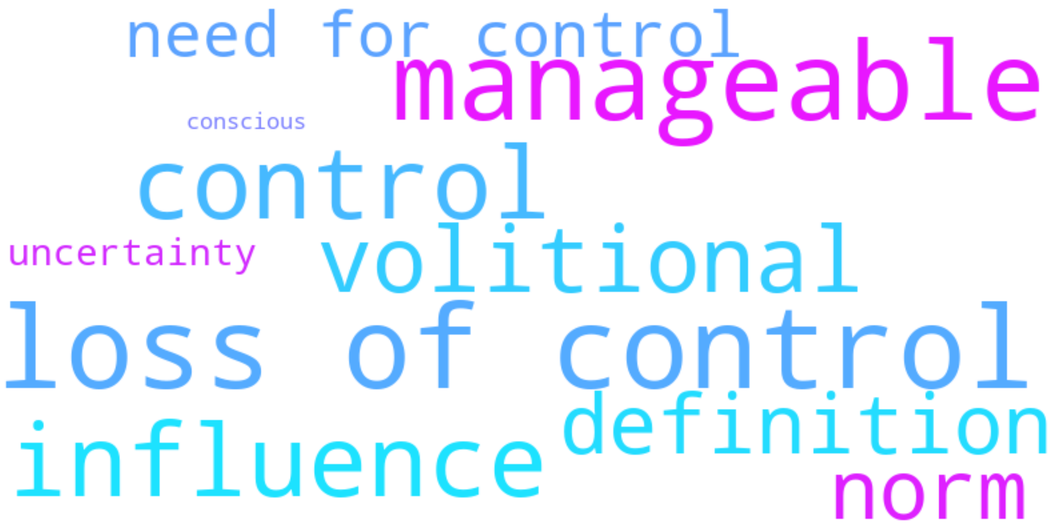

Autobiographical and past-life memories

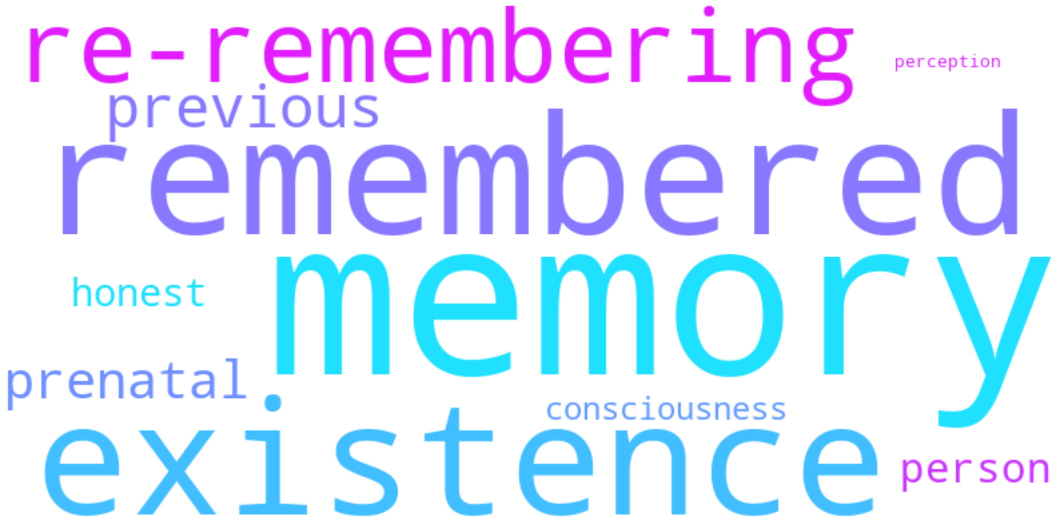

Color perception and visual synaesthesia

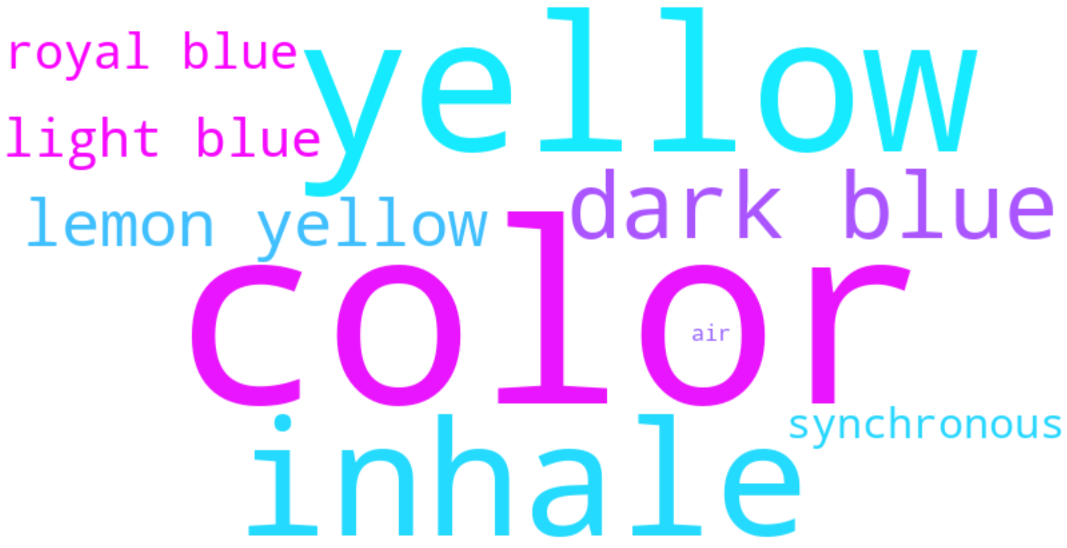

Afterglow

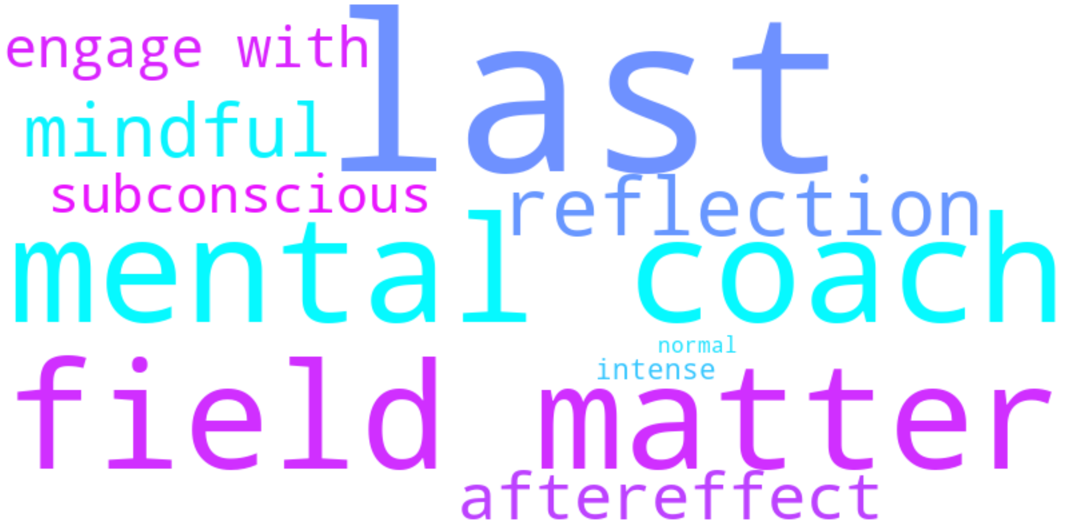

Empathy, compassion, metta

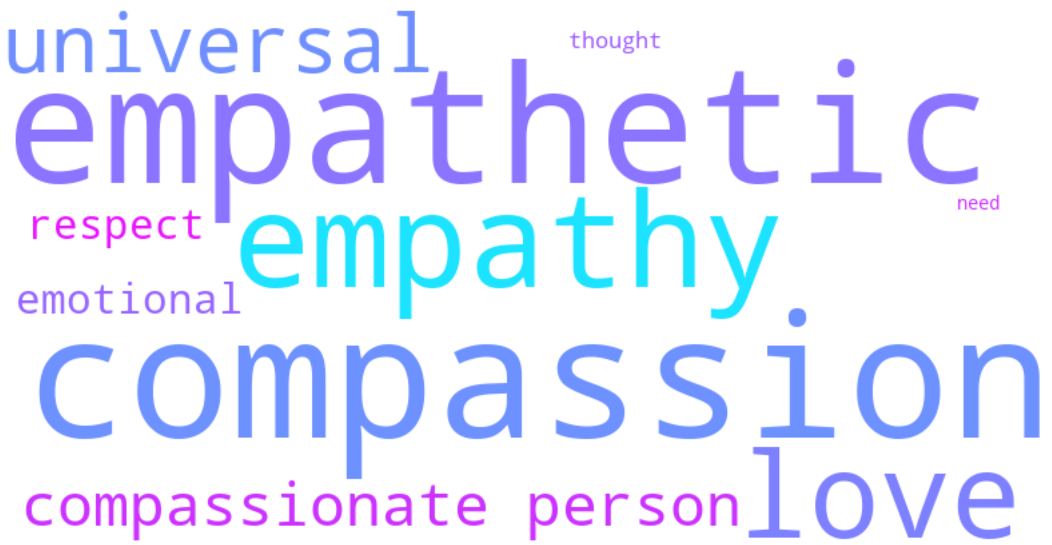

Simplicity

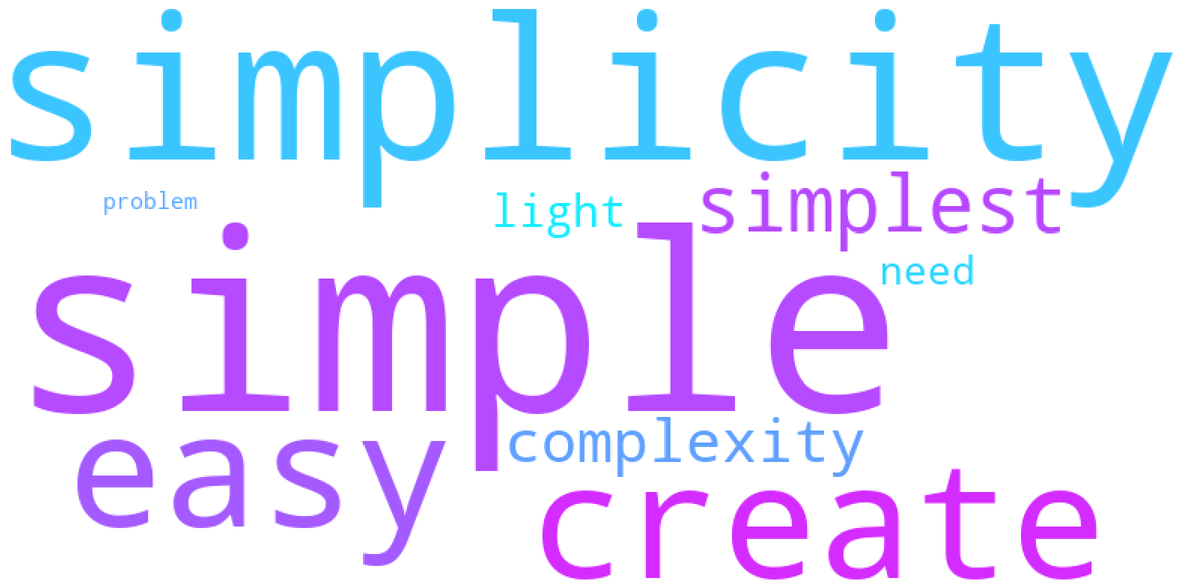

Mixed-Methods Analysis on Psychedelic-Augmented Meditation Experiences from a Randomized Controlled Mindfulness Retreat

Supplementary data XI: Word cloud representations unique keywords for all 11 distinct topics from placebo-group analysis

---

Acute phenomenology: Somatic overload, emotional release, and regulation

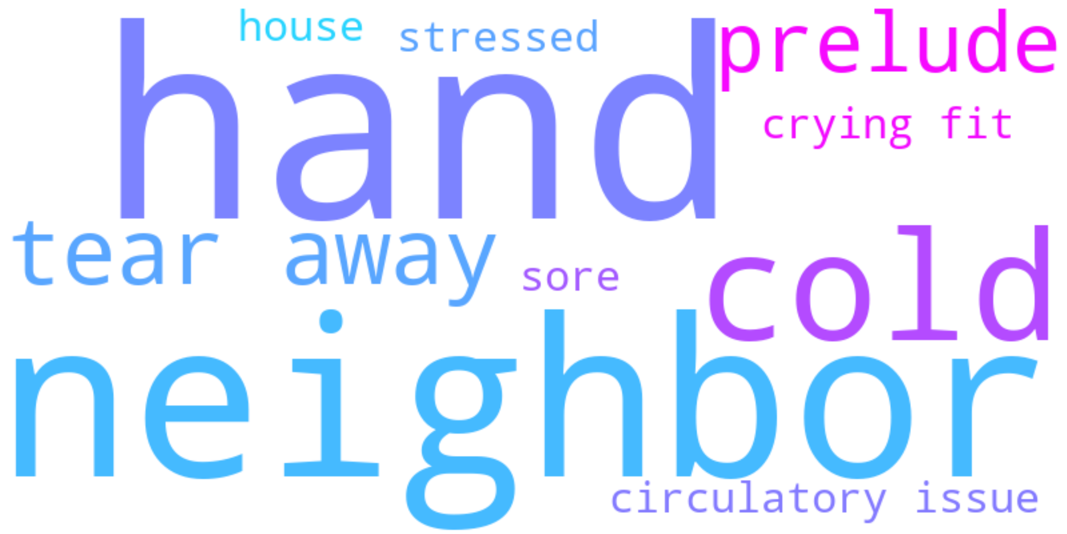

Profound positive affect and uplifted interpersonal-contextual emotional states

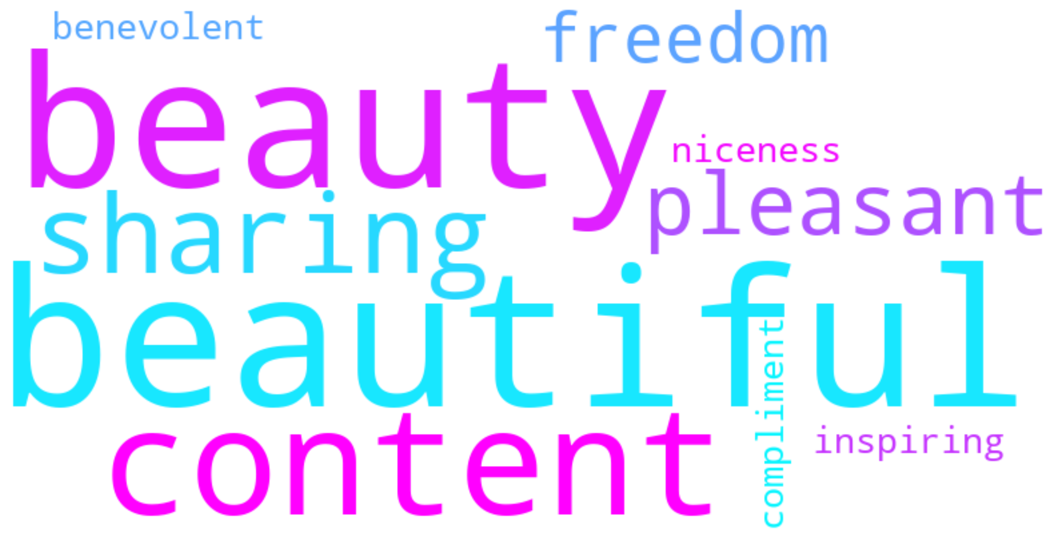

Interplay of subjective effects and contextual factors

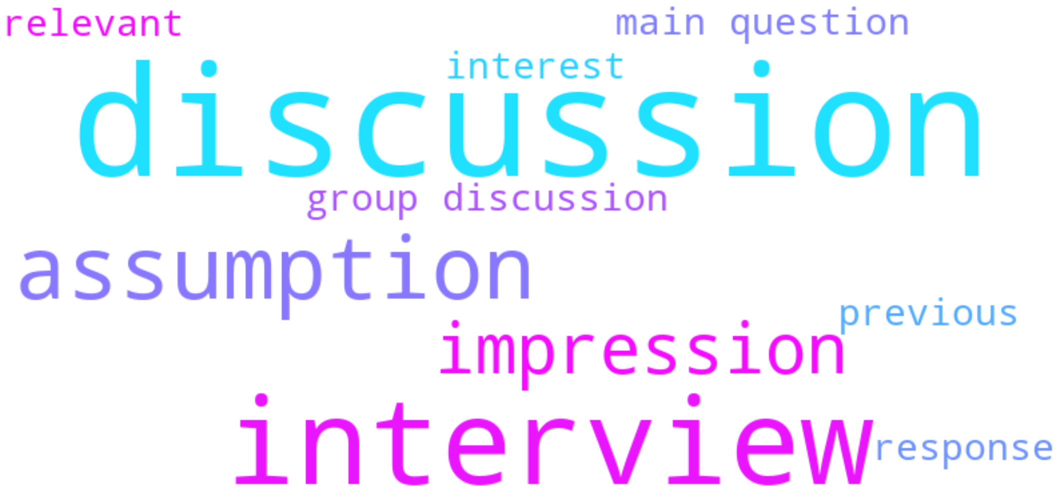

Contrasts and similarities between ordinary and enhanced meditation experience

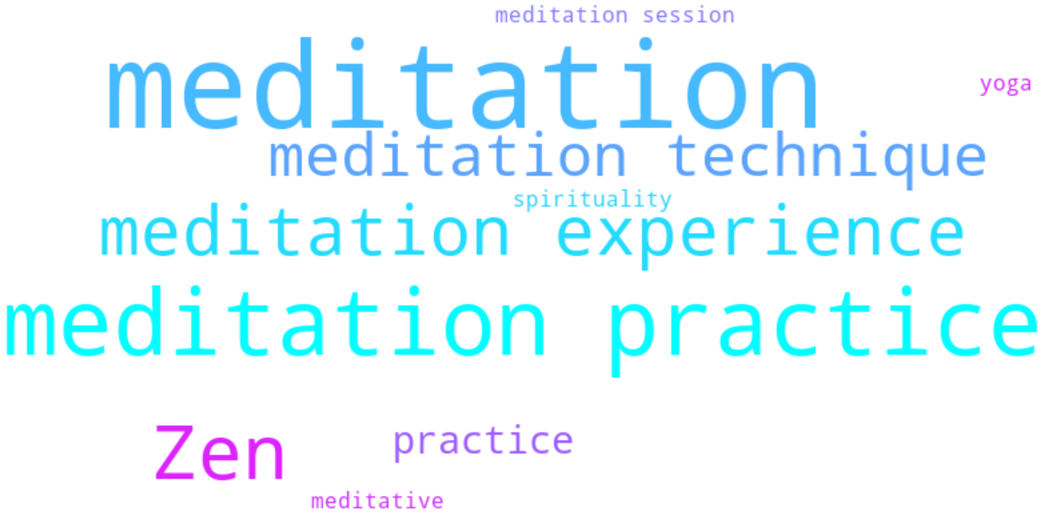

Equanimity and authentic presence

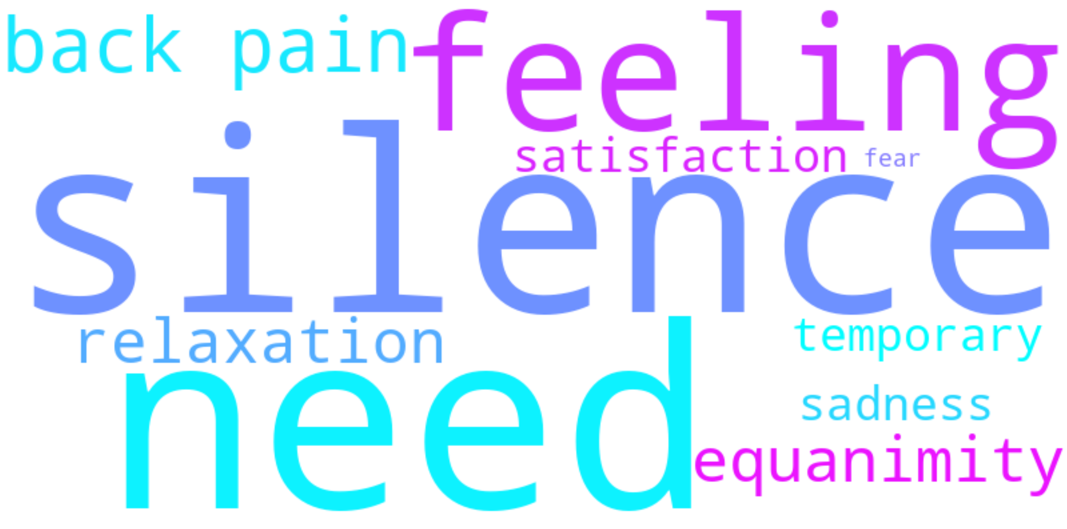

Energetic experiences and physiological sensations

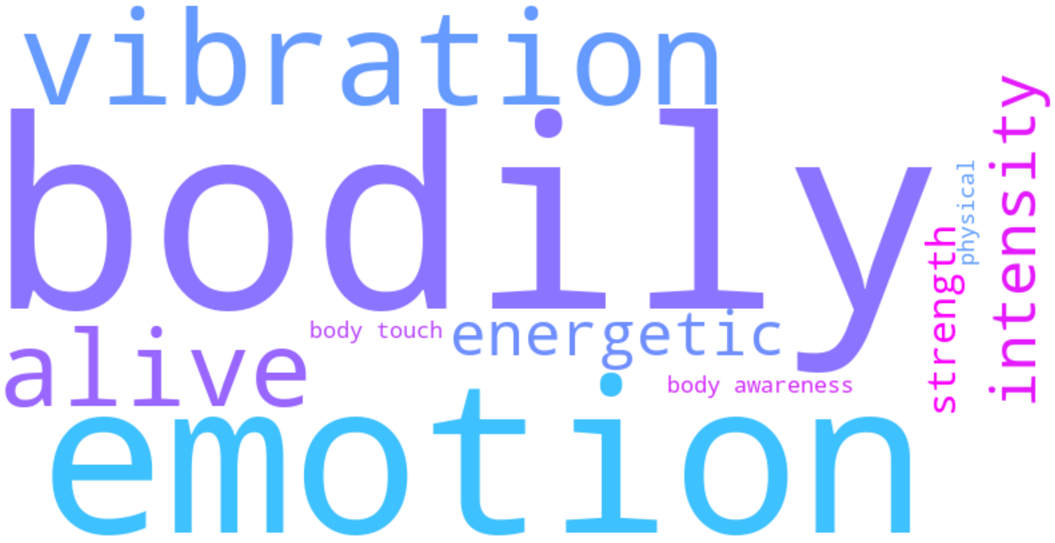

Placebo effect

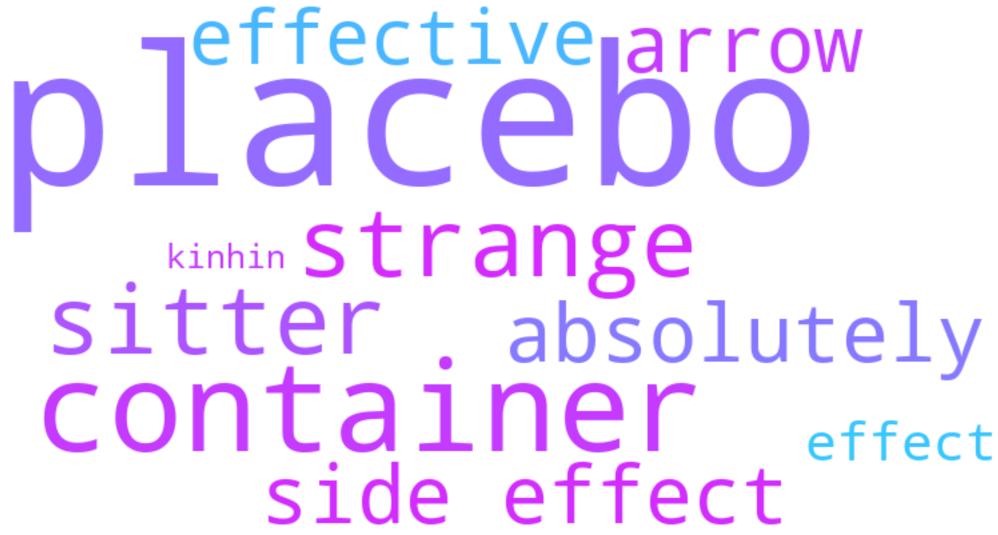

Transitions, fluctuations, and impermanence of experiential states

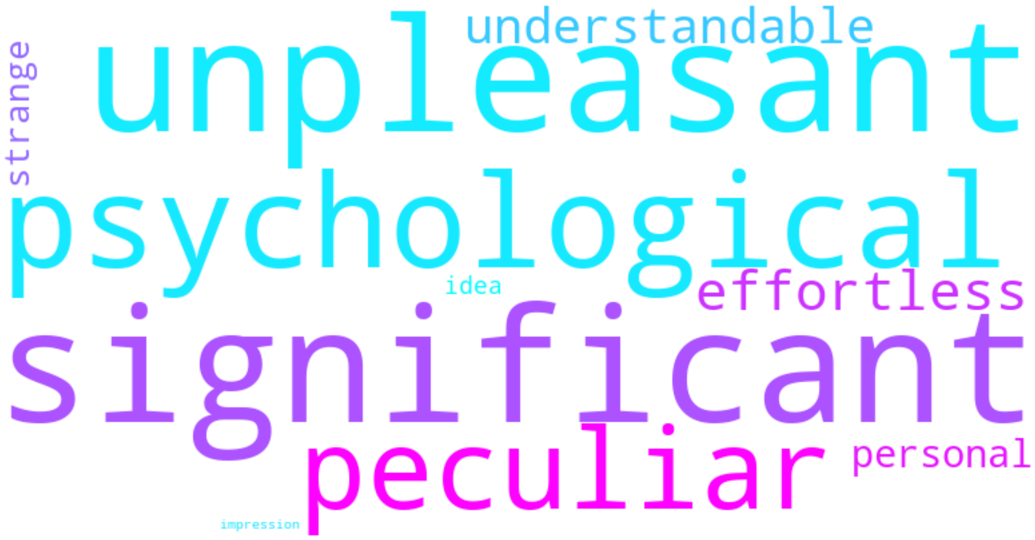

Lack of recall

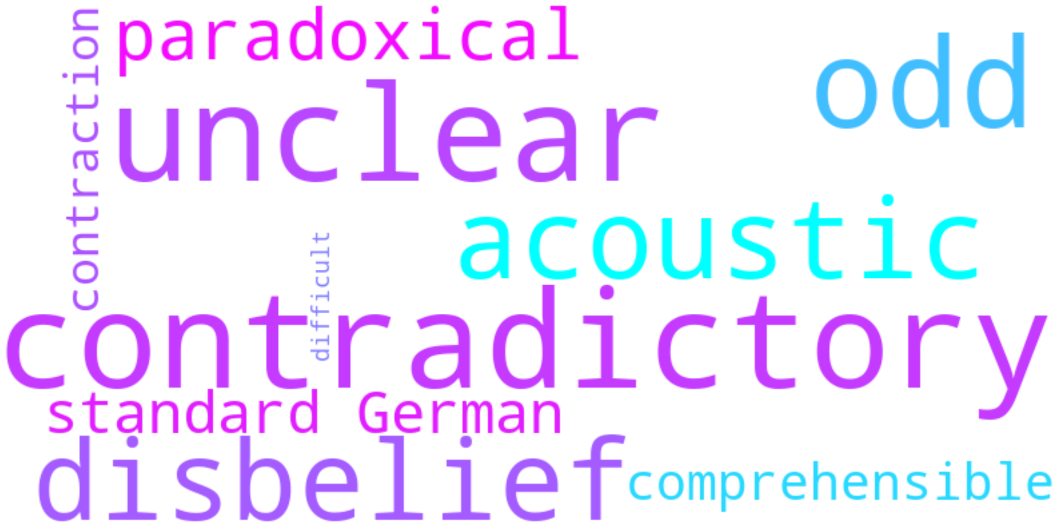

Transformative power of music

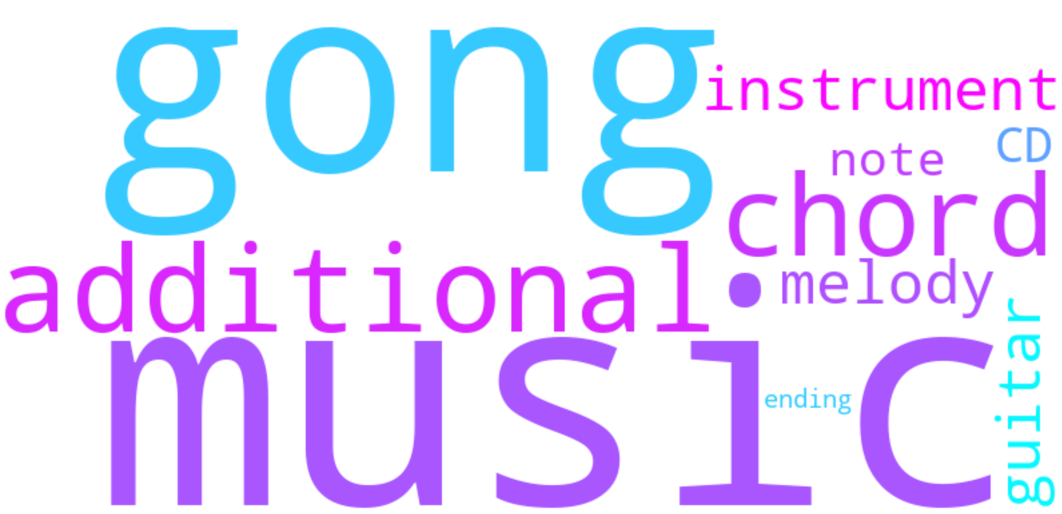

Temporal dynamics and dose-dependent effects study medication

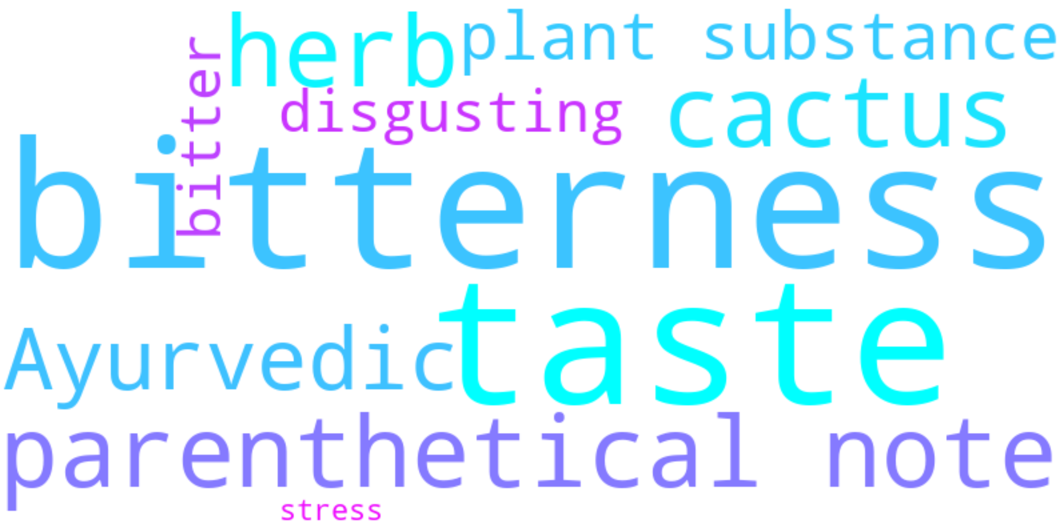

Supplement: Supplementary file 1 — Supplementary Material 1 [file 41598_2026_39261_MOESM1_ESM.pdf]
